# Proceedings of the 10th International Symposium on Veterinary Rehabilitation and Physical Therapy; and the Summit of the American Association of Rehabilitation Veterinarians; and the American College of Veterinary Sports Medicine and Rehabilitation: International Association of Veterinary Rehabilitation and Physical Therapy (IAVRPT) 2018 Abstracts

**DOI:** 10.1186/s13028-019-0439-3

**Published:** 2019-02-07

## Canine

### A1 Preliminary findings from a clinical test of a therapeutic garment for hip dysplasia

#### Lisa Bedenbaugh

##### K9Align, Irvine, CA, USA

###### **Correspondence:** Lisa Bedenbaugh (lhinerman2@aol.com)

*Acta Veterinaria Scandinavica* 2019, **61**(**Suppl 1**):A1

**Background:** Hip dysplasia is a very common orthopedic disorder in dogs, and a costly one for owners. Currently, only a few treatment strategies are available for treatment of hip dysplasia: medications, rehabilitation therapy, surgery and rigid braces/orthotics. We have developed a therapeutic garment which works to reduce effects of hip dysplasia by stimulating the proprioceptive system in a strategic way to engage the gluteal, epaxial and abdominal muscles as the dog moves, with the goal of enhancing the dynamic stability of the hip joint, thereby reducing the pain and inflammation associated with hip dysplasia.

**Materials and methods:** We conducted a preliminary clinical test of the garment on 5 dogs with varying degrees of hip dysplasia and asked the owners to have their dogs wear the garment daily for a minimum of 1 h, for a 3-week period. Owners filled out a Canine Brief Pain Index (CBPI), a validated pain scale, prior to the garment being fitted and again at the end of each week. Certified rehabilitation therapists (veterinarians or physical therapists, CCRP/T) collected objective data (hip extension ROM, thigh circumference), filled out a functional score, and videoed the dogs walking with and without the garment on at the initiation and completion of the clinical trial.

**Results:** Results from this study were favorable. All dogs demonstrated gains in hip extension ROM, thigh girth gains in 3/5 dogs, decreased lameness score in 4/5 dogs, improvement in walking distance in 3/5 dogs, better ability to climb stairs in 3/5 dogs and improvement in the CBPI noted by 4/5 owners. All dogs were accepting of the garment, and no declines in functional ability were noted by the owners during use.

**Conclusion:** Based on this limited sample trial, it appears that this garment could provide a novel approach to the treatment for hip dysplasia. A longer-term study with a larger test population is needed.

### A2 Development of a Basic Functional Neurorehabilitation Scale (BFNRS) for evaluation and monitoring of dogs with thoracolumbar injury

#### Rita Cruz^1,2^, Inês Viegas^3^, António Ferreira^4^, Artur Varejão^5^, Ângela Martins^1,2,3^

##### ^1^Hospital Veterinário da Arrábida (HVA), Azeitão, Portugal; ^2^Centro de Reabilitação Animal da Arrábida (CRAA), Azeitão, Portugal; ^3^Faculdade de Medicina Veterinária da Universidade Lusófona (ULHT), Lisboa, Portugal; ^4^Faculdade de Medicina Veterinária de Lisboa (FMV), Lisboa, Portugal; ^5^Universidade de Trás-os-Montes e Alto Douro (UTAD), Vila Real, Portugal

###### **Correspondence:** Ângela Martins (vetarrabida.lda@gmail.com)

*Acta Veterinaria Scandinavica* 2019, **61**(**Suppl 1**):A2

**Background:** Functional Neurorehabilitation (FNR) is an area of restorative neurology [1, 2, 3] which aims to achieve the functionality of neurologic patients classified according to the Modified Frankel Scale [4, 5, 6, 8, 11]. A study about the development of a Basic FNR Scale (BFNRS) for evaluation and monitoring of dogs with thoracolumbar injury (T3-L3) was performed to record the differences in patient’s recovery, direct the changes in the FNR protocols and achieve improved objectivity in the patient’s evaluation [7, 9, 10, 12, 13, 14].

**Materials and methods:** The BFNRS was applied to 34 dogs with imaging diagnosis of thoracolumbar lesion by computed tomography (CT) and magnetic resonance imaging (MRI), and classified with grade 0, 1 and 2 level of functionality according to the Modified Frankel Scale regardless breed, gender, age, body condition and etiology. At admission, the animals attempted to complete a FNR appointment, and were assessed regarding the mental state, gait, posture, postural reactions, peripheral spinal reflexes (PSR) and pain sensation. All the animals were evaluated by the BFNRS weekly while integrated in an intensive FNR program. The outcome was measured at the 4th and 8th week of the FNR program (Fig. 1). The BFNRS is a punctuation scale with 5 essential categories in the neurologic patient’s evaluation: sensorial evaluation, PSR evaluation, muscle tone evaluation, movement evaluation, and proprioception and coordination evaluation, which allows the animal’s categorization into: bad prognosis (BP), moderate prognosis (MP) and good prognosis (GP). The dogs classified with GP were integrated into a 6th category to readjust the FNR program and decrease residual neurologic deficits (Table 1). Statistical analysis was made from Microsoft Office Excel 2016 and IBM SPSS Statistics 22.0, evaluating the BFNRS score at the entrance and exit of HVA/CRAA with several variables such as gender, age, body condition, etiology and duration of FNR program.

**Fig. 1:**
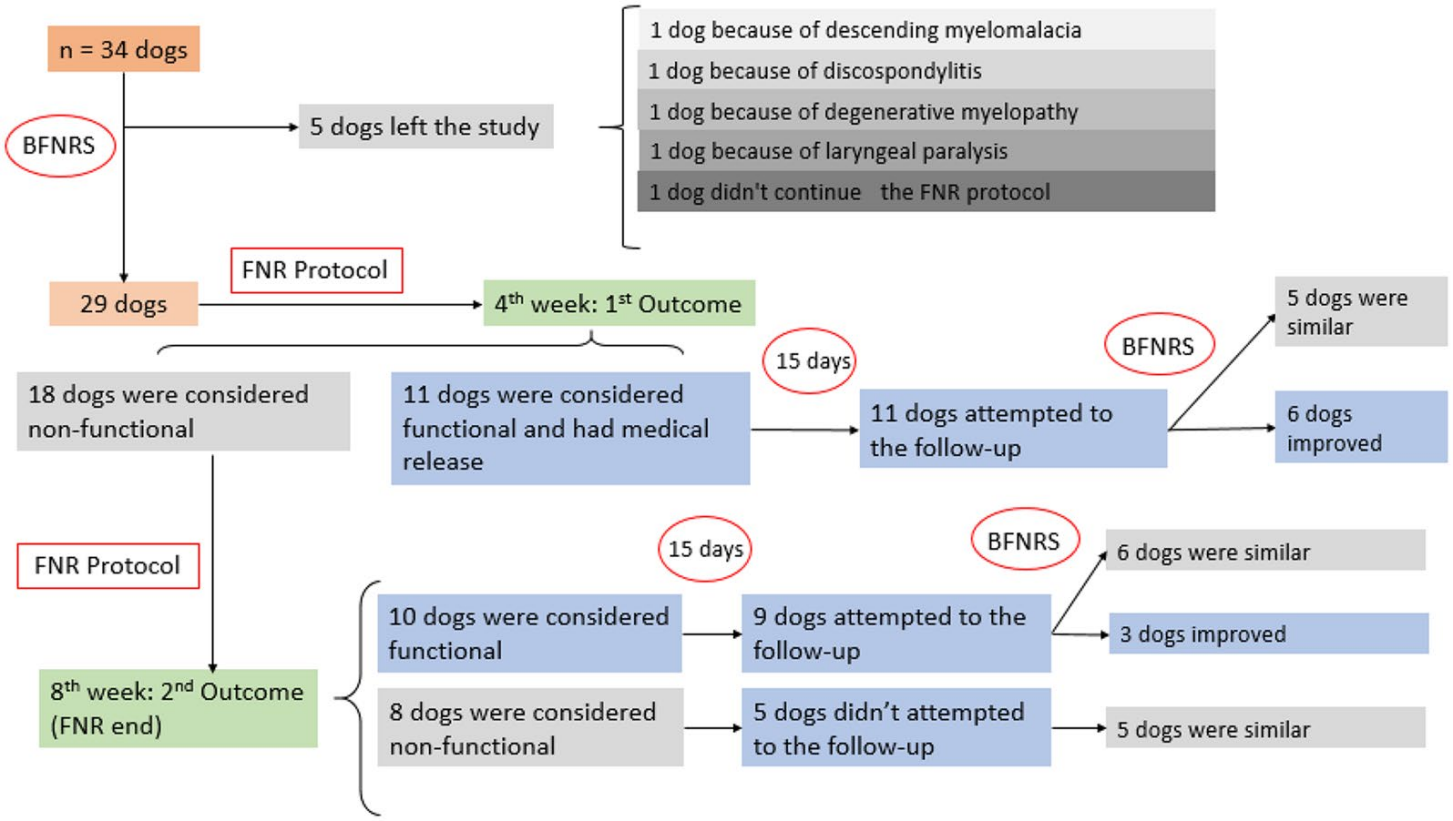
Scheme and progression of the clinical study

**Table 1:** UWTM—underwater treadmill; TM—treadmill; ROM—range of motion; PL—pelvic limbs; TL—thoracic limbs; time*—time relative to 30 steps

*Basic functional neurorehabilitation scale (BFNS) for dogs with thoracolumbar injury*		
Nociception evaluation	Deep pain sensitivity absent in the digits, perineum and vulva	0
	Deep pain sensitivity absent in the digits, and decreased in the perineum and vulva	1
	Deep pain sensitivity absent/decreased in the digits, and present in the perineum and vulva. Superficial pain sensitivity decreased	2
	Deep pain sensitivity absent/decreased in the digits, and present in the perineum and vulva. Superficial pain sensitivity present	3
	Deep pain sensitivity present in the digits, perineum and vulva	4
Spinal reflexes evaluation	Absence of spinal reflexes	0
	Patellar reflex and cranial tibial reflex decreased, but withdrawal reflex absent	1
	Patellar reflex, cranial tibial reflex and withdrawal reflex decreased	2
	Patellar reflex and cranial tibial reflex normal/increased, but withdrawal reflex decreased and crossed extensor reflex present/absent	3
	Normal spinal reflexes	4
Muscle tone evaluation	Hypotonic extensors muscles and hypotonic flexors muscles without active assisted postural standing	0
	Hypertonic extensors muscles and hypotonic flexors muscles, with active assisted postural standing but without active postural standing	1
	Spasticity of the extensors muscles and hypotonic flexors muscles, with active postural standing but passive range of motion difficult or absent ROM	2
	Hypertonic extensors muscles and hypotonic flexors muscles, with active postural standing but range of motion decreased in about 50%	3
	Normal muscle tone or slightly hypotonic flexors muscles, with active postural standing	4
Gait evaluation	Voluntary and involuntary movement absent of PL on the UWTM, TM and walking	0
	Voluntary movement absent of PL on the UWTM, TM and walking, and involuntary movement present of PL on the UWTM, TM but absent during walking	1
	Voluntary movement absent of PL on the TM and walking, but present on the UWTM, and involuntary movement present of PL on the UWTM, TM and walking, but without active postural standing and inability to stand up	2
	Voluntary movement present of PL on the UWTM and TM, but absent during walking, and involuntary movement present of PL on the UWTM, TM and walking, with active postural standing but inability to stand up	3
	Voluntary and involuntary movement present of PL on the UWTM, TM and walking, with active postural standing and ability to stand up	4
Proprioception and locomotor coordination evaluation	Coordination between PL and TL < 10% of the time*; ± knuckling	0
	Coordination between PL and TL between 10 and 25% of the time*; ± knuckling	1
	Coordination between PL and TL between 25 and 50% of the time*; without knuckling	2
	Coordination between PL and TL between 50 and 75% of the time*; without knuckling	3
	Coordination between PL and TL > 75% of the time*; without knuckling	4
Poor prognosis (0–9)	Fair prognosis (10–14)	Good prognosis (15–20)
*Subclass for patients with good prognosis*		
Gait defects evaluation	Wide base walking, crossing and dragging of the PL > 75% of the time*	0
	Wide base walking, crossing and dragging of the PL between 50 and 75% of the time*	1
	Wide base walking, crossing and dragging of the PL between 25 and 50% of the time*	2
	Wide base walking, crossing and dragging of the PL between 5 and 25% of the time*	3
	Wide base walking, crossing and dragging of the PL < 5% of the time*	4

**Results:** In the study, there was a significant relation between the BFNRS scores and the animal’s functionality (P < 0.01), significant relation between BFNRS scores and the FNR period (P < 0.01), and a positive correlation between the entrance’s score and the exit’s score, in addition to other interesting results.

**Conclusion:** The BFNRS allowed the evaluation of all the animals in a brief period (40 to 60 min), for fast perception of individual’s sensory and motor deficits, and the rapid change of the FNR’s protocols, which makes the BFNRS a possible operational tool for all FNR centers, according to the restorative neurology’s guidelines.

**References**

1. Angeli CA, Edgerton VR, Gerasimenko YP, Harkema SJ. Altering spinal cord excitability enables voluntary movements after chronic complete pralysis in humans. Brain. 2014;137:1394–409.
2. Dietz, V. Neuronal plasticity after a human spinal cord injury: positive and negative effects. Exp Neurol. 2012;235:110–5.
3. Edgerton VR, Roy RR. Activity-dependent plasticity of spinal locomotion: implications for sensory processing. Exerc Sport Sci Rev. 2009;37:171–8.
4. Courtine G, Gerasimenko Y, van den Brand R, Yew A, Musienko P, Zhong H, et al. Transformation of nonfunctional spinal circuits into functional states after the loss of brain input. Nat Neurosci. 2009;12:1333–42.
5. Duysens J, Crommert HW. Neural control of locomotion; the central pattern generator from cats to humans. Gait Posture. 1998;7:131–41.
6. Gerasimenko Y, Gorodnichav R, Machueva E, Pivovarova E, Semyenov D, Savochin A, et al. Novel and direct access to the human locomotor spinal circuitry. J Neurosci. 2010;30:3700–8.
7. Gerasimenko Y, Gorodnichev R, Puhov A, Moshonkina T, Savochin A, Selionov V, et al. Initiation and modulation of locomotor circuitry output with multi-site transcutaneous electrical stimulation of the spinal cord in non-injured humans. J Neurophysiol. 2014;113:834–42.
8. Grasso R, Ivanenko YP, Zago M, Molinari M, Scivoletto G, Castellano V, et al. Distributed plasticity of locomotor pattern generators in spinal cord injured patients. Brain. 2004;127:1019–34.
9. Hubli M, Dietz V. The physiological basis of neurorehabilitation—locomotor training after spinal cord injury. J Neuroeng and Rehabil. 2013;10:1–8.
10. Krueger E, Magri LM, Botelho AS, Bach FS, Rebellato CL, Fracaro L, et al. Low-intensity electrical stimulation and stem cells in a dog with acute spinal cord injury. In: Proceedings of the Thirteeth International Association of Science and Technology for Development (IASTED) International Conference on Biomedical Engineering 2017; Innsbruck.
11. Lavrov I, Musienko PE, Selionov VA, Zdunowski S, Roy RR, Edgerton VR, et al. Activation of spinal locomotor circuits in the decerebrated cat by spinal epidural and/or intraspinal electrical stimulation. Brain Res. 2014;1600:84–92.
12. Lewis MJ, Howard JF, Olby NJ. The relationship between trans-lesional conduction, motor neuron pool excitability and motor function in dogs with incomplete recovery from severe spinal cord injury. J Neurotrauma. 2017;34:2994–3002.
13. Musienko P, Brand RV, Marzendorfer O, Roy RR, Gerasimenko Y, Edgerton VR, et al. Controlling specific locomotor behaviors through multidimensional monoaminergic modulation of spinal circuitries. J Neurosci. 2011;31:9264–78.
14. Olby NJ, Smith DT, Humphrey J, Spinapolice K, Parke N, Mehta PM, et al. Pharmacokinetics of 4-aminopyridine derivatives in dogs. J Vet Pharmacol Therap. 2009;32:485–91.

### A3 Recovery of walking after an IVDD without DPP with adipose derived mesenchymal stem cells

#### Yolanda Elbertse, Peter Hundepool

##### Rehab and Pain Centre for Animals, Rotterdam, Netherlands

###### **Correspondence:** Yolanda Elbertse (sterkliniek@live.nl)

*Acta Veterinaria Scandinavica* 2019, **61**(**Suppl 1**):A3

**Background:** A 2-year old female English Staffordshire Terrier had surgery for intervertebral disc disease (IVDD) at L1–L2, leaving her with no deep pain perception (DPP).

**Materials and methods:** The dog received 1 year of underwater treadmill (UTM) at a PT before being transferred to our center for more extensive rehab (Table 1). After improving her muscle stamina (Table 2) we started ground walking. Initially, the feet had to be placed forward by hand. She voluntarily protracted her foot 26 weeks later, with tactile hamstring cuing.

**Table 1:** Interventions used during extensive rehab

Modality	Details	Other
Laser	20 J/cm^2^, location T11-L6	
Acupuncture	Dorsal branches spinal cord T11-L3 Sciatic nerve at greater trochanter Tibial and fibular nerve above the achilles heel Interdigital	After 4 times the placing of the needles at the dorsal branches was stopped
Weight shifting	Ground > balancing board > peanut or donut	Also done daily at home
Ground walking exercise	First with complete placing of the hind feet Later with just triggering the hamstrings	Taping helped decrease muscle spasms

**Table 2:** Results before and after extensive rehab

	After 1 year only UTM	After 1 year extensive rehab
Hind leg R	22.3 cm	24.5 cm
Hind leg L	24.0 cm	26 cm
Standing on 4 feet	< 2 min	> 15 min
Three-legged stance	Not possible	Few seconds
Protracting hind leg	Never	Only when triggered
Getting onto hind feet	Never	< 20% when asked
Results	Olby score 1	Olby score 3

The rehab treatment improved her Olby score from 1 to a 3 [1]. The dog moved her hind legs during sleep and swimming. Table 2 shows her accomplishments. However, 6 additional months of extensive rehab did not improve her scoring beyond Olby 3. This was considered too poor of a performance outcome, thus adipose derived stem cell treatment was suggested.

Under general anesthesia fat was harvested at the falciformic ligament and the inguinal region. It was processed with the in-house-kit of Medivet. One ml was added to Nano whiskers and injected in the epidural space. The remainder (2 mL) was given intravenously.

**Results:** Three weeks post-injection, the dog showed neurological improvements (Table 3).

**Table 3:** Results after stem cell treatment

1st ADMCS	2 weeks	Waning atopy		Olby score 3
	3 weeks	Less force required to hold the dog up during assisted ground walking Better weight shifting stability		
	4 weeks	Wagged tail Flexor reflex hind legs L and R		Olby score 5
	6 weeks	Panniculus reflex up to L4 Pain perception at L4 Lateral skin sensation at the hock joint Less hyper flexor reflex hind legs Spinal walking for a few steps without assistance	Adding LTM, laser upped to 40 J/cm2	
	3 months	Walked up a step with hind legs		Olby score 7
	5 months	45 min walk without assistance		Olby score 12
2nd ADMSC	2 weeks	Gets on feet in house independently Reduced crossing of hind legs		Olby score 13

After one treatment the dog could walk for 45 min, though she continued to cross the hind legs or walk ‘ataxic-like’. During daily walks wheelchair usage was continued to prevent falls, though she walked on all four feet. At home she did not voluntarily utilize all four feet and continued dragging herself forward. After 10 months a second stem cell treatment was suggested. This time the stem cells were given intrathecally and through IV. Following this treatment, she began to walk around the house instead of dragging herself. Outdoors she made less gait deviations.

**Conclusion:** This study has a solid historical control. It is clear that the final results involving improved gait and hind limb muscle recruitment of the dog were achieved following the addition of stem cells treatment to the rehabilitation intervention. Further research is needed to determine if an intrathecal stem cell injection yields varied results from an epidural injection and to determine the best interval between treatments.

**Reference**

1. Olby NJ, Lim JH, Babb K, Bach K, Domaracki C, Williams K, et al. Gait scoring in dogs with thoracolumbar spinal cord injuries when walking on a treadmill. BMC Vet Res. 2014;10:58.

### A4 Comparison of a commercially available consumer mobile movement analysis application with a 3-D kinematic gait analysis system

#### Jose L Guevara, Emily A Liles, Joseph P Weigel, Darryl L Millis

##### Department of Small Animal Clinical Sciences, University of Tennessee College of Veterinary Medicine, Knoxville, TN, USA

###### **Correspondence:** Darryl L Millis (boneplate@aol.com)

*Acta Veterinaria Scandinavica* 2019, **61**(**Suppl 1**):A4

**Background:** Kinematic gait evaluation is increasingly used to assess the effects of exercises on joint motion and the efficacy of treatments for musculoskeletal conditions [1, 2]. Recently, several low-cost consumer applications (apps) have become available. The apps measure motion in 2-D, but aim to improve decision making using an economical, available, and easy to use kinematic gait analysis tool. However, the accuracy of these apps has not been evaluated in dogs. The purpose of this study was to compare a consumer movement analysis app to a 3-D kinematic gait analysis system (KGAS). We hypothesized that the two gait analysis systems not be significantly different in the sagittal plane in trotting dogs.

**Materials and methods:** Reflective spheres were attached to anatomic landmarks on ten dogs. Dogs were trotted in a test area while four infrared cameras and an infrared digital camcorder captured kinematic data. Five trials of each side of the dogs were obtained. Images from the infrared cameras were recorded in a KGAS program (Peak Motus, Centennial, CO, USA). Images from the infrared digital camcorder were uploaded to the app (Simi Move, Germany). Maximum flexion, extension, and total range of motion (ROM) were calculated for each trial of the shoulder, elbow, hip, and stifle using the KGAS and the app. Mean joint angles were calculated from the five trials. Total joint ROM was calculated for each trial by subtracting the flexion from the extension angle. Values obtained for the mobile app were compared to the KGAS by paired *t* test with P values < 0.05 considered significant.

**Results:** Mean joint angles obtained on the app were not significantly different (P > 0.702) from those generated by the KGAS (Figs. 1, 2). 97.5% of the angles measured on the mobile app deviated < 5% from KGAS values, with most of those deviating more than 5% measured during flexion. Although mean ROM calculated by the app exhibited greater deviation than expected, ROM values calculated on the app were not significantly different than actual values (p > 0.283).

**Fig. 1:**
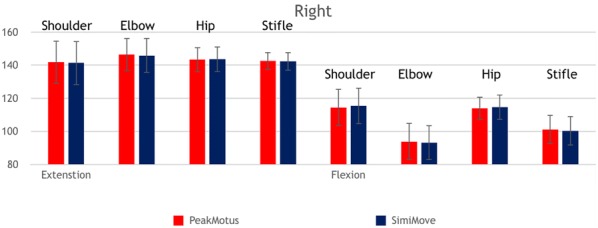
Comparison of extension and flexion angles of the shoulder, elbow, hip and stifle between KGAS and an app for the right side of trotting dogs

**Fig. 2:**
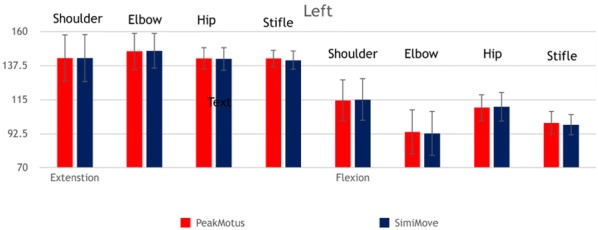
Comparison of extension and flexion angles of the shoulder, elbow, hip and stifle between KGAS and an app for the left side of trotting dogs

**Conclusion:** The app performed well and may be an inexpensive method to obtain joint angles, with 97.5% of the measurements from the mobile app accurately identifying maximum joint angles within 5% of the KGAS values. Limitations of this study included artifact from images on the infrared digital camcorder and skin marker movement artifacts during ambulation. Additional studies are required to determine the best protocols and use of this technology in clinical settings.

**References**

1. DeCamp CE. Kinetic and kinematic gait analysis and the assessment of lameness in the dog. Vet Clin North Am Small Anim Pract. 1997;27:825–40
2. Gilette R. Gait analysis. In: Millis DL, Levine D, Taylor RA, editors. Canine rehabilitation and physical therapy. St. Louis (MO): WB Saunders; 2004. p. 201–10.

### A5 Kinetic and temporospatial gait parameters in dogs: reproducibility of an instrumented pressure-sensitive canine treadmill

#### Kirsten Haeusler^1^, Doro Braun^1^, Nah-Chieh Liu^2^, Matthew James Allen^2^

##### ^1^Zentrum für Tierphysiotherapie, Stuttgart, Germany; ^2^Department of Veterinary Medicine, University of Cambridge, Cambridge, UK

###### **Correspondence:** Kirsten Haeusler (info@dr-haeusler.com)

*Acta Veterinaria Scandinavica* 2019, **61**(**Suppl 1**):A5

**Background:** Objective, reliable and validated gait analysis is rarely used routinely in clinical practice because it is time-consuming and rarely cost-effective. The purpose of this study was to assess the validity and reliability of a pressure sensor matrix equipped stationary treadmill (CanidGait^®^, Zebris) for measuring gait characteristics in dogs of various sizes and weights. We hypothesized that the system would provide reliable measures of gait parameters in dogs.

**Materials and methods:** 12 client-owned dogs were measured while walking on the CanidGait^®^ system at a constant velocity of 3 km/h. Body weight of the dogs (kg), temporospatial (stride length, cadence) and kinetic data (pressure distribution) were recorded for all four limbs. Short-term (intra-session) reproducibility of the measurements was determined by comparing two measurements collected in the span of 2 h on the same day. Long-term (inter-session) reproducibility was determined by comparing baseline measurements on 1 day with a second set of measurements collected not less than 4 days later. Reproducibility between either the two measurements on the same day, or the two measurements on different days, were assessed using intraclass correlation coefficient (ICC) and ratios of difference. Statistical significance was determined at P < 0.05.

**Results:** The dogs evaluated in this study weighed between 12.7 kg and 31.2 kg. ICC values for intra-session and inter-session comparisons of pressure distribution ranged from 0.959 to 0.992 and 0.917 to 0.978 respectively. ICC values for step length and cadence were also excellent (> 0.76 in all cases). Results for swing and stance phase lengths were more variable (0.663 to 0.942). Overall ratios of differences were lower for comparisons from the same day, but in all cases ratios of differences were below 5%.

**Conclusion:** The CanidGait^®^ system demonstrates excellent intra- and inter-session reproducibility. A ratio of difference of 5% or less indicates the validity of this system for objective gait analysis in dogs, both as a means of assessing lameness but also as an objective method of documenting the effectiveness of new surgical techniques and post-operative rehabilitation protocols.

### A6 Lower back pain: positing a novel cause and treatment for canine urethral sphincter mechanism incompetency

#### David Lane^1^, Sarah Hill^2^

##### ^1^Points East West Veterinary Services, Garibaldi Highlands, BC, Canada; ^2^Department of Military Psychology & Leadership, Royal Military College of Canada, Kingston, ON, Canada

###### **Correspondence:** Sarah Hill (dlane@pointseastwest.com)

*Acta Veterinaria Scandinavica* 2019, **61**(**Suppl 1**):A6

**Background:** Canine urethral sphincter mechanism incompetency (USMI) is the most common cause of urinary incontinence in dogs [1, 2]. Sex, age when neutered, bladder position, tail docking, breed, body weight, obesity, and urethral length are all risk factors, and the only recognized non-surgical treatment is ongoing medication [1–3]. To the authors’ knowledge, this paper is the first to posit that palpable lower back pain (LBP) is a potential cause or risk factor for USMI. If LBP is a cause of USMI, it follows that resolution of LBP should result in a reduction or resolution of USMI symptoms. We hypothesize that some dogs presenting with a history of urinary incontinence and concurrent symptoms of LBP, and whom receive treatment for LBP, will demonstrate a corresponding reduction in USMI symptoms.

**Materials and methods:** This retrospective study examined the outcomes of 19 dogs presenting with concurrent LBP and urinary incontinence that were treated for LBP exclusively (Group A). Further, as part of a larger research project, blinded outcomes were recorded on 5 dogs with a history of urinary incontinence and concurrent LBP, treated for LBP alone (Group B). Group A responses were divided into none, partial, or complete, based on the frequency or volume of urine leaked following treatment. The frequency and volume of incontinence episodes were compared pre and post treatment for Group B.

**Results:** Four Group A dogs (21.1%) showed no improvement in USMI symptoms. Seven (36.8%) showed partial improvement. Of the dogs demonstrating complete resolution, 0% relapsed within 6 months, 2 (10.5%) relapsed between 6 and 12 months, 2 (10.5%) relapsed after 12 months, and 2 (10.5%) permanently responded. Two dogs (10.5%) had mixed results, with one relapsing after 5 months then permanently resolving, and 1 relapsing within both the short and medium-term time frames (< 6 months, and 6–12 months). Eighty percent of Group B dogs showed a reduction in frequency of incontinence, and in voided urine volume.

**Conclusion:** Group A results indicate that treating LBP may reduce or eliminate the symptoms of USMI in 78.9% of dogs. Group B dogs also showed an average drop in both the frequency and volume of urine leaked in the short term following treatment. This suggests that LBP is a risk factor or potential cause of USMI. Further prospective, blinded, randomized, and controlled research is required to substantiate this observation.

This paper complies with the NIH guidelines for Humane Care and Use of Animals.

**References**

1. Reichler I, Hubler M. Urinary incontinence in the bitch: an update. Reprod Domest Anim. 1987;49:75–80.
2. Chew DJ, DiBartola SP, Schenck PA. Disorders of micturition and urinary incontinence. In: Chew DJ, DiBartola SP, Schenck PA, editors. Canine and feline nephrology and urology. 2nd ed. Saint Louis (MO): W.B. Saunders; 2011. p. 409–33
3. Power SC, Eggleton KE, Aaron AJ, Holt PE, Cripps PJ. Urethral sphincter mechanism incompetence in the male dog: importance of bladder neck position, proximal urethral length and castration. J Small Anim Pract. 1998;39:69–72.

### A7 Thermographic evaluation of the duration of cryotherapy effect in dogs undergoing tibial plateau leveling osteotomy (TPLO) surgery

#### Jack A. Lee^1^, Betsy Phillips^1^, Marti G. Drum^1^, Henry S. Adair, III^2^, Darryl L. Millis^1^

##### ^1^Small Animal Clinical Sciences, College of Veterinary Medicine, University of Tennessee, Knoxville, TN, USA; ^2^Large Animal Clinical Sciences, College of Veterinary Medicine, University of Tennessee, Knoxville, TN, USA

###### **Correspondence:** Darryl L. Millis (Boneplate@aol.com)

*Acta Veterinaria Scandinavica* 2019, **61**(**Suppl 1**):A7

**Background:** Cryotherapy is commonly applied to patients after tibial plateau leveling osteotomy (TPLO). Although studies have assessed duration of effect of cryotherapy in the canine stifle, few studies have examined this in the clinically relevant context of a post-operative stifle using noninvasive techniques [1–3]. Thermal imaging is a non-invasive method to measure superficial skin temperature and has been used in both human and veterinary medicine for a variety of indications, including to assess duration of cooling after cryotherapy. However, duration of cooling has not been assessed by thermography in the inflammatory setting of a post-operative canine stifle. The purpose of this study was to evaluate the use of thermal imaging to determine changes in surface temperature following TPLO, cryotherapy, and during the rewarming period. We hypothesized that thermal imaging would be useful to assess changes in limb surface temperature, and that the pattern of cooling and rewarming would be similar to invasive methods of measurement.

**Materials and methods:** Eighteen client-owned dogs undergoing TPLO surgery were enrolled after obtaining consent. The day after surgery, dogs were placed in a draft-free room with constant temperature and bandages were removed from limbs. Thermographic images were made of the lateral, cranial, and medial aspects of both stifles using a Med2000 thermal imaging camera. Thermal images were taken of the limb after bandage removal until temperature equilibrated 60 min after bandage removal. Cryotherapy was then applied for 20 min using bags of crushed ice. Thermographic images of the sites were obtained 15, 30, 45, 60, 90, 120, 150, 180, 210, and 240 min after cryotherapy. A one-way repeated measures analysis of variance (ANOVA) was used to evaluate the treatment effect across time at each site. Fisher’s least significant difference mean separation was used as post hoc test. The significance level was set at P < 0.05.

**Results:** Surface temperatures of all sites of the stifle were significantly lower after cryotherapy (P < 0.001, Fig. 1). Rewarming began within 15 min after cryotherapy, and by 45 min, temperature returned to equilibrium temperatures for all views of the stifle.

**Fig. 1:**
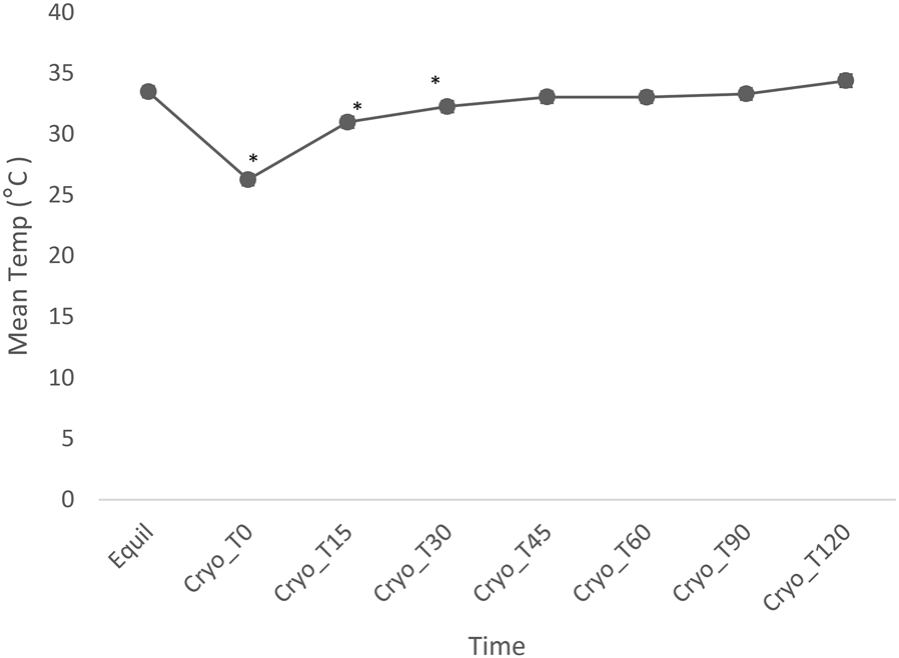
Mean temperatures of the medial aspect of the stifle, immediately prior to cryotherapy, after 20 min of cryotherapy, and during rewarming for 2 h. *Indicates significant difference from Equil (P < 0.05)

**Conclusion:** This rewarming pattern was similar to previous thermister derived rewarming data [1], although rewarming occurred sooner in these post-operative patients. These results suggest that more frequent application of cryotherapy may be indicated post-operatively. However, additional studies are needed to evaluate the relationship between surface temperatures measured with thermal imaging and temperatures of deeper tissues.

**References**

1. Vannetta M, Millis D, Levine D, Adair S, Schwartz P, Hicks D, et al. The effects of cryotherapy on in vivo skin and muscle temperature, and intramuscular blood flow. J Orthop Sports Phys Ther. 2006;36:A47.
2. Rexing J, Dunning D, Siegel A, Knap K, Werbe B. Effects of cold compression, bandaging, and microcurrent electrical therapy after cranial cruciate ligament repair in dogs. Vet Surg. 2010;39:54–8.
3. Drygas K, McClure S, Goring R, Pozzi A, Robertson SA, Wang C. Effect of cold compression therapy on postoperative pain, swelling, range of motion, and lameness after tibial plateau leveling osteotomy in dogs. J Am Vet Med Assoc. 2011;238:1284–91.

### A8 Evaluation of forces produced by therapeutic elastic resistance bands of various lengths and colors: a biomechanical study

#### Daniel McCarthy^1^, Ellis Wright^2^, Pierre-Yves Mulon^3^, Darryl Millis^1^

##### ^1^CARES Center, The University of Tennessee College of Veterinary Medicine, Knoxville, TN, USA; ^2^Biomedical and Diagnostic Sciences, The University of Tennessee College of Veterinary Medicine, Knoxville, TN, USA; ^3^Large Animal Clinical Sciences, The University of Tennessee College of Veterinary Medicine, Knoxville, TN, USA

###### **Correspondence:** Daniel McCarthy (dmccart6@utk.edu)

*Acta Veterinaria Scandinavica* 2019, **61**(**Suppl 1**):A8

**Background:** Elastic resistance bands (ERB) have been used in human and veterinary rehabilitation to increase muscle strength. ERB are used in human physical therapy to improve muscle strength and achieve earlier return to function after surgery [1, 2], in elderly adults [3], osteoarthritis [4], and stroke injury [5]. Limited data have been published using ERB for veterinary conditions, but they are commonly used for neurologic and orthopedic disease. Studies in veterinary rehabilitation regarding forces generated using different lengths of bands, different stiffnesses, and by stretching to different lengths are lacking. Previous studies have published data for both 100 and 200% elongation values; however, these values may not be applicable in dogs, especially considering the stride length of dogs [6]. The aims of this study were to determine the forces produced with different stiffnesses of colored ERB at 1.25, 1.5 and 1.75 times the original length, and to determine the effects of using two different lengths of ERB (10 cm and 40 cm). We hypothesized that the ERB would behave in linear fashion and that shorter ERB, stiffer ERB, and greater elongation values would generate higher forces.

**Materials and methods:** Five replicates of cut sections (10 cm and 40 cm) of different colored ERB (Theraband, Akron, OH) (tan, yellow, red, green, blue, and black, with colors arranged from least stiff most stiff) were placed in an Instron Biomechanical 5969 testing device. Bands were distracted at 3 m/min for 30 cycles at 1.25, 1.5 and 1.75 times the original length and maximal forces per cycle were recorded. Data were analyzed using ANOVA to evaluate different ERB. Mean forces were compared using Tukey’s test, and Pearson’s correlations were calculated in XLSTAT. Significance was set at P < 0.05.

**Results:** The mean forces produced at each elongation length are indicated in Table 1. There was a strong linear correlation of elongation length for each color of ERB (P < 0.05, Table 2). There were significantly lower values measured using the 40 cm versus the 10 cm band length for all colors (Table 3).

**Table 1:** Mean forces (N ± standard deviation) produced using different ERB lengths, various elongation values, and different therapeutic ERB

ERB color	Resting length (cm)	Elongation value compared to resting band length (Force values are N ± SD)		
Tan		1.25	1.5	1.75
	10	6.3 ± 0.3	10.3 ± 1.7	11.5 ± 0.2
	40	5.9 ± 0.1	8.9 ± 0.2	10.7 ± 0.2
Yellow		1.25	1.5	1.75
	10	10.7 ± 0.2	14.3 ± 0.2	15.8 ± 0.2
	40	9.6 ± 0.1	12.9 ± 0.1	14.9 ± 0.2
Red		1.25	1.5	1.75
	10	12.6 ± 0.3	16.6 ± 0.2	18.8 ± 0.3
	40	11.5 ± 0.2	15.6 ± 0.2	18 ± 0.3
Green		1.25	1.5	1.75
	10	16.9 ± 0.3	22.7 ± 0.3	25.7 ± 0.4
	40	15.5 ± 0.2	21.1 ± 0.3	24.3 ± 0.3
Blue		1.25	1.5	1.75
	10	19.3 ± 0.4	25.9 ± 0.4	29.1 ± 0.7
	40	17.7 ± 0.2	24.0 ± 0.3	27.6 ± 0.3
Black		1.25	1.5	1.75
	10	24.7 ± 0.2	33.4 ± 0.7	38.1 ± 0.6
	40	24.5 ± 0.5	33.4 ± 0.4	38.7 ± 0.5

**Table 2:** Calculated linear equations of elongation and each ERB color and resulting Pearson’s correlation coefficient

ERB color	Linear equation	R^2^ value
Tan	y = 10.0x + 3.9	0.95
Yellow	y = 10.7x + 7.8	0.96
Red	y = 12.7x + 9.1	0.97
Green	y = 17.5x + 12.3	0.97
Blue	y = 19.6x + 14.1	0.97
Black	y = 27.4x + 18.4	0.97

All colors resulted in strong correlation to a best fit line

**Table 3:** Mean forces (N ± standard deviation) generated by various elastic bands at 10 and 40 cm lengths with 95% confidence intervals

ERB color	Resting length (cm)	Force (N)	95% CI	P value
Tan	10	9.37 ± 0.04	[9.30–9.45]	< 0.0001
	40	8.49 ± 0.04	[8.42–8.57]	
Yellow	10	13.62 ± 0.01	[13.59–13.65]	< 0.0001
	40	12.43 ± 0.01	[12.41–12.46]	
Red	10	16.01 ± 0.01	[15.99–16.04]	< 0.0001
	40	15.02 ± 0.01	[14.99–15.04]	
Green	10	21.75 ± 0.01	[21.72–21.77]	< 0.0001
	40	20.32 ± 0.01	[20.29–20.34]	
Blue	10	24.74 ± 0.01	[24.71–24.77]	< 0.0001
	40	23.09 ± 0.01	[23.07–23.13]	
Black	10	32.23 ± 0.03	[32.18–32.28]	< 0.0001
	40	32.05 ± 0.03	[31.99–32.10]	

All color bands resulted in significantly different forces between the 10 and 40 cm resting lengths

**Conclusion:** Stiffer bands (darker colors), and increased elongation values generated greater forces. Shorter length ERB produced greater forces compared to a longer length of similar color. Further studies and calculations are underway to further characterize ERB based on these preliminary data. In addition, formulas may be generated to provide practitioners with information relative to the forces being applied during therapeutic exercises using ERB.

**References**

1. Ditmyer MM, Topp R, Pifer M. Prehabilitation in preparation for orthopaedic surgery. Orthop Nurs. 2002;21:43–54.
2. Christiansen DH, Falla D, Frost P, Frich LH, Svendsen SW. Physiotherapy after subacromial decompression surgery: development of a standardised exercise intervention. Physiotherapy. 2015;101:327–39.
3. Chen KM, Li CH, Huang HT, Cheng YY. Feasible modalities and long-term effects of elastic band exercises in nursing home older adults in wheelchairs: a cluster randomized controlled trial. Int J Nurs Stud. 2016;55:4–14.
4. Swank AM, Kachelman JB, Bibeau W, Quesada PM, Nyland J, Malkani A, et al. Prehabilitation before total knee arthroplasty increases strength and function in older adults with severe osteoarthritis. J Strength Cond Res. 2011;25:318–25.
5. In T, Jin Y, Jung K, Cho HY. Treadmill training with Thera-Band improves motor function, gait and balance in stroke patients. NeuroRehabilitation. 2017;40:109–14.
6. Hottinger HA, DeCamp CE, Olivier NB, Hauptman JG, Soutas-Little RW. Noninvasive kinematic analysis of the walk in healthy large-breed dogs. Am J Vet Res. 1996;57:381–8.

### A9 Variables affecting class IV laser absorbance

#### Daniel McCarthy^1^, Darryl Millis^1^, Agricola Odoi^2^

##### ^1^CARES Center, The University of Tennessee College of Veterinary Medicine, Knoxville, TN, USA; ^2^Biomedical and Diagnostic Sciences, The University of Tennessee College of Veterinary Medicine, Knoxville, TN, USA

###### **Correspondence:** Daniel McCarthy (dmccart6@utk.edu)

*Acta Veterinaria Scandinavica* 2019, **61**(**Suppl 1**):A9

**Background:** Laser therapy is increasingly used in veterinary medicine. Laser therapy, or photobiomodulation, has positive effects on wound healing, joint conditions, and analgesia for chronic and acute pain in people [1–4], but the effects rely on photon delivery to the appropriate tissues. Chromophores are substances that exist in tissues and reduce laser penetration. The type and thickness of tissues may also alter the penetration of photons through tissues. To our knowledge, no studies have evaluated these variables noninvasively in live dogs. The purpose of this study was to objectively evaluate class IV laser penetration through various tissues in dogs. We hypothesized that an increasing melanin and erythema index, and unclipped hair, would decrease photon penetration. Further, we hypothesized that laser penetration would vary among different tissue types.

**Materials and methods:** Twenty healthy dogs weighing between 15 and 30 kg were included in the study. Laser penetration was evaluated at six sites, including the pinna, triceps muscle, inguinal skin, caudal vertebra and caudal vertebra intervertebral space, distal common calcaneal tendon, and proximal caudal thigh. Tissue thickness, colorimeter measurement including melanin and erythema index, and laser penetration were evaluated prior to and after clipping hair at each designated site. Penetration was measured using a wavelength photodetector placed in a jig positioned 180 degrees from a class IV laser (CTC-12, LiteCure, Newark, DE) aperture at 0.5, 1, 3, and 5 W power settings. Mean laser penetration values were compared using generalized linear mixed models in SAS. Statistical significance was set at P ≤ 0.05

**Results:** No adverse effects were observed. Laser penetration decreased significantly with unclipped hair (94.6%) versus clipped hair (98.6%) and greater melanin index (each unit increased absorbance 0.033%, P < 0.0001) in all tissues. Erythema index did not influence laser penetration (P = 0.273). Tissue type also affected mean laser penetration (Table 1).

**Table 1:** Mean laser penetration through different tissues

Site	Penetration (%)
Pinna	7.02 A
Inguinal region	6.56 A
Calcaneal Tendon	5.12 A, B
Triceps muscle	2.59 B, C
Distal Intervertebral space	2.56 B, C
Caudal vertebra	0.00 C
Proximal thigh	0.00 C

Penetration through various tissues is not significantly different (P > 0.05) in tissues with the same letter. The greatest penetration was present through the pinna, while the lowest was in the caudal vertebra and proximal thigh

**Conclusion:** Unclipped hair decreased laser penetration by approximately 4%. Tissue type and thickness also affected laser penetration, with no penetration through triceps muscle and caudal vertebra. Based on the findings reported here, patients should be clipped prior to therapeutic laser application to improve transmission to tissues, and differences in tissue structure should be taken into account prior to laser application. Future studies are necessary to measure photon penetration to target tissues and to determine the dose of laser for ideal photobiomodulation of cells in live dogs.

**References**

1. Reddy GK, Stehno-Bittel L, Enwemeka CS. Laser photostimulation accelerates wound healing in diabetic rats. Wound Repair Regen. 2001;9:248–55.
2. Stelian J, Gil I, Habot B, Rosenthal M, Abramovici I, Kutok N, et al. Improvement of pain and disability in elderly patients with degenerative osteoarthritis of the knee treated with narrow-band light therapy. J Am Geriatr Soc. 1992;40:23–6.
3. Djavid GE, Mortazavi SMJ, Basirnia A, Roodsari GS, Jamili P, Sheikhbahaee N, et al. Low level laser therapy in musculoskeletal pain syndromes: pain relief and disability reduction. Lasers Surg Med. 2003;152:43.
4. Chow RT, Heller GZ, Barnsley L. The effect of 300 mW, 830 nm laser on chronic neck pain: a double-blind, randomized, placebo-controlled study. Pain. 2006;124:201–10.

### A10 Surface electromyography of the vastus lateralis, biceps femoris, and gluteus medius muscles in normal dogs during treadmill exercise with elastic resistance bands

#### Hannah McLean^1^, Darryl Millis^1^, David Levine^2^

##### ^1^Department of Small Animal Clinical Sciences, University of Tennessee College of Veterinary Medicine Knoxville, TN, USA; ^2^Department of Physical Therapy, University of Tennessee at Chattanooga, Chattanooga, TN, USA

###### **Correspondence:** Hannah McLean (hthurma2@vols.utk.edu)

*Acta Veterinaria Scandinavica* 2019, **61**(**Suppl 1**):A10

**Background:** Therapeutic exercises are an essential part of the rehabilitation of musculoskeletal and neurologic conditions, as well as strengthening and conditioning of dogs. Elastic resistance bands (ERB) are used to provide resistance during therapeutic exercise, thereby increasing muscle strength [1]. In humans, muscle activity using surface electromyography (EMG) is used in healthy populations to determine the role and interactions of different muscles during specific tasks as well as in clinical studies to assess muscle dysfunction or maladaptions due to neurologic or musculoskeletal injury or pain. Several studies have evaluated canine muscle activity using needle EMG or surface EMG during walking and trotting on a treadmill at different angles of inclination or declination [2–4]. The objective of this study was to evaluate surface EMG muscle activity of the vastus lateralis (VL), biceps femoris (BF), and gluteus medius (GM) muscles during walking and trotting on a treadmill with progressively increasing elastic band resistance in clinically normal dogs. We hypothesized that surface EMG muscle activity would increase with increasing resistance.

**Materials and methods:** Surface EMG (Noraxon, Scottsdale, AZ) was performed during treadmill walking and trotting with 4 different stiffnesses of ERB (Theraband, Akron, OH– Yellow, Red, Green, Blue) secured above the hock. Dogs were acclimated to the treadmill prior to the study. The treadmill velocity was set to a comfortable walk (1.4–2.0 MPH) and trot (3.0–4.1 MPH). The ERB were held at 120–150% elongation based on tensiometer measurements [5]. Raw EMG data was rectified, smoothed and filtered prior to statistical analysis. The means of maximal and mean muscle potentials during 3 gait cycles were compared among the different ERB. Data were analyzed using a mixed model analysis. Significance was set at the P < 0.05 level.

**Results:** At the walk and trot, the maximum amplitude of the GM was significantly lower than BF and VL for all ERB (P < 0.05). During the walk, the maximum amplitude increased with progressively increasing resistance of the different ERBs (P < 0.05). At the trot, the GM again had a significantly lower amplitude at all resistances, and the maximum amplitude of the VL and BF increased with increasing resistance of the different ERBs (P < 0.05).

**Conclusion:** Increased muscle activity in the BF and VL can be achieved by use of ERB and muscle activity progressively increases with increasing band resistance at the walk and trot. Specific knowledge of muscle activation patterns during therapeutic exercises should allow practitioners to target specific muscles for strengthening.

**References**

1. Taylor NF, Dodd KJ, Damiano DL. Progressive resistance exercise in physical therapy: a summary of systematic reviews. Phys Ther. 2005;85:1208–23.
2. Breitfuss, Franz M, Peham C, Bockstahler B. Surface electromyography of the vastus lateralis, biceps femoris, and gluteus medius muscle in sound dogs during walking and specific physiotherapeutic exercises. Vet Surg. 2015;5:588–95.
3. Bockstahler BB, Gesky R, Mueller M, Thalhammer JG, Peham C, Podbregar I. Correlation of surface electromyography of the vastus lateralis muscle in dogs at a walk with joint kinematics and ground reaction forces. Vet Surg. 2009;38:754–61.
4. Lauer SK, Hillman RB, Li L, Hosgood GL. Effects of treadmill inclination on electromyographic activity and hind limb kinematics in healthy hounds at a walk. Am J Vet Res. 2009;70:658–64.
5. Uchida, MC, Nishida, MM, Sampaio, RAC, Moritani T, Arai, H. Thera-band^®^ elastic band tension: reference values for physical activity. J Phys Ther Sci. 2016;28:1266–71.

### A11 The use of radial pressure wave therapy to manage chronic musculoskeletal pain in dogs

#### Britt Mills

##### Mills Veterinary Services, Armstrong, BC, Canada

###### **Correspondence:** Britt Mills (millsdvm@gmail.com)

*Acta Veterinaria Scandinavica* 2019, **61**(**Suppl 1**):A11

Radial pressure wave therapy has been used in humans to treat chronic soft tissue injuries, especially tendinopathies. Postulated mechanisms of action include transmission of positive pressure energy through tissue, cavitation, alteration of ion channels, stimulation of nitric oxide pathways, stimulation of angiogenesis, reduction in inflammation, and tissue and nerve regeneration. It is useful in small animal veterinary medicine because it is very well tolerated in unsedated animals, and it provides a viable method to treat pain when non-steroidal anti-inflammatories cannot be used. The purpose of this study is to demonstrate the efficacy of radial pressure wave therapy in treating various chronic pain conditions in dogs. Over a period of 6 months, ten dogs were selected for case studies. They all had pain conditions that were obvious to the owner and had persisted for more than 1 month. The location of pain varied but included shoulder and stifle pain, generalized hindquarter pain, and spine and forelimb pain. All dogs received three treatments approximately 2 weeks apart and efforts were made to ensure there were no changes in medication or exercise during the treatments. The treatment intensity and duration was tailored to the size of the dog and the site treated. The treatments were evaluated by owner reports of changes in demonstrated pain and lameness, and also by veterinary evaluation. No dog required sedation and all dogs except two showed a clear improvement in pain.

**Reference**

1. Moya D, Ramon S, Schaden W, Wang CJ, Guiloff L, Cheng JH. The role of extracorporeal shockwave treatment in musculoskeletal disorders. J Bone Joint Surg Am. 2018;100:251–63.

### A12 Effect of aquatic exercise on cardiovascular parameters of dogs

#### Darryl Millis, Rachel Dickson, Ellen Camp, Jose Guevara, Marti Drum

##### CARES Center, University of Tennessee College of Veterinary Medicine, Knoxville, TN, USA

###### **Correspondence:** Darryl Millis (boneplate@aol.com)

*Acta Veterinaria Scandinavica* 2019, **61**(**Suppl 1**):A12

**Background:** A previous study found that heart rate (HR) and respiratory rate (RR) increased prior to fatigue with ground treadmill exercise [1]. Little information exists regarding aquatic exercise and the cardiovascular system in pet dogs compared to people [2, 3]. Because cardiovascular parameters in pet dogs may differ from those of elite athletes, we collected data from pet dogs. Our main objective was to measure cardiovascular, respiratory, and thermal parameters of dogs undergoing a graded fitness test in an underwater treadmill with three different water levels, and during swimming. We hypothesized that HR and RR would increase during exercise for each condition, and these parameters would peak prior to fatigue.

**Methods and methods**: Fifteen adult dogs, weighing 20–40 kg and of various fitness levels were evaluated. Dogs were acclimated to an underwater treadmill (UWTM) and a swimming pool (SP) prior to data collection. Resting HR; systolic (SBP), diastolic (DBP) and mean blood pressure (MBP); rectal temperature; and RR were obtained. Dogs underwent a graded exercise test in the UWTM at 3 water levels performed in random order, (1) carpus (C), (2) midantebrachium (MA), (3) elbow (E), until mild fatigue. Dogs walked 2 km/h for 5 min, followed by a 1 min rest period to obtain data. Dogs were walked for additional 5 min intervals at increasing velocity (0.5 km/h for each period) until mild fatigue, with 1 min rest periods between sessions. Because velocity could not be controlled during swimming, data were collected every minute, with a 1 min rest period to collect data. Data were analyzed using ANOVA to evaluate changes over time and among groups.

**Results:** Dogs exercised for a mean of 56.3, 58.3, 53.3, and 6.2 min for C, MA, E, and SP, respectively. In general, SP HR was greatest, and C HR was lowest (Fig. 1). HR increased steadily throughout exercise with minimal indication of fatigue. Similarly, RR gradually increased throughout exercise in all groups, with the greatest RR within 15 min of fatigue in UWTM groups and within 3 min in the SP group (Fig. 2). There were no significant changes in SBP, DBP, MBP, or temperature to indicate fatigue (P > 0.05).

**Fig. 1:**
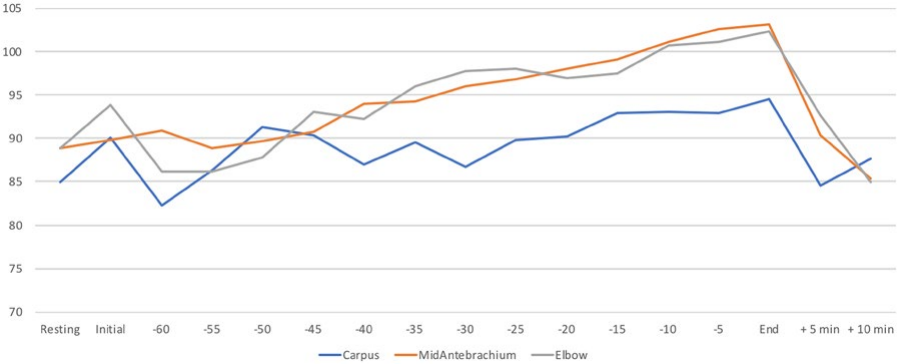
Heart rate in dogs exercising in an underwater treadmill at different water levels

**Fig. 2:**
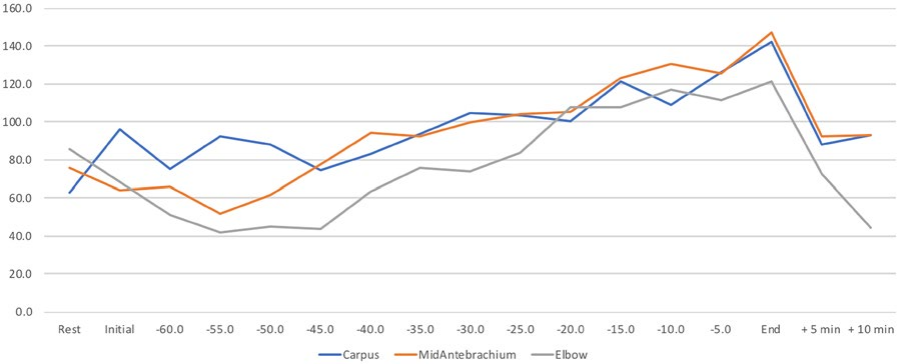
Respiratory rate in dogs exercising in an underwater treadmill at different water levels

**Conclusion:** Mild increases in HR and moderate increases in RR may be useful to design conditioning protocols to induce mild training stress. Possible recommendations from this graduated exercise test suggest an increase in 10 heartbeats per min, or a RR of 100 may precede exercise fatigue.

**References**

1. Camp E, Dickson R, Guevara J, Drum M, Millis D. Cardiovascular parameters of exercising pet dogs. Acta Vet Scand. 2016;58:A44.
2. Whitley JD, Schoene LL. Comparison of heart rate responses: water walking versus treadmill walking. Phys Ther. 1987;67:1501–4.
3. Johnson BL, Stromme SB, Adamczyk JW, Tennoe KO. Comparison of oxygen uptake and heart rate during exercises on land and in water. Phys Ther. 1977;57:273–8.

### A13 The effect of a dynamic warm-up protocol on performance in agility dogs

#### Romany Pinto, Emily Horan, Kira Penney, Sarah Parker, Cindy Shmon

##### Western College of Veterinary Medicine, Saskatoon, Saskatchewan, Canada

###### **Correspondence:** Romany Pinto **(**romany.pinto@usask.ca)

*Acta Veterinaria Scandinavica* 2019, **61**(**Suppl 1**):A13

**Background:** Dynamic warm-up can improve performance and prevent injuries in human athletes [1]. To our knowledge, no studies have investigated the effects of warm-up in dogs, despite significant injury rates in sports such as agility [2, 3, 4, 5]. We wished to assess the effects of a dynamic warm-up on performance in agility dogs.

**Materials and methods:** We recruited 22 agility dogs of varying age and breed. Owners performed their dog’s normal pre-agility preparation prior to completing an agility course. Electronic timing equipment provided times to 0.01 s. All runs were videotaped. Owners were randomly divided into a control group (CG) and a warm-up group (WG). The WG received instruction on the dynamic warm-up. The protocol was based on those used in several human studies. It included cardiovascular activity, dynamic stretches, passive stretches, proprioceptive activities and agility activities. Four weeks later, the CG handlers did their normal pre-agility routine. The WG did the dynamic warm-up protocol. The dogs ran the same course as on Day 1. The blinded principal investigator analyzed each video and determined the number of times off task (TOT) for each dog. This included any time a dog paused, went off course, or restarted the weave poles. Day 1 times were subtracted from Day 2 times to give an outcome of change in time for each dog. Day 1 TOT was subtracted from Day 2 TOT to give an outcome of change in TOT for each dog. The differences between groups were compared using the Wilcoxon Rank sum test.

**Results:** Twenty-one dogs completed the study protocol. Dogs in the CG were a median of 2.48 s slower on Day 2, while WG dogs were a median of 3.18 s faster (Table 1, Fig. 1). The difference between groups was significant (P = 0.035). The CG dogs had a median of no difference in TOT between Day 2 and Day 1. The WG dogs had a median of 1 fewer TOT on Day 2 (Fig. 2). The difference between groups was nearing significance (P = 0.067). Seven of the 8 faster dogs (88%) in the WG had fewer TOT on Day 2.

**Table 1:** List of medical conditions treated with radial pressure wave therapy and outcomes after three treatments 2 weeks apart

Condition	Treatment	Comments
Partial ACL tear	2000 shocks 3.0 bar	Return to soundness
Generalized hindquarter pain	4000 shocks 4.5 bar	Improvement in difficulty rising and mobility
Medial shoulder instability	3000 shocks 3.0 bar	Resolution of lameness, return to competition
Back pain	4000 shocks at 4.5 bar	Improvement in mobility and pain
Partial ACL tear	2000 shocks at 3.0 bar	No change in lameness
Hip dysplasia	4000 shocks at 5.0 bar	Improvement in mobility and pain
Shoulder pain	2000 shocks at 3.0 bar	Improvement in lameness
Elbow dysplasia	2000 shocks at 3.0 bar	No change in lameness
Generalized hindquarter pain	4000 shocks at 4.0 bar	Improvement in difficulty rising and mobility
Back pain	4000 shocks at 4.5 bar	Improvement in mobility and pain

**Fig. 1:**
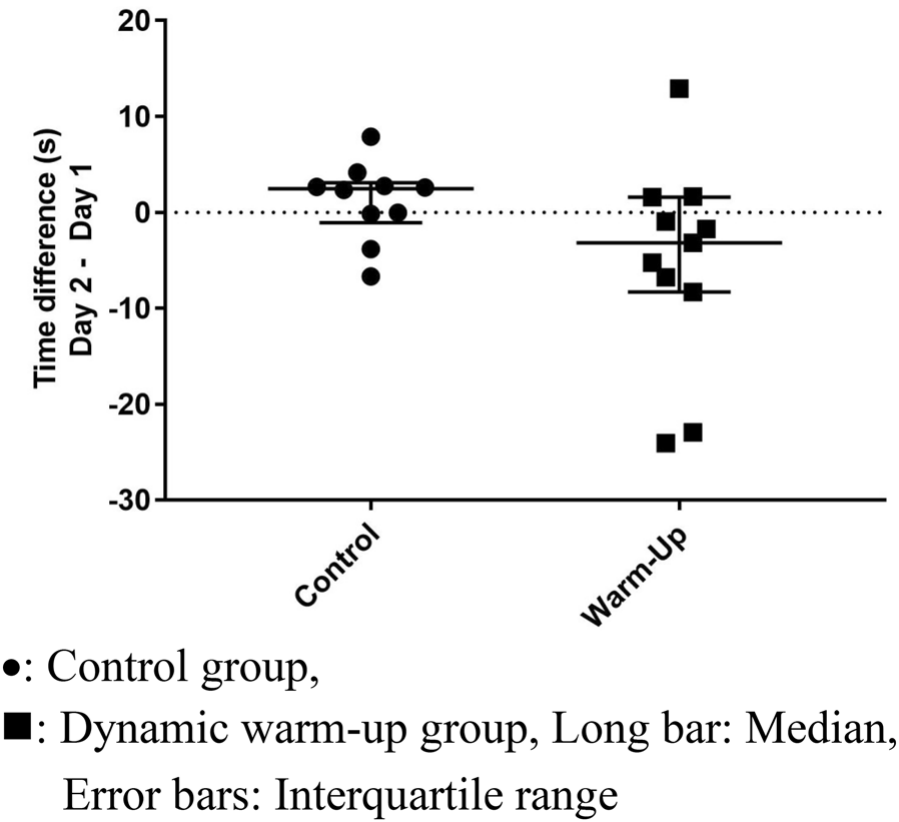
Difference in time taken to complete an agility course on two separate days (Day 2–Day 1) for individual dogs in either the warm-up group or control group

**Fig. 2:**
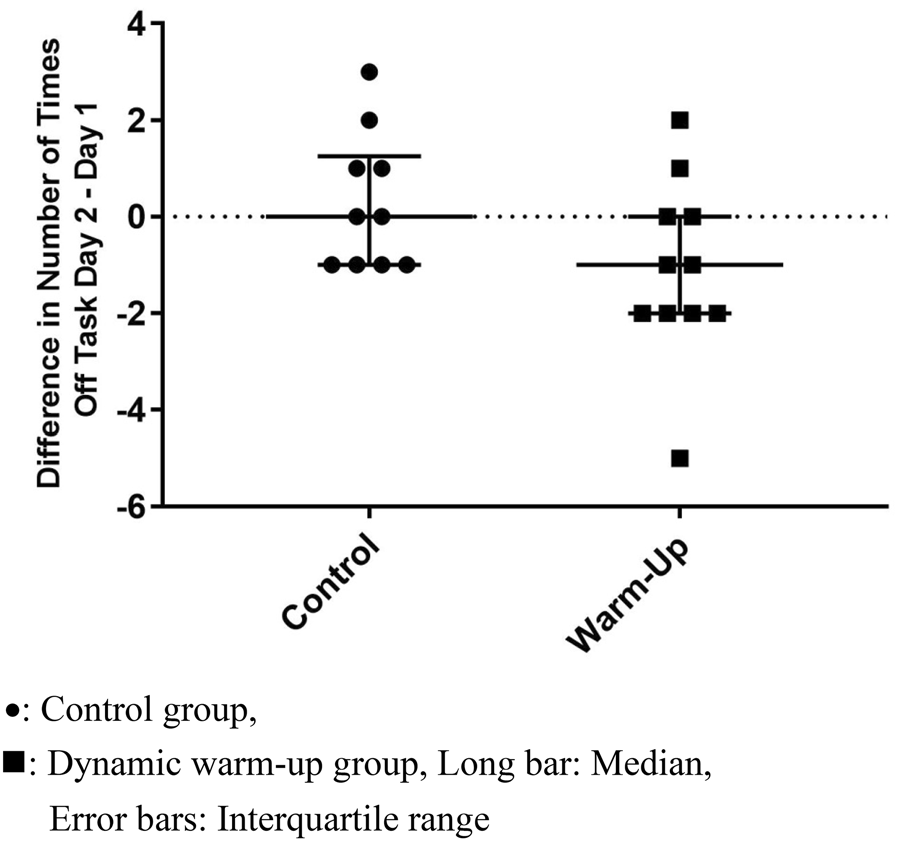
Difference in number of times off task on two separate days (Day 2–Day 1) for individual dogs in the control group and the warm-up group

**Conclusion:** The dynamic warm-up protocol significantly reduced agility run times. This may be due in part to improved focus and fewer TOT. Further research is needed to investigate the protocol’s effect on dogs’ running speed as well as any effects on injury rates.

**Acknowledgements:** The authors acknowledge Jason Good, Christina Tollett, Alexandra Wentzell, Christine Wilson and the Saskatchewan agility clubs for their assistance with this study.

**References**

1. Behm DG, Chaouachi A. A review of the acute effects of static and dynamic stretching on performance. Eur J Appl Physiol. 2011;111:2633–51.
2. Cullen KL, Dickey JP, Bent LR, Thomason JJ, Moëns NM. Survey-based analysis of risk factors for injury among dogs participating in agility training and competition events. J Am Vet Med Assoc. 2013;243:1019–24.
3. Cullen KL, Dickey JP, Bent LR, Thomason JJ, Moëns NM. Internet-based survey of the nature and perceived causes of injury to dogs participating in agility training and competition events. J Am Vet Med Assoc. 2013;243:1010–8.
4. Kerr ZY, Fields S, Comstock RD. Epidemiology of injury among handlers and dogs competing in the sport of agility. J Phys Act Health. 2014;11:1032–40.
5. Levy M, Hall C, Trentacosta N, Percival M. A preliminary retrospective survey of injuries occurring in dogs participating in canine agility. Vet Comp Orthop Traumatol. 2009;22:321–4.

### A14 Euthanasia diversion by use of extracorporeal shockwave therapy to improve mobility and decrease pain in a Treeing Walker hunting dog

#### Tamara Sue Shearer

##### Western Carolina Animal Pain Clinic, Sylva, NC, USA

###### **Correspondence:** Tamara Sue Shearer (tshearer5@frontier.com)

*Acta Veterinaria Scandinavica* 2019, **61**(**Suppl 1**):A14

**Background:** Extracorporeal shockwave therapy should be considered as part of the multimodal therapy to support palliative and hospice care patients to help restore function and mitigate pain. Patients may present for euthanasia, hospice, or palliative care when symptoms of a chronic condition interfere with activities of daily living, a decision is made not to pursue curative treatment, or when a patient has progressive consequences of a trauma which is associated with health complications [1].

**Materials and methods:** A retired 9-year old, 32 kg, neutered male Treeing Walking Coonhound presented to the Western Carolina Animal Pain Clinic for euthanasia because of a bilateral forelimb lameness associated with osteoarthritis of the humeroradial and humeroulnar joint which had exacerbated 2 weeks prior to the visit. Prior to the visit, his therapy included carprofen, gabapentin, amantadine, injectable glycosaminoglycan, oral supplements, laser therapy, acupuncture, therapeutic exercise and joint mobilizations [2, 3, 4, 5]. New modifications of existing dosages had failed to mitigate the pain.

**Results:** Upon presentation, the dog had a lameness score of 3/5 and pain score of 8/10. Quality of life scale was 275/500 [6]. Under light sedation (0.3 mg dexmedetomidine, IM), the patient received 3 treatments of 750 shocks with an energy level of 0.25 mJ/mm^2^ using the Storz Duolith^®^ Vet divided between the medial and lateral areas of the elbows every 2 weeks for 3 treatments. At the end of the series of treatments the lameness score improved to 1/5, quality of life to 450/500 and the pain scale decreased to 2/10.

**Conclusion:** Based on this case study, extracorporeal shockwave therapy should be offered as another treatment option over euthanasia for clients that have a desire to palliate a painful lameness.

**References**

1. Shearer T. Pet hospice and palliative care protocols. Vet Clin North Amer Small Anim Pract. 2011;41:507–18.
2. Shearer T, August K. Physical medicine, rehabilitation, complementary and integrative medicine treatment modalities. In: Shanan A, Pierce J, Shearer T, editors. Hospice and palliative care for companion animals. Hoboken NJ: Wiley Blackwell; 2018; 10.1002/9781119036722.ch19
3. Millis D, Levine D. Exercises for proprioception and balance. In: Millis D, Levine D, editors. Canine rehabilitation and physical therapy. 2nd ed. Philadelphia: Elsevier; 2014. p. 484–94
4. Durant A, Millis D. Applications of extracorporeal shockwave in small animal rehabilitation. In: Millis D, Levine D, editors. Canine rehabilitation and physical therapy. 2nd ed. Philadelphia: Elsevier; 2014. p. 381–9.
5. Hanks J, Levine D, Bockstahler B. Physical agent modalities in physical therapy and rehabilitation of small animals. Vet Clin North Am Small Anim Pract. 2015;45:33–7.
6. Xie H. 3 steps of palliative care and end-of-life care. In: Traditional Chinese Veterinary Medicine (TCVM) Palliative Care and End-of-Life Care Lecture series 2018.

### A15 Heart rate testing in agility dogs

#### Gillian Tabor^1^, Hannah Carmicheal^1^, Nicky Grant^2^

##### ^1^Animal Welfare Research and Knowledge Exchange Arena, University Centre Hartpury, Gloucester, UK; ^2^Win Clinic, Willowbrook Garden Centre, Wellington, UK

###### **Correspondence:** Gillian Tabor (Gillian.tabor@hartpury.ac.uk)

*Acta Veterinaria Scandinavica* 2019, **61**(**Suppl 1**):A15

**Background:** Heart rate (HR) has been used as a proxy measure for exercise intensity in human and horse training but its use is less reported in dog training. Agility dog trainers could use peak and mean heartrate of dogs undergoing agility trials to report episodes of physiological stress and fitness. Currently there are no studies published evaluating technique or outcome for HR testing of agility dogs over an agility run consisting of a timed course of obstacles. The aim of this study was to use a simple manual method of heartrate monitoring, pre-and post-agility trial, to test the hypothesis that heartrates would be higher after completion of the course.

**Materials and methods:** HR of 39 UK Kennel club rated grade 7 dogs (mean age 5.7 ± 1.7 years, 11 males, 18 females, variable breeds) were collected immediately preceding (PreHR) and after (PostHR) competing over an agility course. The course consisted of 168 m with 20 obstacles (13 jumps, 3 tunnels, set of 12 weaves, dogwalk, A-frame, see-saw) and was part of a 1-day camp for agility performance. Manual palpation was used to locate the femoral artery in the medial thigh region and dogs’ pulses were counted for 30 s. The number of beats per minute (BPM) was derived by multiplying by this number by two, to obtain a HR.

**Results:** Data from 22 dogs were excluded due to unreliability of HR collection as a result of the behaviour of the dog. HR of 17 dogs were successful collected at the two measurement points (PreHR mean 95.5 ± 19.7 BPM; PostHR mean 97.6 ± 14.4 BPM). The was no significant differences between PreHR and PostHR (t_16_ = 0.388 P = 0.703).

**Conclusion:** The behavioural excitement and rapid movement of the dogs in the competition environment limited the ability to collect HR data in this group of dogs. Of those that were collected there was a large spread of HR BPM with no mean difference between PreHR and PostHR. Physiological arousal due to excitement in this scenario appeared to be the limiting factor in the reliability of using the HR to measure exercise intensity. Therefore to record training load data an alternative method would need to be tested, such as continuous monitoring and data capture during the agility trial using technology to log HR.

## Equine

### A16 Effects of chiropractic on static and dynamic muscle parameters in 6 sport horses

#### Elizabeth Verity Acutt^1^, Sarah Sonia le Jeune^2^, Bruno Henri Pypendop^2^

##### ^1^W.R. Pritchard Veterinary Medical Teaching Hospital, Department of Surgical and Radiological Sciences, University of California, Davis, CA, USA; ^2^Department of Surgical and Radiological Sciences, University of California, Davis, CA, USA

###### **Correspondence:** Elizabeth Verity Acutt (evacutt@ucdavis.edu)

*Acta Veterinaria Scandinavica* 2019, **61**(**Suppl 1**):A16

**Background:** Chiropractic has become widely used in equine practice, however little objective data exists supporting the efficacy of this modality in horses [1, 2]. Non-invasive methods of assessment of muscle function have been validated in horses [3, 4]. Bioimpedance analysis (BI) uses measurements of current through cell membranes and tissue interfaces to infer information regarding the degree of contraction and health of the selected muscle [3]. Acoustic myography (AMG) analyzes the low frequency sounds created during muscular activity and uses them to determine the way in which the central nervous system recruits and uses the active fibers in a muscle (efficiency, E), the number of active muscle fibers (spatial summation; S) and the frequency with which they contract (temporal summation, T) [5]. The objective of this study was to assess the effect of chiropractic treatment on static bioimpedance (BI) and dynamic acoustic myography (AMG) of paired muscle groups in healthy sport horses. We hypothesized that chiropractic would affect BI and AMG variables.

**Materials and methods:** Recordings were taken before and at 24, 48 and 72 h after chiropractic treatment of six healthy, client-owned horses by a veterinarian certified in veterinary chiropractic. BI of trapezius, latissimus dorsi, longissimus dorsi and gluteus medius muscles were recorded at rest; AMG data were collected from paired gluteus medius, longissimus dorsi and latissimus dorsi muscles at walk and trot in a straight line. Data were screened for significance (P = 0.05) using RMANOVA and Dunnett’s test. Gait abnormalities were assessed subjectively and objectively (Lameness Locator™) and sensitivity at superficial acupuncture points was recorded at all time points.

**Results:** All horses tolerated the procedures well. Sensitivity at acupuncture points was present initially in 5/6 horses and was abolished immediately after chiropractic in all five. Signs of mild lameness were detected objectively in all horses prior to chiropractic and throughout the course of the study. BI: Bioimpedance scores for the left trapezius muscle were significantly altered at 24 and 72 h. AMG: The balance score of the gluteus medius muscle at walk was significantly affected by time, as was its efficiency at the trot.

**Conclusion:** This small pilot study reveals that objective measurements of muscle function improve following chiropractic, lasting for at least 72 h post-treatment. This warrants further investigations in a larger number of subjects and in additional muscle groups.

**Acknowledgements:** Adrian Harrison D.Phil. for assisting in data collection and processing.

**Study compliance:** The study complied with the Animal Care and Use Committee of the University of California, Davis.

**References**

1. Haussler K. The role of manual therapies in equine pain management. Vet Clin North Am Equine Pract. 2010;26:579–601.
2. Haussler K. Joint mobilization and manipulation for the equine athlete. Vet Clin North Am Equine Pract. 2016;32:87–101.
3. Harrison A, Elbrond V, Riis-Olesen K, Bartels E. Multi-frequency bioimpedance in equine muscle assessment. Physiol Meas. 2015;36:453–64.
4. Riis K, Harrison A, Riis-Olesen K. Non-invasive assessment of equine muscular function: a case study. Open Vet J. 2013;3:80–4.
5. Harrison A, Danneskiold-Samsøe B, Bartels E. Portable acoustic myography—a realistic noninvasive method for assessment of muscle activity and coordination in human subjects in most home and sports settings. Physiol Rep. 2013;1:e00029.

### A17 Facial expressions are not able to detect pain in horses with localized inflammatory process

#### Júlia Ribeiro Garcia Carvalho^1^, Pedro Henrique Esteves Trindade^2^, Gabriel Conde^1^, Marina Lansarini Antonioli^3^, Paula Patrocínio Dias^4^, Marcelo Aparecido Chinelatto^4^, Paulo Aléscio Canola^3^, Guilherme Camargo Ferraz^1^

##### ^1^Department of Animal Morphology and Physiology, Faculty of Agrarian and Veterinary Sciences, São Paulo State University (UNESP), Jaboticabal, SP, Brazil; ^2^Department of Veterinary Anesthesiology and Surgery, Faculty of Medicine Veterinarian and Animal Science, São Paulo State University (UNESP), Botucatu, SP, Brazil; ^3^Department of Veterinary Clinic and Surgery, Faculty of Agrarian and Veterinary Sciences, São Paulo State University (UNESP), Jaboticabal, SP, Brazil; ^4^Department of Materials Engineering, School of Engineering of São Carlos, University of São Paulo (USP), São Carlos, SP, Brazil

###### **Correspondence:** Júlia Ribeiro Garcia Carvalho (juliargc@hotmail.com)

*Acta Veterinaria Scandinavica* 2019, **61**(**Suppl 1**):A17

**Background:** Horse welfare has been scientifically and non-invasively evaluated based on horses’ body language [1–3]. Among behavioral indicators, facial expressions have been considered reliable signs of horses’ internal state [2]. Facial features are currently used to assess pain on horses post-surgeries and induced pain, but not specifically with local inflammation [4, 5]. The objective of this study is to investigate if facial expressions are able to detect pain originated from local inflammation in horses submitted to implantation of biopolymers.

**Materials and methods:** Six horses were submitted to surgical implantation of a biopolymer blend PLA/PCL, which was performed in the neck board subcutaneous space. The horses were sedated with detomidine (0.01 mg/kg), and an anesthetic button was made with lidocaine (2%). Horses’ faces were recorded with a video camera 1 h before, 24 and 48 h after the procedure, for 5 min. Two 30-s video clips of the right profile of the head/face of the horse were extracted from the videos [4]. The videos were given three scores levels (0, 1 and 2) with the high score representing the maximum pain expression for each facial feature evaluated (ears position, orbital tightening, inner brow raiser, eye tightness, nostril dilated, tension of the muzzle and tonus of the masticatories muscles; Fig. 1).

**Fig. 1:**
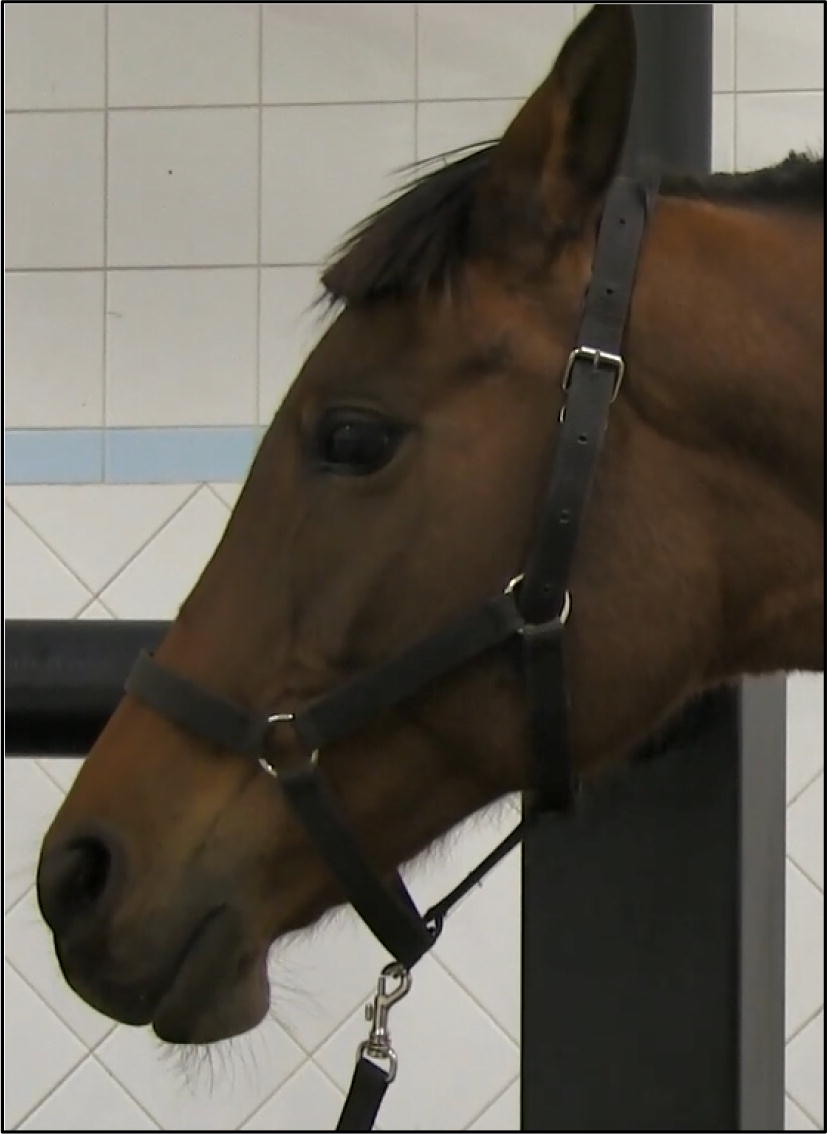
Still image from 30 s video clips of one horse 24 h after (procedure) implantation of a biopolymer in the subcutaneous space of the region of the neck board. The image show horse orienting their ears backward, inner brow raised, nostril dilated, tension of the muzzle and tone of the masticatories muscles

Cutaneous hyperalgesia was evaluated by von Frey monofilaments (VFM), which were applied in four different points around the implantation site. The mechanical threshold is determined by the diameter of the filament at which a behavioural response is obtained. This value was converted to kg/force [6]. If there was no aversive response with the smaller diameter (0.432 mm), the larger filament was applied until the animals demonstrated an aversive response or the larger diameter filament was used (1.143 mm).

We performed three Principal Components Analyses (PCA), one for each sampling time (1 h before, 24 and 48 h after procedure) using all face scores. For each PCA we extracted the scores of each animal in representative dimensions (eigenvalues ≥ 1). The score that the PCA algorithm provides is a vector that concentrates the values of all variables included in the PCA referring to the same dimension. Then, the extracted face scores (EFS) and VFM data from each sampling time were compared using the Friedman test (P < 0.05). Relation between EFS and VFM was estimated with Pearson correlation.

**Results:** Nociceptive threshold 1 h before the procedure was lower than 24 and 48 h after the procedure (P = 0.002; Fig. 2). There were no differences between the sampling time for EFS (P = 0.562; Fig. 3). EFS and VFM did not correlate (r = 0.42; P = 0.086).

**Fig. 2:**
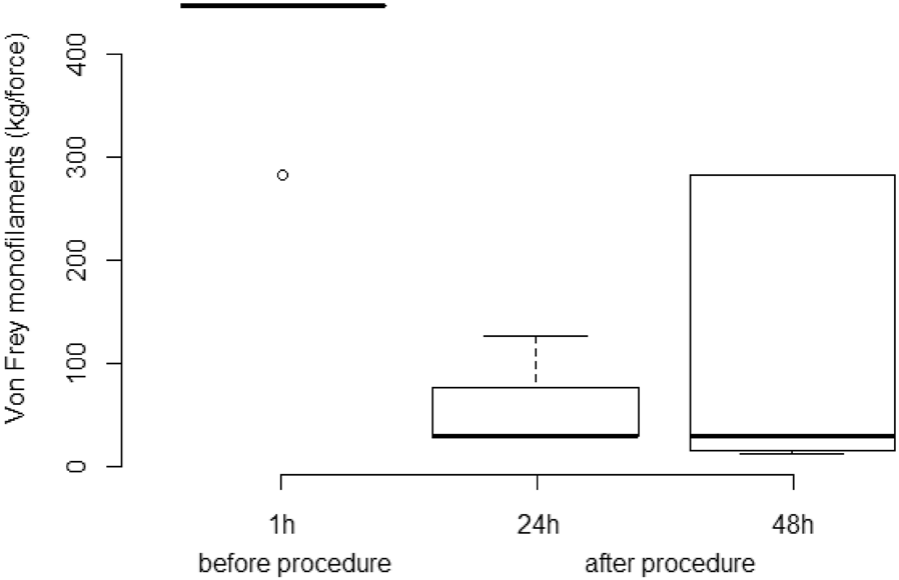
Boxplot of the von Frey monofilaments for six horses assessed 1 h before, 24 and 48 h after (procedure) implantation of a biopolymer in the subcutaneous space of the region of the neck board. Sensibility threshold was established for the diameter of the filament (0.432 to 1.143 mm), and this value was converted to kg/force by a conversion scale available from the manufacturer

**Fig. 3:**
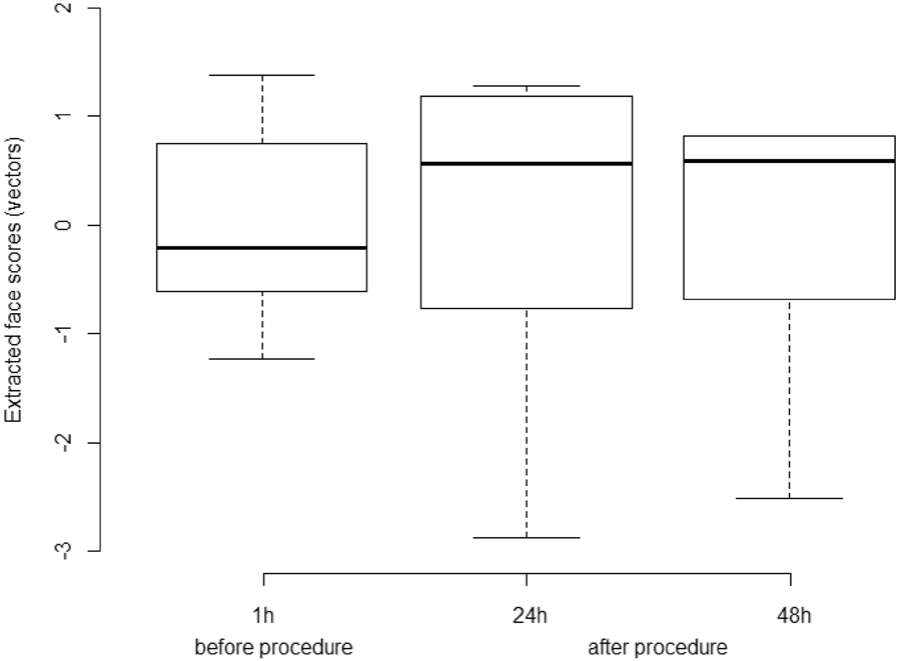
Boxplot of the extracted face scores for six horses assessed 1 h before, 24 h and 48 h after (procedure) implantation of a biopolymer in the subcutaneous space of the region of the neck board. The extracted face scores represented a vector from Principal Component Analysis (PCA) that concentrates the values of all face variables (ears position, orbital tightening, inner brow raiser, eye tightness, nostril dilated, tension of the muzzle and tonus of the masticatories muscles) included in the PCA referring to the same dimension

**Conclusion:** Facial expressions were not able to detect pain in localized inflammatory processes in horses.

**Declaration:** The experimental protocol was approved by the Ethics Committee of the São Paulo State University (nº 006548/17) and followed the standards care of Use of Animals.

**Acknowledgements:** São Paulo Research Foundation-FAPESP (Process nº 2017/10959-4).

**References**

1. Broom DM. Animal welfare: concepts and measurement. J Anim Sci. 1991;69:4167–75.
2. Descovich K, Wathan JW, Leach MC, Buchanan-Smith HM, Flecknell P, Farningham D, et al. Facial expression: an under-utilised tool for the assessment of welfare in mammals. ALTEX. 2017;34:409–29.
3. Grauw JC, Loon JP. Systematic pain assessment in horses. Veterinary J. 2016;209:14–22.
4. Gleerup KB, Forkman B, Lindegaard C, Andersen PH. An equine pain face. Vet Anaesth Analg. 2015;42:103–14.
5. Dalla Costa E, Minero M, Lebelt D, Stucke D, Canali E, Leach MC. Development of the Horse Grimace Scale (HGS) as a pain assessment tool in horses undergoing routine castration. PLoS one. 2014;9:e92281.
6. Touch Test™ Sensory Evaluators Operation Manual. Stoelting Co. https://www.stoeltingco.com/media/wysiwyg/58011_Touch_Test_Evaluator.pdf.

### A18 Effect of chiropractic manipulation on the active range of motion of the dorso-ventral flexion at the thoraco-lumbar back in the standing horse

#### Juan Carlos Garcia-de-Brigard^1^, Maria Fernanda Pardo-Valderrama^1^, Luis Carlos Mena-Aguilera^2^, Luna Gutierrez-Cepeda^3^

##### ^1^Hippo-Training EU, Bogotá, Colombia; ^2^Escuela de Equitacion, Ejercito de Colombia, Bogotá, Colombia; ^3^Universidad Complutense de Madrid, Madrid, Spain

###### **Correspondence:** Juan Carlos Garcia-de-Brigard (garciadebrigard@gmail.com)

*Acta Veterinaria Scandinavica* 2019, **61**(**Suppl 1**):A18

**Background:** Chiropractic manipulation and other high velocity-low amplitude thrusts on the thoraco-lumbar spine have long had anecdotic clinical reports of increasing the Range of Motion of the horse. Passive intervertebral ROM is increased after manipulation [1].

The aim of this study was to assess if, performing a full chiropractic adjustment of the thoracic, lumbar and sacral sections of the spine would increase the active Range of Motion (ROM_A_) of the Dorso-Ventral Flexion (DVF) at the Thoraco-Lumbar Back (TLB) on standing horses.

**Materials and methods:** ROM_A_ of the DVF at the TLB was evaluated in 21 clinically sound, actively ridden horses. For the purpose of this study, ROM_A_ was defined as the change in height of the back at (T15, T18, L2), induced with sudden pressure stimulation over the semitendinous muscle, when compared to the resting height. Measures were recorded three times per horse with a background measuring wall and high speed video. After this assessment, a chiropractic adjustment was performed on every segment that required it, over the thoracic, lumbar and sacral sections of the spine on 14 horses selected at random (Treatment group). The remaining 7 horses acted as control. A second ROM_A_ assessment was then performed on every horse, 20 min after the first one, recording the change in ROM_A_ between assessments. The technicians who performed the assessments and the treatments were independent and blinded to each other’s procedures.

**Results:** The change from first to second ROM_A_ assessments was significantly greater (P ˂ 0.05) for the Treatment Group (9.21 ± 2.99 cm vs. 10.36 ± 2.02 cm) than for the Control Group (8.86 ± 2.47 cm vs. 8.86 ± 2.67 cm).

**Conclusion:** The chiropractic adjustment of the thoracic, lumbar, and sacral spinal sections helped increase the ROM_A_ for DVF at the TLB, which could show a beneficial effect of chiropractic care on ROM_A_ of the back. Further studies are recommended to evaluate the effect of chiropractic manipulations on the ROM_A_ of the TLB for Dorsoventral extension, axial rotation and lateral bend, on both the standing and moving horse.

**Acknowledgements:** The authors thank the Equitation School of the Colombian Army and its commander, LtCol. David Andrés Rodríguez Camacho for permitting the use of its facilities and providing the horses involved in this study.

**Reference**

1. Haussler K, Martin C, Hill A. Efficacy of spinal manipulation and mobilisation on trunk flexibility and stiffness in horses: a randomised clinical trial. Equine Vet J Suppl. 2010;38:695–702.

### A19 Comparison of the effectiveness of Photobiomodulation (PBM) on Delayed Onset Muscle Soreness (DOMS) between Class IIIb and IV Laser and at high and low doses

#### Lauren Hunt, Robin L Gill

##### Writtle University College, Writtle, Chelmsford, UK

*Acta Veterinaria Scandinavica* 2019, **61**(**Suppl 1**):A19

**Background:** PBM is an expanding field and recent human studies focus on its effects to reduce DOMS [1]. To the author’s knowledge, no such study has been carried out in animals, specifically the equine species. This investigation aimed to compare two doses from both a class IIIb and class IV laser to determine if PBM had any effect on equine DOMS.

**Materials and methods:** A randomised crossover Latin square design was used with five horses. Pressure algometry tested nociception of 5 points on both sides of the horse located across the *biceps brachii, longissimus dorsi* and *gluteus medius* muscles. A baseline reading was taken prior to an underwater treadmill session as a controlled exercise protocol (CEP), 20 min with water to the level of the lateral styloid process. Treatment groups received laser intervention before CEP whilst the control group received sham laser. A further 4 days of data collection followed the exercise day with recordings taken at the same time. A 2-week washout period was implemented between conditions. Laser parameters are detailed in Table 1.

**Table 1:** Run times, times off task and differences between days (Day 2–Day 1) for dogs in the control and dynamic warm-up groups

Dog #	Day 1 time (s) [TOT]	Day 2 time (s) [TOT]	Difference (Day 2–Day 1) time (s) [TOT]
*Control group*			
3	50.23 [2]	43.56 [1]	− 6.67 [− 1]
2	49.57 [2]	45.76 [1]	− 3.81 [− 1]
6	41.29 [0]	41.14 [0]	− 0.15 [0]
9	60.06 [2]	60.03 [4]	− 0.03 [2]
4	80.91 [1]	83.26 [0]	2.35 [− 1]
1	53.79 [0]	56.39 [1]	2.6 [1]
5	44.87 [1]	47.54 [2]	2.67 [1]
10	65.27 [0]	68.03 [0]	2.76 [0]
7	91.28 [4]	95.43 [3]	4.15 [− 1]
8	51.18 [0]	59.08 [3]	7.90 [3]
Median	52.49 [1]	57.74 [1]	2.48 [0]
*Warm-up group*			
16	105.63 [7]	81.58 [5]	− 24.05 [− 2]
19	69.60 [5]	46.67 [0]	− 22.93 [− 5]
15	89.59 [6]	81.30 [4]	− 8.29 [− 2]
11	47.94 [2]	41.18 [0]	− 6.76 [− 2]
17	71.20 [3]	65.97 [1]	− 5.23[− 2]
20	47.64 [1]	44.46 [0]	− 3.18[− 1]
14	40.85 [0]	39.12 [0]	− 1.73 [0]
13	81.01 [5]	80.07 [4]	− 0.94 [− 1]
18	53.4 [1]	54.98[2]	1.58 [1]
21	64.33 [1]	66.00 [1]	1.67[0]
12	63.84 [2]	76.74 [4]	12.9 [2]
Median	64.33 [2]	65.97 [1]	− 3.18 [− 1]

A two-way repeated measures ANOVA was used to test significance of treatments, with studentized residuals to check for outliers.

**Results:** Points 1, 2 and 3 (*gluteus medius*) showed a significant 2-way interaction between time and treatment, F(16,464) 2.506, (P < 0.005). At 24 h post exercise, all doses improved compared to control, however, only CL4_L (P < 0.005) and CL4_H (P < 0.05) doses showed a positive significant difference. Furthermore, at 48 h post exercise only the CL4_H remained positively significant (P < 0.05) compared to the control.

**Conclusion:** Overall PBM has shown possible inhibition of equine DOMS in the gluteal region, with both class IV doses showing significance. There is speculation from this study that different doses and devices may be required in different muscle areas in order to provide optimum results. This follows patterns in human studies [1]. There were limitations to this study as horse activity varied, as did coat color (all, however, had dark skin) with some being clipped. With no previous research of the effects of PBM on equine DOMS, dosages were calculated and speculated from human research, possibly affecting their efficacy. PBM can be a promising treatment to inhibit equine DOMS, but more studies on its action and determining dosage parameters are required.

**Acknowledgements:** John Rushby at Celtic SMR ltd & Omega Laser Systems.

**Reference**

1. Oliveira AR, Vanin AA, Tomazoni SS, Miranda EF, Albuquerque-Pontes GM, De Marchi T, et al. Pre-exercise infrared photobiomodulation therapy (810 nm) in skeletal muscle performance and post exercise recovery in humans: what is the optimal power output?. Photomed Laser Surg. 2017;35:595–603.

### A20 Effects of the rider on dynamic muscle parameters of the longissimus dorsi muscle in 6 sport horses at the walk

#### Sarah S le Jeune^1^, Elizabeth V Acutt^2^, Bruno H Pypendop^1^

##### ^1^Department of Surgical and Radiological Sciences, University of California, Davis, CA, USA; ^2^W.R. Pritchard Veterinary Medical Teaching Hospital, University of California, Davis, CA, USA

###### **Correspondance:** Sarah S le Jeune (sslejeune@ucdavis.edu)

*Acta Veterinaria Scandinavica* 2019, **61**(**Suppl 1**):A20

**Background:** There is significant scientific support for the importance of the rider’s interactions with the back musculature of the horse [1–4]. However, objective effects of the rider on the muscle tissue have not been reported to date. Acoustic myography (AMG) is a non-invasive measurement of the low frequency sounds created during muscular activity which has been validated in horses and can generate information regarding the way in which the central nervous system recruits and uses the active fibers in a muscle (efficiency, E), the number of active muscle fibers (spatial summation; S) and the frequency with which they contract (temporal summation, T) [5, 6]. These measurements can then be added to give a total ‘ESTi’ score for each muscle. The objective of this prospective controlled experimental study was to assess the effects of the rider on the longissimus dorsi muscle fibers in the saddle support area in healthy sport horses at the walk. We hypothesized that the rider would increase acoustic myographic variables of the longissimus muscle under the saddle.

**Materials and methods:** Six healthy, client-owned sports horses were instrumented with a commercial acoustic myographic system (CURO^®^ MyoDynamik ApS, Denmark). Sensors were placed on the longissimus dorsi in the saddle support area at the level of the 15th thoracic vertebra. The horse’s own saddle and a standard saddle pad were routinely secured with a girth and AMG recordings were taken before and during ridden exercise at the walk in a straight line with an approximately 65 kg experienced rider. Data was screened for significance (P = 0.05) using a paired t-test.

**Results:** All horses tolerated the procedures well. The rider induced significant and immediate increases in E, T and ESTI scores of the longissimus muscle under the saddle bilaterally (Table 1).

**Table 1:** LASER parameters and characteristics

LASER device	Treatment	Wavelength (nm)	Aperture (cm^2^)	Power (W)	Diodes	Pulse rate (Hz)	Seconds (s)	Total Joules (J)
Class IIIb	Low dose (CL3_L)	820	0.125	1	5	1000	25	8
	High dose (CL3_H)						75	24
Class IV	Low dose (CL4_L)	808 ± 5	3	1.2	1	1000	17	8
	High dose (CL4_H)	905 ± 7					50	24

**Conclusion:** This study reveals that the rider induces significant changes in objective measurements of muscle function of the longissimus muscle of the horse under the saddle. Particularly the muscle becomes more efficient, with a higher frequency of muscle fiber activation in response to the rider’s weight. This has significant implications on the training and rehabilitation of horses under saddle and warrants further investigations in a larger number of subjects and in additional muscle groups.

**Acknowledgements:** Adrian Harrison for assisting in data collection and processing.

**Trial registration**: The study complied with the Animal Care and Use Committee of the University of California, Davis.

**References**

1. Greve L, Dyson S. The horse–saddle–rider interaction. Vet J. 2013;195: 275–81.
2. Peham C, Licka T, Schobesberger H, Meschan E. Influence of the rider on the variability of the equine gait. Hum Mov Sci. 2004;23: 663–71.
3. Greve L, Dyson S. Saddle fit and management: an investigation of the association with equine thoracolumbar asymmetries, horse and rider health. Equine Vet J. 2015;47: 415–21.
4. Greve L, Murray R, Dyson S. Subjective analysis of exercise-induced changes in back dimensions of the horse: the influence of saddle-fit, rider skill and work quality. Vet J. 2015;206:39–46.
5. Riis KH, Harrison AP, Riis-Olesen K. Non-invasive assessment of equine muscular function: a case study. Open Vet J. 2013;3:80–4.
6. Harrison, AP, Danneskiold-Samsøe B, Bartels EM. Portable acoustic myography—a realistic noninvasive method for assessment of muscle activity and coordination in human subjects in most home and sports settings. Physiol Rep. 2013;1:e00029.

### A21 Reestablishment of normal skin temperature in the cold limbs of a horse after craniosacral therapy

#### Solange Mikail^1^, Marina Villaça Issa de Araujo^1^, Celina Cutrale Tarantino^2^

##### ^1^Espaço Equus, Cotia, SP, Brazil; ^2^Horse Therapeutic Solutions, Aspelare, Belgium

###### **Correspondence:** Solange Mikail (mikail@termovet.com.br)

*Acta Veterinaria Scandinavica* 2019, **61**(**Suppl 1**):A21

**Background:** Craniosacral therapy is a form of manual therapy using gentle palpation to release fascial restrictions between the cranium and the sacrum. It is described in the literature that craniosacral therapy may influence the autonomous nervous system, but the underlying mechanism is not known. Thermography is a non-invasive diagnostic technique based on the caption of the infrared radiation emitted from the body and transforming it in a map of superficial body temperature. The temperature of the skin has a direct relationship with its blood flow, which is under the influence of the autonomous nervous system. An increased sympathetic tone can lead to cold extremities due to peripheral vasoconstriction (Fig. 1).

**Fig. 1:**
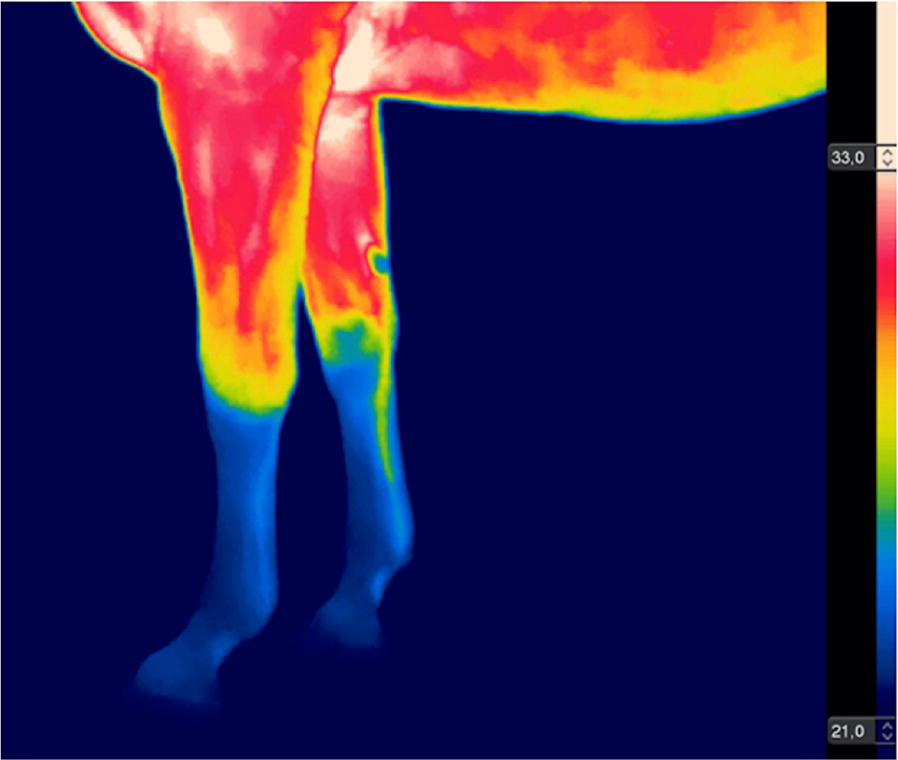
Thermography images of the cold limbs

This case study assessed the effect of craniosacral therapy on the cold limbs of a horse by means of thermographic evaluation.

**Materials and methods:** A 9-year-old, Brasileiro de Hipismo mare with a decrease temperature in both front limbs was evaluated. Craniosacral therapy was performed for 60 min (Fig. 2). Thermographic evaluation using a Thermographic Camera FLIR T530, was performed before, every 10 mins during the treatment, and 30 min after the treatment, i.e., 90 min from the first evaluation.

**Fig. 2:**
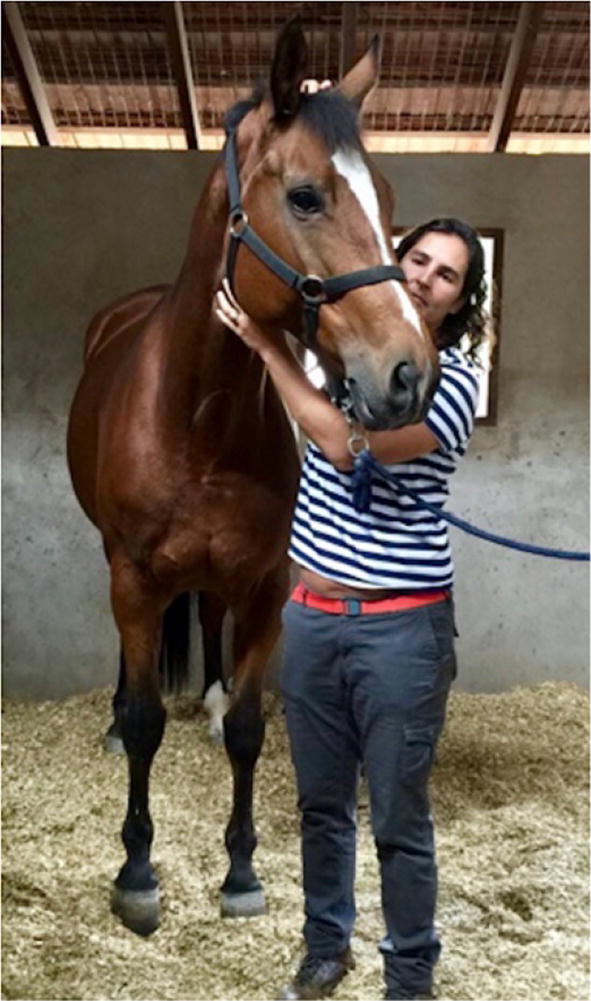
Craniosacral therapy

**Results:** Before the craniosacral therapy session, the mare had a marked decrease in skin temperature in both front limbs (mean 71.78 °F or 22.16 °C). After the session started, the evaluations showed that the temperature of the limbs began to increase in a proximal to distal pattern, and continued to increase reaching a normal temperature (mean 87.26 °F or 30.71 °C) 30 min after the session the end of the session, i.e., 90 min from the first evaluation. There was an increase in temperature of an average of 15.48 °F or 8.6 °C between the first and the last evaluation.

**Conclusion:** Craniosacral therapy could be used to increase superficial skin temperature by improving peripheral circulation in the limbs.

**Consent for publication**: The authors confirm that they have written informed consent to publish from the person in Fig. 3.

**Fig. 3:**
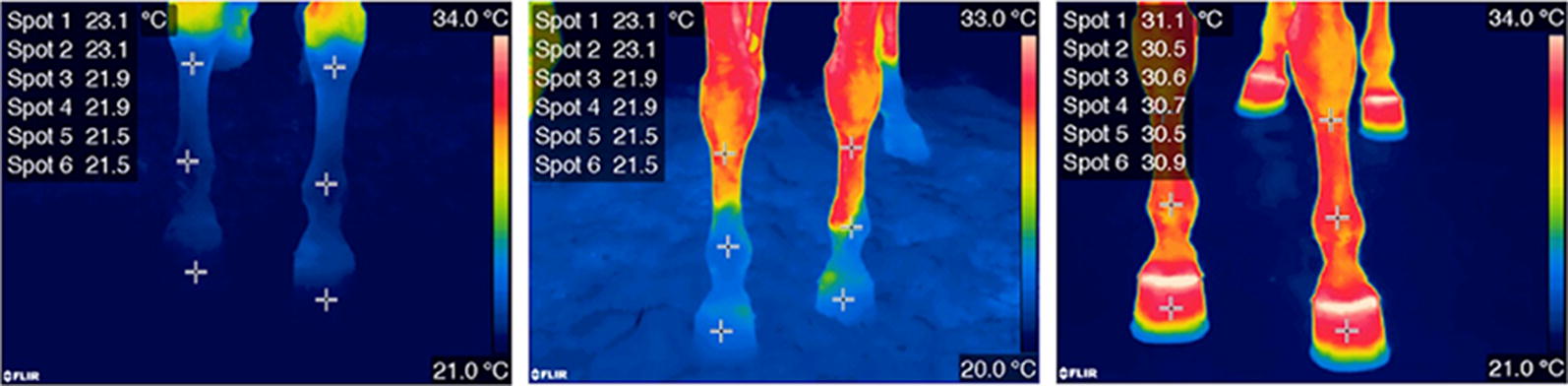
Sequence of thermographic examinations at 0, 30 and 90 min

**References**

1. Ernst E. Craniosacral therapy: a systematic review of the clinical evidence. Focus Altern Complement Ther. 2012;17:197–201.
2. Purohit RC, Pascoe D, De Franco B, Schumacher J. Thermographic evaluation of the neurovascular system in the equine. Thermol Int. 2004;14:89–92.

### A22 Correction of angular limb deformities in foals using kinesiology taping

#### Solange Mikail^1^, Marina Villaça Issa de Araujo^1^, Maria Vitória Jatobá^2^, Maria Isabel Gonçalves e Silva^2^, Luciana Menezes de Carvalho^3^

##### ^1^Espaço Equus, Cotia, SP, Brazil; ^2^Move Therapy Performance e Reabilitação Equina, Montes Claros, MG, Brazil; ^3^Clínica e Cirurgia de Grandes Animais, UFMT, Cuiabá, MT, Brazil

###### **Correspondence:** Solange Mikail (mikail@termovet.com.br)

*Acta Veterinaria Scandinavica* 2019, **61**(**Suppl 1**):A22

**Background:** Kinesiology Taping is a technique consisting of the use of elastic adhesive tape to promote stabilization of anatomical structures. Angular limb deformities are a common problem affecting foals and is often treated surgically. The objective is to evaluate the efficacy of kinesiology taping as a treatment for angular limb deformities in foals.

**Materials and methods:** Two cases of angular limb deformities were studied: Case 1: A 30-day-old, Mangalarga filly, with carpus valgus on the right front limb. Case 2: A 60-day-old, Quarter Horse foal, with carpus valgus on the left front limb. In both cases, a 20 cm tape Vetkin Tape^®^ was applied with 30% stretch over the medial aspect of the affected limb to support the carpal colateral medial ligament. A 10 cm tape for the two anchors was wrapped over the ends of the tape to avoid detachment. The tape was changed every 5 days and the foals were reevaluated at 15 days of the treatment. The angles were measured in the pictures by ImageJ software.

**Results:** The degree of angular limb deformity improved in both cases during the 15-day treatment period. The angle of the carpus gradually approached a normal value from day 1 to day 15. The angle change in Case 1 was from 153 to 180 (Fig. 1), and in Case 2 was from 162 to 177 degrees (Fig. 2).

**Fig. 1:**
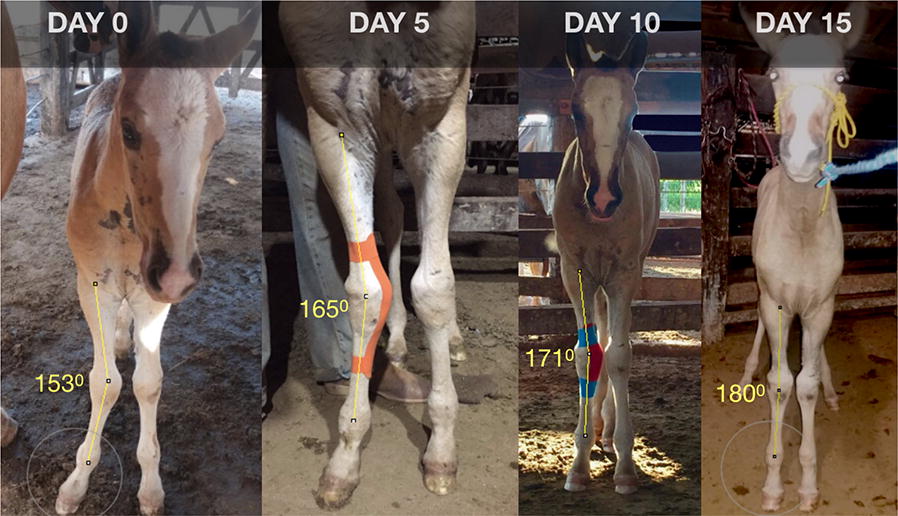
.

**Fig. 2:**
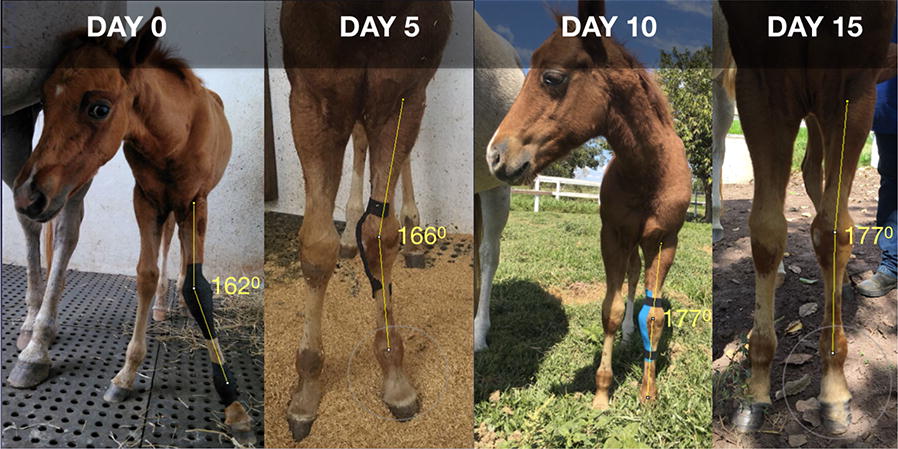
.

**Conclusion:** Kinesiology taping could be a potential non-invasive and inexpensive treatment for angular limb deformities in foals. Further research is warranted.

**References**

1. Karabicak GO, Bek N, Tiftikci U. Short-term effects of kinesiotaping on pain and joint alignment in conservative treatment of hallux valgus. J Manipulative Physiol Ther. 2015;38:564–71.
2. Colles CM. How to aid the correction of angular limb deformities in foals using physeal stimulation. AAEP Proceedings. 2008;54:60–3.

### A23 Rehabilitation for equine *Peroneus tertius* rupture

#### Solange Mikail, Laura Yasmin Stancov, Ana Lopes, Marina Villaça Issa de Araujo

##### Espaço Equus, Cotia, SP, Brazil

###### **Correspondence:** Solange Mikail (mikail@termovet.com.br)

*Acta Veterinaria Scandinavica* 2019, **61**(**Suppl 1**):A23

**Background:** A 17-year old Lusitano horse was evaluated at Espaço Equus Rehabilitation Center after a nine-month history of rest due to a rupture of the *Peroneus tertius* in the left hindlimb. Ultrasound examination at arrival showed an enlargement of the *Peroneus tertius* near its origin, with hypoechoic areas and diffuse edges. The horse presented with decreased maximal flexion (140 degrees) of the left tarsus during movement, as measured by a smart phone app (Hudl Technique) developed for biomechanical evaluation and measurement of joint angles during exercise.

**Materials and methods:** To establish the ability to flex the left tarsus, two treatments were implemented: Therapeutic laser (Respond System Luminex^®^) 20 J/cm^2^ to address the healing of the lesion and a program of exercises with progressive intensity to address the flexion of the tarsus and strengthen of the limb. The exercises consisted in stimulation of tarsal flexion, using different techniques over 1 month: gentle stimulation with a whip, hand walking, ridden exercises, tactile stimulator of the pastern, walking over poles, cavalettis and kinesiology tapping (VetkinTape^®^) during exercise.

**Results:** After 30 days, the ultrasound examination showed the *Peroneus tertius* with normal size and echogenicity and also well-defined edges. The biomechanical evaluation showed that the flexion of the tarsus improved substantially: the maximum flexion angle of the hock at walk went from 140° to 57°.

**Conclusion:** According to the literature the lesion of the *Peroneus tertius* takes several months to heal, and when near its origin, it carries a poor prognosis [1, 2]. Despite of the poor prognosis for the lesion site, this case has an excellent outcome in a short time (1 month) considering the previous time without evolution (9 months). This study highlights the value of adding a program of therapeutic exercises during the healing of this type of injury.

**References**

1. Koenig J, Cruz A, Genovese R, Fretz P, Trostle S. Rupture of the peroneus tertius tendon in 27 horses. Can Vet J. 2005;46:503–6.
2. Clayton HM, Kaiser LA, Stubbs NC. Hindlimb flexion responses to different types of tactile devices. Am J Vet Res. 2011;72:1489–95.

### A24 Proprioceptive innervation: What about the cranial ligament of the equine medial menisci? Preliminary results

#### Elodie Nemery^1^, Annick Gabriel^1^, Joëlle Piret^2^, Nadine Antoine^2^

##### ^1^ Laboratory of Veterinary Anatomy, FARAH Research Center, Faculty of Veterinary Medicine, University of Liège, Sart Tilman, Belgium; ^2^ Laboratory of Animal Histology, FARAH Research Center, Faculty of Veterinary Medicine, University of Liège, Sart Tilman, Belgium

###### **Correspondence:** Elodie Nemery (Elodie.Nemery@uliege.be)

*Acta Veterinaria Scandinavica* 2019, **61**(**Suppl 1**):A24

**Background:** Meniscal tears are common as stifle soft tissue injuries in the equine species. Cranial horn of the medial meniscus and the cranial meniscotibial ligament are the most often injured [1]. These lesions can be frequently accompanied by articular degenerative changes [2], resulting in poor sportive prognosis in the equine athlete [3].

In humans, it has been postulated that knee osteoarthrosis following meniscal injury could be due to biomechanical alteration [4] or proprioceptive disabilities attributable to the loss of mechanoreceptors (MCR), the role of the meniscus in joint stabilisation being not only mechanical [5].

In horses, a proprioceptive innervation has already been highlighted in the cranial horn of the medial menisci [6] but to our knowledge, until now, none studies have been performed in its cranial ligament.

Our objective was to highlight an eventual proprioceptive innervation in the equine cranial meniscotibial ligament of the medial menisci (Fig. 1).

**Fig. 1:**
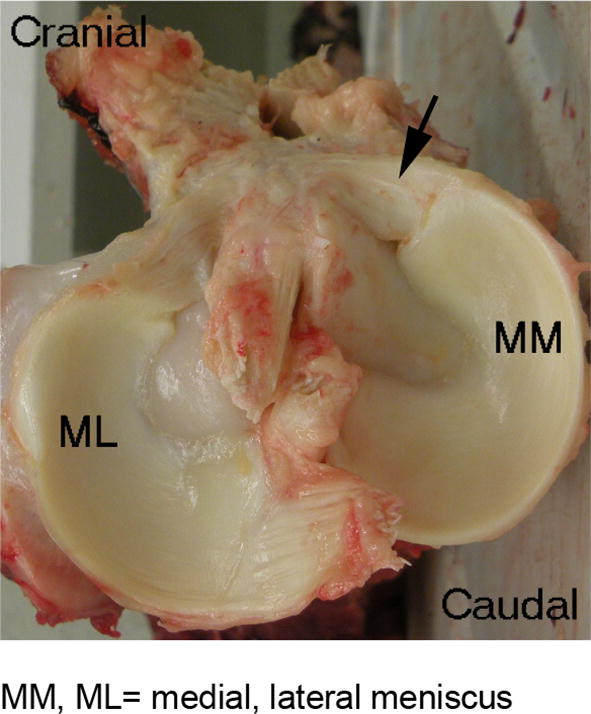
Anatomical location of the cranial ligament of the equine medial meniscus (black arrow). *MM, ML* medial, lateral meniscus

**Materials and methods:** Menisci were harvested, maximum 48 h post mortem, from one mare of 6 years old in an autopsy room. Death was not related with any locomotor problems. Serial cryosections were processed for immunohistochemistry and incubated in a range of primary antibodies directed against high molecular weight neurofilaments (anti-NFH), and Schwann cells (anti-GFAP). These antibodies were revealed with a species-specific secondary antibody bearing a fluorescent label. Nuclei were stained with DAPI.

Common criteria found in the literature, in order to identify the different types of mechanoreceptors, were included in Table 1 [6–10].

**Table 1:** Acoustic myographic variables of the longissimus dorsi in the saddle support area significantly (P < 0.05) affected by the rider (mean ± standard deviation)

	E left	E right	T left	T right	ESTI left	ESTI right
No rider	2.8 ± 2.7	1.5 ± 1.0	3.6 ± 2.8	2.6 ± 0.8	4.9 ± 2.0	3.9 ± 0.8
Rider	7.3 ± 2.5	7.0 ± 2.1	7.4 ± 1.3	7.4 ± 1.4	7.0 ± 1.2	6.8 ± 1.3

**Results:** On all sections analysed (n = 44), 36 nervous profiles were found with only one Golgi-like corpuscle observed (Fig. 2). As agreed with criteria found in Table 1, this corpuscle was found isolated. It exhibits a fusiform shape, shows a dense axonal arborisation with which Schwann cells established numerous close contacts. Corpuscle was about 279 µm long and 88 µm wide, estimated with Image J software.

**Fig. 2:**
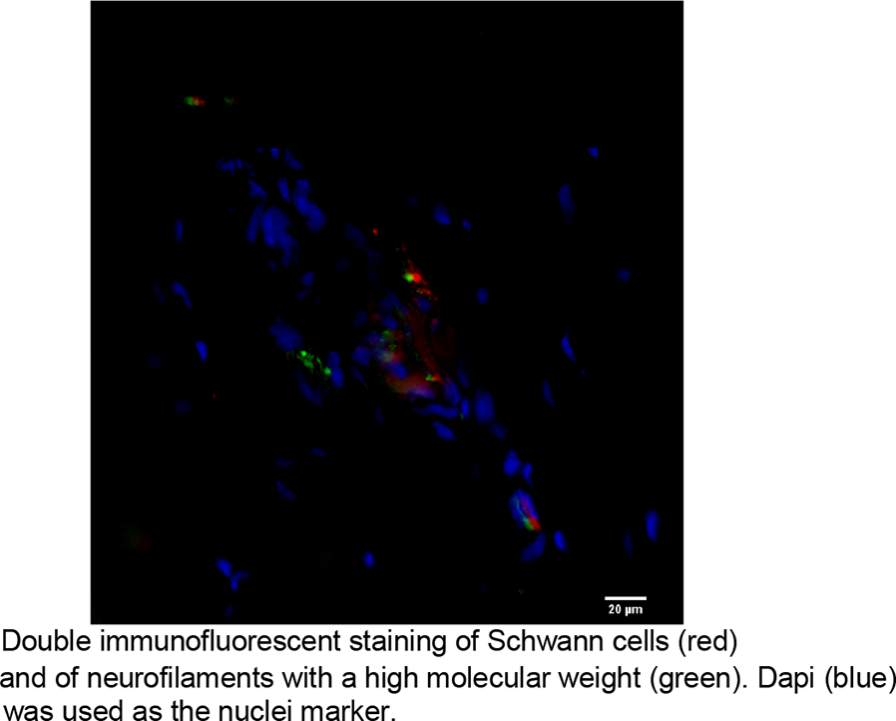
Type 3 corpuscle or Golgi-like corpuscle

**Table 1:**
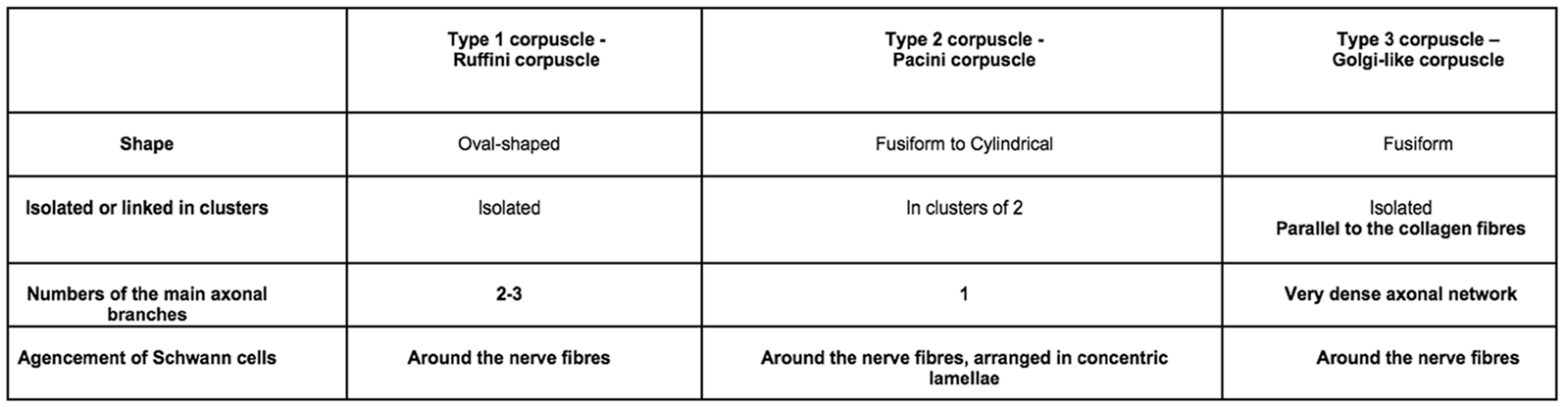
Criteria to identify mechanoreceptors based on literature review [6–10]

No Pacini, neither Ruffini were observed.

**Conclusion:** These preliminary results suggest that the cranial ligament of the equine medial menisci is innervated and possess a proprioceptive innervation. A young horse was chosen as it was postulated that number of MCR decrease with age [11]. Nevertheless in this horse, only one Golgi-like corpsucle was found; this MCR is active during extreme movement [12]. Increase of sample, followed by functional studies, would be interesting in order to assess the role of the proprioceptive innervation of this ligament in the equine stifle stability.

**Trial registration**: Not applicable. This is not a research study that “prospectively assigns human participants or groups of humans to one or more health-related interventions to evaluate the effects on health outcomes.”

**Acknowledgements:** We would like to express our special thanks to the staff of the autopsy room at the Faculty of Veterinary Medicine in Liège for the samples, to the technical assistance of Lemaitre O. and Weyckmans B. and to Cambroisier F.

**References**

1. Cohen JM, Richardson DW, McKnight AL, Ross MW, Boston RC. Long-term outcome in 44 horses with stifle lameness after arthroscopic exploration and debridement. Vet Surg. 2009;38:543–51.
2. Busscher V, Verwilghen D, Bolen G, Serteyn D, Busoni V. Meniscal damage diagnosed by ultrasonography in horses: a retrospective study of 74 femorotibial joint ultrasonographic examinations (2000–2005). J Equine Vet Sci. 2006;26:453–61.
3. Walmsley JP. Diagnosis and treatment of ligamentous and meniscal injuries in the equine stifle. Vet Clin North Am Equine Pract. 2005;21:651–72, vii.
4. Heijink A, Gomoll AH, Madry H, Drobnic M, Filardo G, Espregueira-Mendes J, et al. Biomechanical considerations in the pathogenesis of osteoarthritis of the knee. Knee Surg Sports Traumatol Arthrosc. 2012;20:423–35.
5. Karahan M, Kocaoglu B, Cabukoglu C, Akgun U, Nuran R. Effect of partial medial meniscectomy on the proprioceptive function of the knee. Arch Orthop Trauma Surg. 2010;130:427–31.
6. Nemery E, Gabriel A, Grulke S, Piret J, Toppets V, Antoine N. Mechanoreceptors in the Anterior Horn of the Equine Medial Meniscus: an immunohistochemical approach. Anat Histol Embryol. 2015;45:131–9.
7. Freeman MAR, Wyke B. The innervation of the knee joint: an anatomical and histological study in the cat. J Anat. 1967;101:505–32.
8. Halata Z. The ultrastructure of the sensory nerve endings in the articular capsule of the knee joint of the domestic cat (Ruffini corpuscles and Pacinian corpuscles). J Anat. 1977;124:717–29.
9. Halata Z, Rettig T, Schulze W. The ultrastructure of sensory nerve endings in the human knee joint capsule. Anat Embryol (Berl). 1985;172:265–75.
10. Halata Z, Haus J. The ultrastructure of sensory nerve endings in human anterior cruciate ligament. Anat Embryol (Berl). 1989;179:415–21.
11. Aydog ST, Korkusuz P, Doral MN, Tetik O, Demirel HA. Decrease in the numbers of mechanoreceptors in rabbit ACL: the effects of ageing. Knee Surg Sport Traumatol Arthrosc. 2006;14:325–9.
12. Cavalcante ML, Rodrigues CJ, Mattar Jr. R. Mechanoreceptors and nerve endings of the triangular fibrocartilage in the human wrist. J Hand Surg Am. 2004;29:432–8.

### A25 Spinal posture in horses with and without back pain

#### Gillian Tabor, Natacha Mann, Jane Williams

##### Equine Performance Research and Knowledge Exchange Arena, University Centre Hartpury, Gloucester, UK

###### **Correspondence:** Gillian Tabor (Gillian.tabor@hartpury.ac.uk)

*Acta Veterinaria Scandinavica* 2019, **61**(**Suppl 1**):A25

**Background:** Observation of posture by physiotherapists forms part of standard assessment procedure and includes visual analysis of spinal alignment, conformation and the relative position of thoracolumbar (TL) and lumbosacral (LS) spinal regions. A relationship between equine TL posture and back pathology has been reported following veterinary diagnostic imaging. However in clinical practice measurement using imaging e.g. radiographs is not practical. Objective measurement of posture using sagittal view photographs are considered reliable. This study aimed to determine if there were any differences in spinal posture between horses with and without back pain.

**Materials and methods:** Sagittal view digital photographs were taken of 71 horses (mean age 12.5 ± 6.2 years; mean height 155.2 ± 12.2 cm; 42 geldings and 29 mares), in a neutral square stance, with a smartphone camera (8 megapixels). TL and LS angles were measured from the photographs using ImageJ. TL angle was formed from the highest point of withers to the lowest dorsal aspect of the TL region to the tuber sacrale. A smaller TL angle related to relative extension compared to a larger TL angle. LS angle was formed from the lowest point of the dorsal aspect of the TL region to the tuber sacrale to the head of tail; a smaller LS angle related to relative flexion compared to a larger LS angle. Data were divided into back pain/no back pain groups based on assessment by a Chartered Physiotherapist and owner assessment, to determine if differences existed between horses with and without back pain (alpha: P < 0.05). Data were also explored for cluster analysis to examine for posture in sub groups.

**Results:** Horse with back pain had smaller TL angles (n = 38; mean 148.0 ± 3.20) than horses with no back pain (n = 33; mean 150.0 ± 3.80; t_70_ = 2.41, P = 0.019). There was no difference in the LS angle between groups (t_70_ = 1.33, P = 0.187). Cluster analysis identified four clusters and determined that horses with a larger TL and LS angle were less likely to have back pain.

**Conclusion:** Thoracolumbar extension is associated with over-riding dorsal spinous processes (ORDSP) and therefore it could be hypothesised that horses with a smaller TL angle are at increased risk of ORDSP or vice versa for larger TL angles. Diagnostic imaging would be required to confirm this. Assessment of posture should be included in equine physiotherapists’ assessment of horses with back pain. This simple method of objectively recording TL and LS angles could be used within routine assessment in clinical practice.

### A26 The use of outcome measures in equine rehabilitation

#### Gillian Tabor, Jane Williams

##### Equine Performance Research and Knowledge Exchange Arena, University Centre Hartpury, Gloucester, UK

###### **Correspondence:** Gillian Tabor (Gillian.tabor@hartpury.ac.uk)

*Acta Veterinaria Scandinavica* 2019, **61**(**Suppl 1**):A26

**Background:** The ideal goal of equine rehabilitation following injury or surgery is to return the horse to a level of function that either meets or exceeds the previous performance level and monitoring progress is important within rehabilitation. Outcome measures (OM) are used extensively in human practice and research, especially patient reported outcomes (PRO) and objective tests of clinical signs. PROs generally consist of a series of questions and observation of functional tasks, use of which may be challenging in equine practice. The aim of this study was to evaluate the knowledge of, and use, of OMs in equine musculoskeletal rehabilitation, to identify OMs used and clinician opinion regarding OM use in practice.

**Materials and methods:** An online questionnaire was used to investigate how those involved with the treatment and training of horses measure progress and outcomes during a rehabilitation. The questionnaire collected demographic data including qualification and during of experience within equine rehabilitation. The respondent was asked to define an OM and indicate their use, method of selection and analyzing OM data. The final section requested opinions on the benefits and barriers to OM use in equine rehabilitation and further comments.

**Results:** 107 practitioners responded, comprising 51 Chartered Physiotherapists and 20 Physiotherapists without prior human training, with an average of 9.25 years in equine practice. 82.2% reported using OMs. When asked to define an OM, 72.5% of Chartered Physiotherapists and 40% of Physiotherapists without prior human training, matched a pre-set definition correctly. The benefits of OM use were reported consistently as a method of objectively monitoring progress and used to adapt treatment plans. The barriers to OM use were lack of OM validation and reliability and time constraints. However the OMs reported to be used were mainly subjective such as visual assessment of lameness, palpation and observation of muscle symmetry.

**Conclusion:** Confusion exists regarding what an OM is and although OM use is reported it often refers to subjective assessment methods. Based on this information from a small group of professionals it would appear that there are limiting factors to the use of OMs in equine rehabilitation. This is primarily a lack of available OMs specific for equine rehabilitation. More work is needed to develop practically applicable outcome measures for specific professionals who are involved in the rehabilitation of equines.

### A27 Paralysis of a Shetland colt caused by drug intoxication resolved completely with Equiter^®^ Method of Equine Physical Therapy and Rehabilitation

#### Eliana Speziale

##### Equiter^®^ Center for Horse Rehabilitation, San Giuliano Terme, Tuscany, Italy

###### **Correspondence:** Eliana Speziale (elispez14@gmail.com)

*Acta Veterinaria Scandinavica* 2019, **61**(**Suppl 1**):A27

**Background:** Horses often suffer neurological damage due to poisonings caused by plants, feed additives, mycotoxins, herbal supplements, metal and minerals, or pesticides. In this case, a newborn colt was poisoned by a veterinary who administered an overdose (ten times the dose) of Depomycin im. (penicillin and streptomycin). The colt developed a fever, cough, and lost the ability to breathe just after birth. The colt was catatonic, could not stand in quadruped station, had no suck reflex, and did not indicate a desire to live.

**Materials and methods:** The colt’s lung infection and drug-induced intoxication was treated for 15 days with:

- Cobactan 4.5% ev (fourth generation cephalosporin)
- Meflosyl ev (anti-inflammatory)
- Selevit (Selenium, E vitamin, vitamin B12, etc.)
- Glucose Solution 5%
- Neatox
- Omeprazole

The colt was treated from a physical rehabilitation approach for progressive caudal-to-cranial rigidity due to progressive muscular atrophy generated by disuse and neurological damage. Atrophy caused by compression of the sciatic nerve caused medial dislocation of both patellae and hyperextension of the hind legs was also treated. The colt’s daily physical rehabilitation program followed Equiter^®^ procedure which is a structured mix of: deep massage therapy, myofascial release, structural digitopressure, muscles release, active and passive joint and body mobilization, exercises with poles and cavaletti and free exercise.

**Conclusion:** Cooperation between the veterinary therapies and Equiter^®^ Physical Therapy and Rehabilitation Program restored both proprioceptive and movement control in the 15 day old Shetland pony over approximately a two-month time frame.

## Exotics

### A28 Laser your dragon, stretch that gorilla, massage the tapir; applying rehabilitation techniques in a zoo setting

#### Harmony Frazier^1^, Kristin Kirkby Shaw^2^, Darin Collins^1^

##### ^1^Animal Health Department, Woodland Park Zoo, Seattle, WA, USA; ^2^SOUND Veterinary Rehabilitation Center, Shoreline, WA, USA

###### **Correspondence:** Harmony Frazier (harmony.frazier@zoo.org)

*Acta Veterinaria Scandinavica* 2019, **61**(**Suppl 1**):A28

With physical rehabilitation becoming an accepted part of comprehensive care in equine and companion animals, the zoo medicine community has also recognized the potential opportunities for this set of treatment modalities, due in large part to the success of animal training to allow cooperation and interaction. Woodland Park Zoo (WPZ) began exploring the benefits of physical rehabilitation for occasional patients in the 1980s. In 2012, WPZ developed a formal physical rehabilitation pilot program with the goal of increasing offerings throughout the zoo for animals with injuries, arthritis and mobility concerns. In order to objectively identify gait disturbances and track response to treatment, as well as create a common language across zoo teams, the Mobility Assessment Scoring System (M.A.S.S) was developed and adopted by the zoo staff. Based on common canine gait analysis scores, our M.A.S.S. stresses the application potential across species. In the 5 years of the pilot program, rehabilitation therapy has successfully been provided for a broad range of species ranging from lizards to gorillas. Based on the success of this pilot program, the WPZ Whole Health Initiative has been created as a collaboration between WPZ and SOUND Veterinary Rehabilitation Center as means of elevating the program with engagement of specialists in the field of veterinary rehabilitation. Goals of this initiative include advancing chronic pain management options for geriatric zoo animals and increasing the scope of rehabilitation services to include fitness and conditioning as a form of behavioral enrichment. With enthusiastic participation by zoo staff we have found our treatments to be effective across range of species.

### A29 Multimodal management of a non-human primate using analgesics, physical rehabilitation techniques and acupuncture

#### Mary Ellen Goldberg

##### International Veterinary Academy of Pain Management, Orlando, FL, USA

###### **Correspondence:** Mary Ellen Goldberg (mewhitester@gmail.com)

*Acta Veterinaria Scandinavica* 2019, **61**(**Suppl 1**):A29

**Background:** A 34-year-old Black-handed male, spider monkey (*Ateles geoffroyi*) had been suffering from chronic elbow dysplasia since 11/8/2008. In 4/2011, due to chronic disuse of the right arm and complete dislocation of the left arm, the subject’s condition was deteriorating and quality of life was threatened as he was unable to feed himself or play with conspecifics. The aim of the study was to test the use of analgesic pharmacology, physical rehabilitation methods, and acupuncture used in dogs, cats or horses on a non-human primate with physical limitations due to chronic bilateral elbow dysplasia.

**Materials and methods:** Long term pharmacological therapy was initiated in April 2011 followed by physical rehabilitation methods and acupuncture (Tables 1, 2, 3, 4). Daily, the animal keeper observed and recorded if the subject used his right and left arms to prehend food and, in conjunction with his prehensile tail, hang on trees and enrichment objects. If not observed, then the veterinary staff was notified. Charts for assessment of mobility, locomotion and adapted disability index were instituted (Tables 5 and 6).

**Table 1:** Pharmacologic therapy

Medication	Dosage	Administration	Frequency	Duration of therapy
Gabapentin supplied in 10/100/600 mg tablets	10–20 mg/kg	Orally mixed with food/treats	Twice daily	Until directed to stop
Glucosamine HCL 1500 mg + Chondroitin sulfate 350 mg capsule	1 capsule	Orally mixed with food/treats	Twice daily	Until directed to stop
Omega 3 fatty acids vitacaps™ capsule	1 capsule	Orally mixed with food/treats	Twice daily	Until directed to stop
Meloxicam tablets	0.1–0.2 mg/kg	Orally mixed with food/treats	Once daily	1 month
Polysulfated Glycosaminoglycan (Adequan^®^)	4.4 mg/kg	Intramuscular injection	Twice weekly	For 4 weeks, then subcutaneously every month and adjust frequency according to patient needs

**Table 2:** Therapeutic laser therapy

Model	Dosage	Area covered	Frequency	Duration of therapy
Cutting edge therapeutic laser EVO™	4–8 J/cm^2^	Humeral condyles of both right and left limbs	Once daily for 7 days, decreased to every other day during week 2	Applied on an as-needed basis

**Table 3:** Physical rehabilitation therapy

Manual therapy	Massage techniques	Hand weights	Therapy/medicine balls
Passive range of motion under sedation	Effleurage and petrissage under sedation	1–2-pound weider, neoprene covered usage through mimicry	Small physioball and 2-pound medicine ball Playing catch and throwing back
Active range of motion through mimicry	1st and 2nd week only	3 times weekly for 4 weeks	3 times weekly for 4 weeks

**Table 4:** Acupuncture treatment for elbow pain through a certified veterinary TCVM acupuncture veterinarian

Location	Qi-blood stagnation	Kidney Yang deficiency	Kidney Yin and Qi (or Yang) deficiency	Local points elbow area
Acupuncture points	TH-10, SI-8, *Zhou*-*shu, Yan*-*zhou, Cheng*-*deng,* SI-3, LI-4, LI-1, TH-1, TH-3	*Bai*-*hui, Jian*-*jiao,* BL-23, BL-11, BL-40, BL-60, GB-34, GB-39	KID-3, KID-10, BL-23, BL-26, *Shen*-*shu, Shen*-*peng, Shen*-*jiao,* LIV-3, SP-6, SP-9, ST-36, LI-10, and LI-11	LI-10, LI-11, LU-5, TH-10, SI-8, HT-3, *Zhou*-*shu* (elbow associate)
	^*^Bony *Bi* Syndrome	Combine with local points	Combine with local points	

Bony Bi syndrome is a very chronic stage of Bi syndrome in which the bones, including the spine, are affected. Hip dysplasia, degenerative joint disease (DJD), spondylosis, and intervertebral disc diseases (IVDD) are part of this syndrome

**Table 5:**
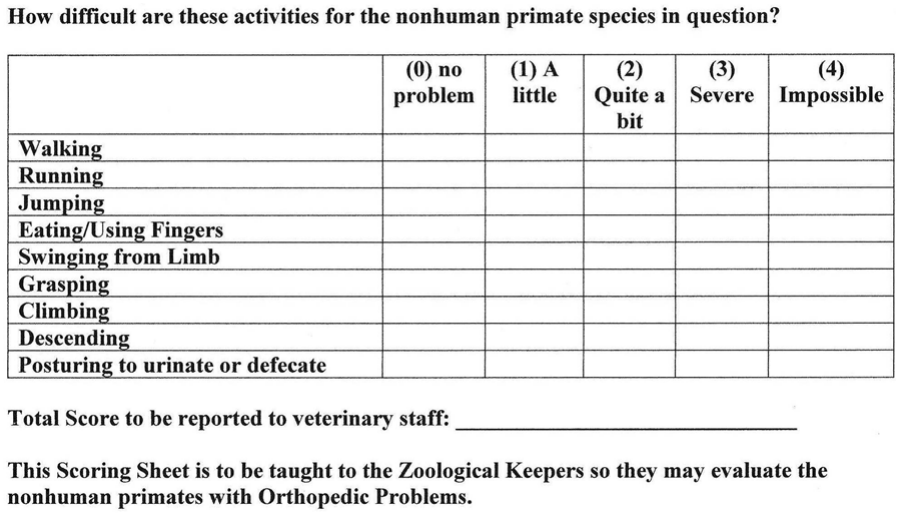
Modified cincinnati orthopedic disability index for a non-human primate

**Table 6:**
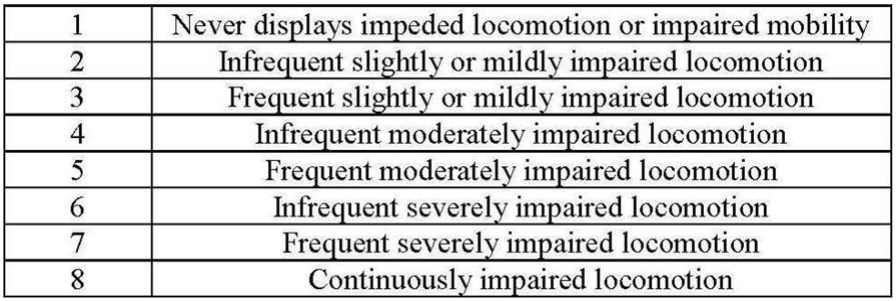
Scoring system for assessing mobility and locomotion for non-human primate

**Results:** With maintenance therapy, the spider monkey utilized arm and hand movements daily for feeding and play. He was observed functioning happily as a member of his troop.

**Conclusion:** The above treatment plan allowed veterinary staff and animal keepers to create an observable improvement in the spider monkey’s chronic pain without compromising his quality of life, therefore indicating multimodal analgesics, physical rehabilitation techniques and acupuncture can be effective treatments for spider-monkeys with chronic elbow dysplasia.

**References**

1. KuKanich B. Outpatient oral analgesics in dogs and cats beyond nonsteroidal antiinflammatory drugs: an evidence-based approach. Vet Clin North Am Small Anim Pract. 2013;43:1109–25.
2. Adkesson MJ. The role of gabapentin as an analgesic: potential applications in zoological medicine. In: Proceedings of the American Association of Zoo Veterinarians 2006; Tampa.
3. Wesselmann L, Cital SJ, Goldberg ME. Analgesia for zoo animal and wildlife practice. In: Goldberg ME Shaffran N, editors. Pain management for veterinary technicians and nurses. 1st ed. Ames, IA: John Wiley and Sons; 2015. p. 263–85.
4. Dadone L, Harrison T. Zoological applications of laser therapy. In: Riegel RJ and Godbold JC Jr., editors. Laser therapy in veterinary medicine: photobiomodulation. 1st ed. Ames, IA: John Wiley & Sons; 2017. p. 320–33.
5. Canapp S, Acciani D, Hulse D, Schulz K, Cannap D. Rehabilitation therapy for elbow disorders in dogs. Vet Surg. 2009;38:301–7.
6. Magden ER, Haller RL, Thiele EJ, Buchl SJ, Lambeth SP, Schapiro SJ. Acupuncture as an adjunct therapy for osteoarthritis in chimpanzees (*Pan troglodytes*). J Am Assoc Lab Anim Sci. 2013;52:475–80.
7. Cheng K. Basic learning. In: How animals think and feel: an introduction to non-human psychology. 1st ed. Santa Barbara, CA: Greenwood Publications; 2016. p. 24–25.

### A30 Rehabilitation of loss of lower extremity motor function in a guinea pig with pododermatitis

#### Kelli Martin^1^, David Levine^2^

##### ^1^Savannah Animal Hospital, 33818 Wescoats Road, Lewes, DE, USA; ^2^The University of Tennessee at Chattanooga, Chattanooga, TN, USA

###### **Correspondance:** Kelli Martin (kmartin@savannahanimalhosp.com)

*Acta Veterinaria Scandinavica* 2019, **61**(**Suppl 1**):A30

**Background:** Domestic guinea pigs (*Cavia porcellus*) are a common household pet in the US and rank in the top ten pets for ownership. Little is known about rehabilitation of injuries in this species.

**Case description**: The subject is a two-year-old intact male guinea pig. In September 2017, his owners reported he started to drag his hind legs for unknown reasons; no recent trauma was reported. He also presented with pododermatitis on his right hind leg. The subject had previously sustained a severe head injury in 2015, which had caused neurologic deficits; his owners stated he never fully recovered the use of his left hind leg. The initial evaluation revealed significant swelling and infection of the right hind leg, incontinence of bowel and bladder, and significant muscle atrophy in the hind limbs. The forelimbs were normal. Deep pain was present in both hind limbs, and pain was elicited on palpation over the lumbosacral spine. The patellar and withdrawal reflexes appeared to be decreased in strength, indicating a lower motor neuron lesion. Radiographs indicated no fractures and no narrowed disc spaces. The goals were to increase muscle mass, improve hind limb function, restore ambulation, and resolve the pododermatitis. Interventions included oral antibiotics (Baytril 100 mg/mL, 0.05 mL po every 24 h for 2 weeks) and anti-inflammatories (Metacam 0.5 mg/mL–0.3 mL po every 24 h) for inflammation and discomfort. PROM of both hind limbs, aquatic therapy, therapeutic laser to the sacral spine and hips (3.5 J/cm^2^), as well as bilateral tarsus in the area of pododermatitis (2.4 J/cm^2^), walking short distances, aquatic therapy (Fig. 1) and “dancing” (Fig. 2). Rehabilitation lasted 21 days for a total of 10 sessions.

**Fig. 1:**
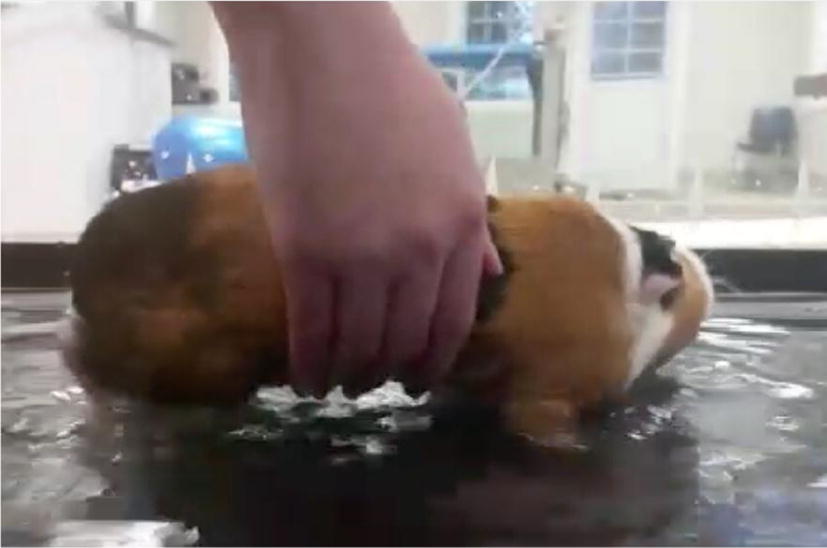
Aquatic therapy

**Fig. 2:**
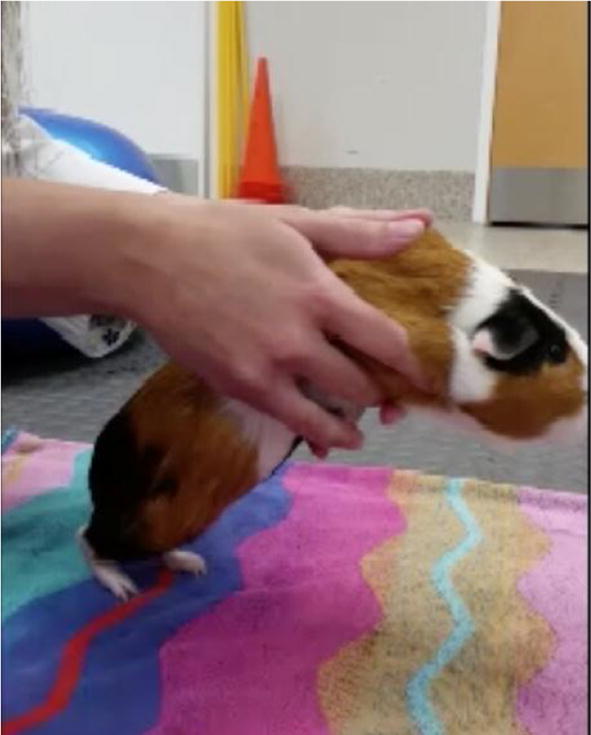
Dancing exercises

**Conclusion:** By discharge, the subject regained full range of motion, had no discomfort in any limb (Table 1), and was able to ambulate independently. Per subjective evaluation, the subject gained muscle over his spine and hind limbs. The pododermatitis also resolved.

**Table 1:** Scores of pain, lameness, and weight of subject at evaluation and discharge

	Initial evaluation	Discharge	Weight (g)
Lameness	4/4	0/4	900
Pain	2/4	0/4	1000

**Trial registration**: Not applicable.

**References**

1. U.S. Pet Ownership & Demographics Sourcebook (2012). American Veterinary Medical Association. https://www.avma.org/KB/Resources/Statistics/Pages/Market-research-statistics-US-pet-ownership.aspx.
2. White SD, Guzman DSM, Paul-Murphy J, Hawkins MG. Skin diseases in companion guinea pigs (*Cavia porcellus*): a retrospective study of 293 cases seen at the Veterinary Medical Teaching Hospital, University of California at Davis (1990–2015). Vet Dermatol. 2016;27:395–e100.
3. Bumblefoot BJ. A comparison of clinical presentation and treatment of pododermatitis in rabbits, rodents, and birds. Vet Clin North Am Exot Anim Pract. 2013;16:715–35.

### A31 Rehabilitation after strangulation injury to distal left forelimb in guinea pig

#### Michelle Meckelborg^1^, David Levine^2^

##### ^1^Park Veterinary Centre, Sherwood Park, AB, Canada; ^2^Department of Physical Therapy, University of Tennessee Chattanooga, Chattanooga, TN, USA

###### **Correspondance:** Michelle Meckelborg (mam2010@shaw.ca)

*Acta Veterinaria Scandinavica* 2019, **61**(**Suppl 1**):A31

**Background:** There is a paucity of literature on rehabilitation in domestic guinea pigs (*Cavia porcellus*).

**Case description:** A 4 year-old neutered male guinea pig with no significant past medical history beyond being overweight (1.314 g). A blood sample was collected from the guinea pig’s left cephalic vein, and a bandage placed on the limb post-phlebotomy. The bandage was accidentally left in place for 5 days, causing a tourniquet effect to the distal limb with subsequent soft tissue necrosis (Fig. 1). The guinea pig was lame in this left forelimb (4/4), lacked sensation distal to L elbow joint, and was in 7/10 pain at initial evaluation. The objective was to prevent amputation of the guinea pig’s left forelimb.

**Fig. 1:**
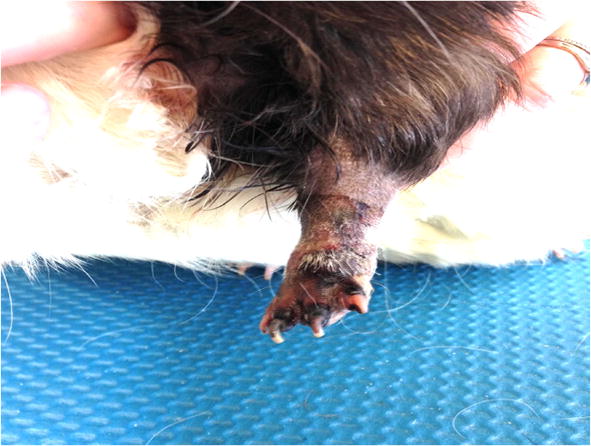
Forelimb 6 days after bandage was initially placed. It is swollen and edematous to roughly twice the size of the unaffected arm. There is a distinct demarcation at the elbow between necrotic and viable tissue

**Materials and methods:** Interventions included removal of the dressing, oral antibiotics (Trimethoprim sulfa 30 mg/kg PO BID) and NSAIDs (Metacam 0.3 mg/kg PO SID), surgical debridement of necrotic tissue as needed (Figs. 2a and b, 3), daily wet-to-dry dressings until wound showed healthy granulation tissue, dry dressings with silver sulfadiazine cream every other day to promote epithelialization, laser (3 J/cm^2^), hydrotherapy modalities, and proprioception training. Rehabilitation spanned 12 visits.

**Fig. 2:**
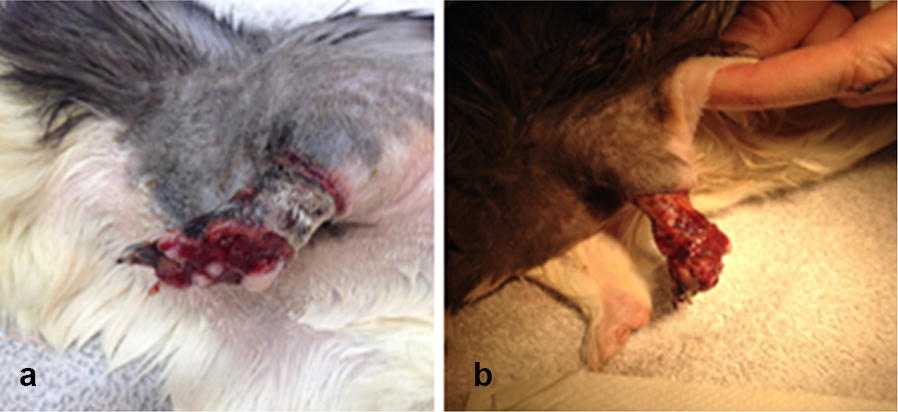
Forelimb prior to surgical debridement (**a**), and post-surgical debridement (**b**)

**Fig. 3:**
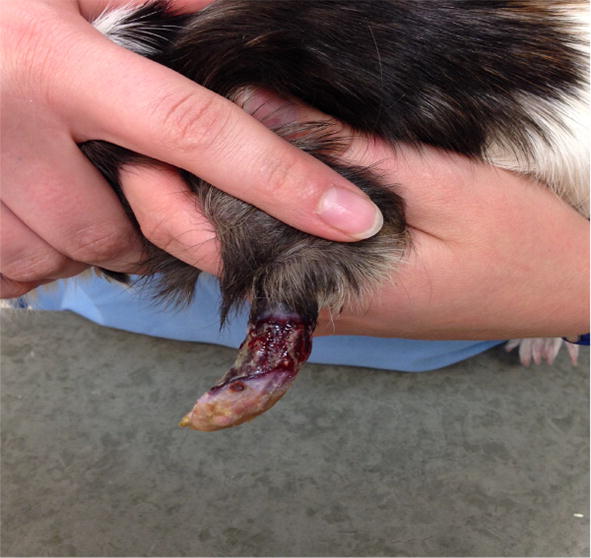
Forelimb 3.5 weeks post-surgical debridement

**Results:** The guinea pig was able to avoid amputation and experienced a return of all sensation in the left forelimb. By the end of treatment, he exhibited 1/4 pain. Lameness also decreased to 1/4.

**Conclusion:** After 12 sessions of rehabilitation, amputation of the left forelimb was avoided. Lameness decreased from 4/4 to 1/4.

**Trial registration:** Not applicable.

**References**

1. Parirokh M, Dabiri S, Bahrampour A, Homayon Zadeh M, Eghbal MJ. Effect of low power laser on incisional wound healing. Iran Endod J. 2006;1:45–8.
2. Peplow PV, Chung TY, Baxter GD. Laser photobiomodulation of wound healing: a review of experimental studies in mouse and rat animal models. Photomed Laser Surg. 2010;28:291–25.
3. Kontoes PP, Vrettou CP, Loupatatzi AN, Marayiannis KV, Foukas PG, Vlachos SP. Wound healing after laser skin resurfacing: the effect of a silver sulfadiazine-hyaluronic acid-containing cream under an occlusive dressing. J Cosmet Laser Ther. 2010;12:10–3.

### A32 Functional and nonfunctional overreaching induced anxiety and adrenosomatic index alteration in Wistar rats

#### Henriette G. Moranza^1^, Maria L. M. Almeida^1^, Gabriel Conde^1^, Júlia R. G. Carvalho^1^, Maria I. Mataqueiro^2^, Guilherme C. Ferraz^1^

##### ^1^Department of Animal Morphology and Physiology, Faculty of Agrarian and Veterinary Sciences, São Paulo State University (UNESP), Jaboticabal, SP, Brazil; ^2^Taquaritinguense Education Institute (ITES), Taquaritinga, SP, Brazil

###### **Correspondence:** Júlia R. G. Carvalho (juliargc@hotmail.com)

*Acta Veterinaria Scandinavica* 2019, **61**(**Suppl 1**):A32

**Background:** Elite athletes are exposed to high training loads and increasingly demanding competition calendars. Increases in training intensities are performed to improve performance [1], often exceeding the physical and psychological limit. The incorrect load management is an important factor for the development of overtraining syndrome (OTS) [4], caused by an imbalance in the cycle training-recovery, resulting in performance reduction [3] in human and equines. Behavioral indicators have great sensitivity and can be an effective tool for diagnosis/prevention of nonfunctional overreaching (NFOR) and OTS. Studies with rats submitted to overtraining protocols (OP) and behavioral disorders were not found in literature.

The objectives of the study were to investigate the effects of induction of functional overreaching (FOR) and NFOR on the behavior and adrenosomatic index of Wistar rats.

**Materials and methods:** Sixty-five rats composed four groups (SED: sedentary; SSED: semi sedentary; FOR and NFOR). Thirty-three animals underwent a 12-week OP, which was performed in a treadmill. In the first 8 weeks a daily training session was performed. In the last 4 weeks, there was an increase in training frequency (2, 3, 4 and 4 x/d) and reduction of recovery time between sessions (4, 3, 2 and 2 h). To evaluate performance, the rats were submitted to maximum performance tests (MPT). Elevated plus maze (EPM) and Open Field (OF) tests were performed at the end of the OP. Adrenosomatic index was measured. ANOVA (Repeated Measures and One-Way) and Pearson’s correlation were used for statistical analysis.

**Results:** FOR and NFOR started increasing performance in the MPT-2 (303.8 ± 13.8; 294.3 ± 18.9 kg.m, respectively) (Fig. 1). The NFOR group had reduced performance in MPT-7 (292.2 ± 36.4 kg.m). In OF, there was an increase in the frequency of rearing for the FOR and NFOR in relation to the SED group (F = 7.429; P = 0.006) and increase NFOR in relation to SSED (F = 7.429; P = 0.029) (Fig. 2). There was no difference in EPM. Adrenosomatic index increased for NFOR and FOR in MPT-7 in relation to the SED group (F = 7.205, P < 0.001) and a reduction of the NFOR index after MPT-9 was observed (P = 0.006), which correlated with performance in both moments (MPT-7: P = 0.03; R = − 0.754; MPT-9: P = 0.004; R = 0.94) (Fig. 3). MPT-8 and 9 data were not shown.

**Fig. 1:**
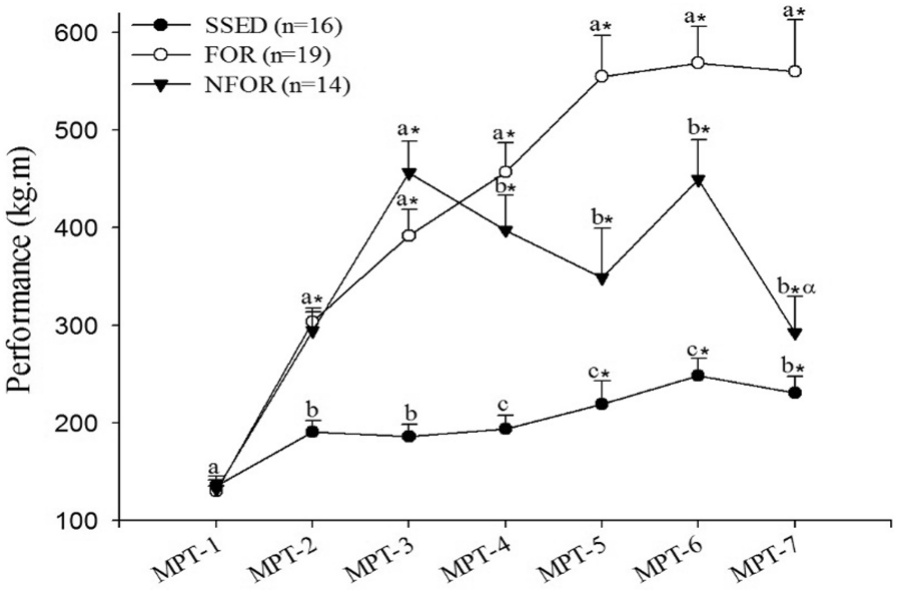
Performance of the groups in the maximum performance tests (MPT) during the OP. SSED: Semi sedentary; FOR: functional overreaching; NFOR=nonfunctional overreaching. *Indicates increase in intragroup performance in relation to MPT-1 (P < 0.05). α: Indicates performance reduction in relation to MPT-6 (P < 0.05). Different letters indicate differences between groups (P < 0.05)

**Fig. 2:**
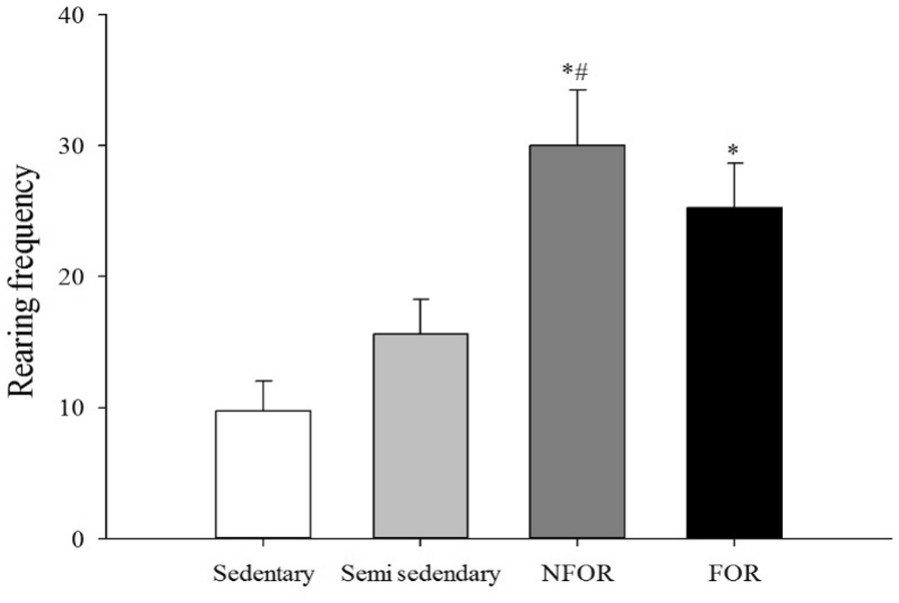
Rearing frequency in the Open field test. Data presented as mean ± SE. *Indicates increase in the rearing frequency in relation to group Sedentary (P < 0.05). ^#^Indicates increase in relation to the Semi sedentary group. Sedentary (n = 8); Semi sedentary (n = 8); FOR: functional overreaching (n = 11); NFOR; nonfunctional overreaching (n = 6)

**Fig. 3:**
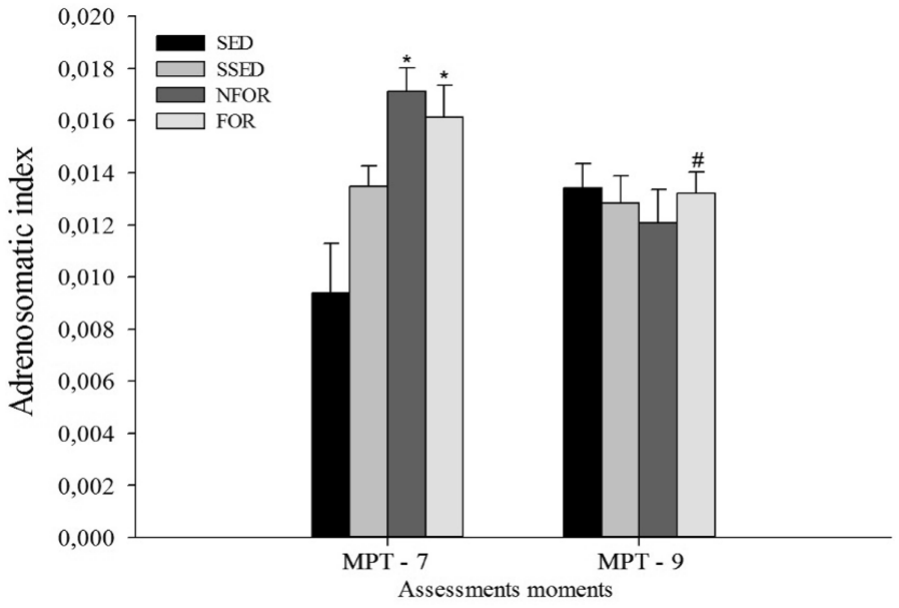
Adrenosomatic index. SED: sedentary group; SSED: semi sedentary group; FOR: functional overreaching; NFOR: nonfunctional overreaching. In MPT-7: SED, n = 8; SSED = 8; FOR, n = 8; NFOR, n = 8. In MPT-9: SED, n = 8; SSED = 8; FOR, n = 11; NFOR, n = 6. *Indicates an increase in relation to group SED in MPT-7 by the Holm-Sidak test (P < 0.05). ^#^Indicates a decrease of the NFOR group in MPT-9 relative to himself in MPT-7 by the t-test (P < 0.05). Data presented as mean ± SE

**Conclusion:** FOR or NFOR conditions induced anxiety and an increase in the adrenosomatic index was observed in the NFOR group.

Trial registration: Not applicable.

**Declaration**: The experimental protocol was approved by the Ethics Committee of the São Paulo State University (nº 23593/15) and followed the standards care of Use of Animals.

**Acknowledgements:** The São Paulo Research Foundation–FAPESP (process nº 2015/26738-1) and National Council for Scientific and Technological Development–CNPq (process nº 141287/2017-9).

**References**

1. Aubry A, Hausswirth C, Louis J, Coutts AJ, Buchheit M, Le Meur Y. The development of functional overreaching is associated with a faster heart rate recovery in endurance athletes. PloS One. 2015;10:1–16.
2. Kellmann M. Preventing overtraining in athletes in high‐intensity sports and stress/recovery monitoring. Scand J Med Sci Sports. 2010;20:95–102.
3. Meeusen R, Duclos M, Foster C, Fry A, Gleeson M, Nieman D, et al. Prevention, diagnosis, and treatment of the overtraining syndrome: joint consensus statement of the European College of Sports Science and the American College of Sports Medicine. Med Sci Sports Exerc. 2013;45:186–205.
4. Soligard T, Schwellnus M, Alonso JM, Bahr R, Clarsen B, Dijkstra HP, et al. How much is too much? (Part 1) International Olympic Committee consensus statement on load in sport and risk of injury. Br J Sports Med. 2016;50:1030–41.

### A33 Survival time of *Staphylococcus aureus* on therapeutic ultrasound heads and implications for rehabilitation

#### Kate L. Noonan^1^, Henry Spratt^1^, David Levine^2^

##### ^1^University of Tennessee at Chattanooga, Department of Biology, Geology, & Environmental Science, Chattanooga, TN, USA; ^2^University of Tennessee at Chattanooga, Department of Physical Therapy, Chattanooga, TN, USA

###### **Correspondence:** David Levine (David-Levine@utc.edu)

*Acta Veterinaria Scandinavica* 2019, **61**(**Suppl 1**):A33

**Background:** Therapeutic ultrasound (US) is commonly used for musculoskeletal injuries in companion animals [1, 2]. Both ultrasound heads and coupling gel containers are reused from patient to patient. These may serve as reservoirs for bacterial pathogens if the equipment is not disinfected between patients. It is also possible that bacteria may colonize the gel itself [3].

The purpose of this study was to determine the survivability of a potentially pathogenic bacterial species, *Staphylococcus aureus* on US heads. *S. aureus* is potentially pathogenic to animals undergoing US in a veterinary setting.

**Materials and methods:** Cultures of *S. aureus* were grown in tryptic soy broth (TSB) overnight. *S. aureus* was applied to sterile US heads either directly in 0.85% saline, or mixed into a matrix of organic matter (TSB at: 100%, 67%, and 33%). To test the survival of *S. aureus* in coupling gel, an aliquot of cells was applied directly to sterile Aquasonic gel added to US heads. After application to the heads, all mixtures were dried using filtered air. Samples from the US heads were obtained via sterile transport swabs, at 1 h, 24 h, and 72 h. These swabs were then immediately placed in a tube of sterile saline, followed by a serial dilution (Fig. 1) with plating to tryptic soy agar (TSA) plates for enumeration. These plates were incubated at 37 °C for 24 h and *S. aureus* colonies were counted. The method described was performed in triplicate.

**Fig. 1:**
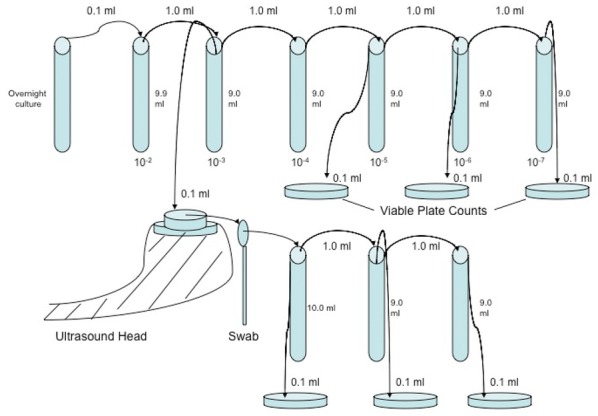
Representation of serial dilution used for dilution of *S. aureus* over night culture, addition to US heads, and counting of surviving cells

**Results:** Suspended in 0.85% saline, *S. aureus* did not survive 1 h. However, when mixed into varying concentrations of TSB or into coupling gel, significant numbers of *S. aureus* survived at least 1 h, and when mixed with TSB, up to 72 h (Fig. 2). Significant growth in the gel was also seen at 1 h, but showed 0% growth at 24 and 72 h.

**Fig. 2:**
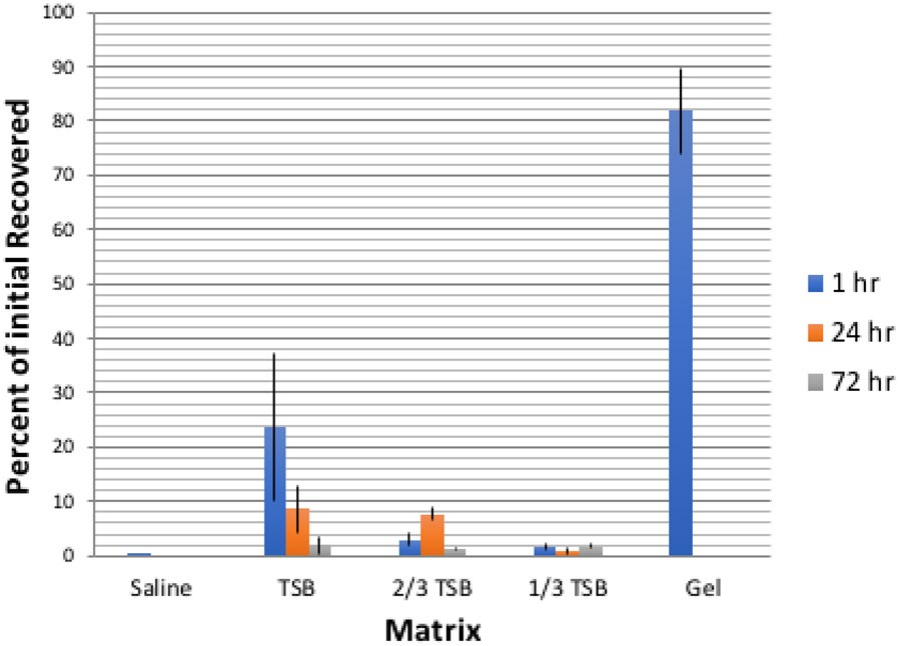
Percent survival of *S. Aureus* mixed into matrices or organic matter (TSB or Gel) of different concentrations over 72 hours

**Conclusion:** The findings of this study indicate that *S. aureus* has the potential to survive on aluminum US heads for up to 3 days when embedded in a matrix containing different concentrations of organic matter. Because US heads may come in contact with multiple animals in the course of a day, US heads must be disinfected thoroughly after use with each animal to reduce the potential of cross-contamination by bacteria such as *S. aureus*. The use of sterile gel may also help reduce cross contamination.

**References**

1. Levine D, Millis DL, Mynatt T. Effects of 3.3 MHz ultrasound on caudal thigh muscle temperature in dogs. Vet Surg. 2001;30:170–4.
2. Steiss JE, Adams CC. Effect of coat on rate of temperature increase in muscle during ultrasound treatment of dogs. Am J Vet Res. 1999;60:76–80.
3. Ohara T, Itoh Y, Itoh K. Ultrasound instruments as possible vector of staphylococcal infection. J Hosp Infect. 1998;40:73–7.

### A34 Immunoexpression analysis (IL-1β, IL-10 and Col II) of an experimental model of osteoarthritis in rats treated with photobiomodulation in association to chondroitin sulfate and glucosamine sulfate

#### Marcella Sanches^1^, Lívia Assis^1,2^, Cyntia Criniti^1^, Danilo Fernandes^1^, Carla Tim^1,2^, Ana Claudia Muniz Renno^1^

##### ^1^Department of Bioscience, Federal University of São Paulo, R. Silva Jardim, 136, Vila Mathias, Santos, SP, Brazil; ^2^Department of Biomedical Engineering, Brazil University, São Paulo, Brazil

###### **Correspondence:** Marcella Sanches (marcellasanches@gmail.com)

*Acta Veterinaria Scandinavica* 2019, **61**(**Suppl 1**):A34

**Background:** Osteoarthritis (OA) is a degenerative joint disease prevalent in the elderly animals and is very frequent in the knee joint. Currently, the therapeutic potential of photobiomodulation (FBM) and chondroprotectors, such as the glucosamine and chondroitin sulfates, is highlighted in the treatment of degenerative diseases affecting the joint cartilage [1, 2]. However there is no evidence of concomitant use of these treatments and what the possible interaction between them. Thus, the hypothesis supported was that the association of glucosamine and chondroitin sulfates to FBM may constitute a more effective therapeutic intervention to reduce inflammation in the joint cartilage associated with OA. In this way, the aim of this study was to compare the effects of combined treatment with chondroitin sulfate and glucosamine sulfate (CS/Gl) and photobiomodulation (PBM) on the degenerative process related to osteoarthritis (OA) in the articular cartilage in rats.

**Materials and methods:** Forty male Wistar rats were randomly divided into four groups: OA control group (CG); OAanimals submitted to PBM treatment (PBM); OA animals submitted to CS/Gl treatment (CS/Gl); OA submitted to CS/GS associated with PBM treatments (GS/Gl + PBM). The CS/Gl started 48 h after the surgery, and they were performed for 29 consecutive days. Moreover, PBM was performed after the CS/Gl administration on the left joint. Immunoexpression of interleukin 10 (IL-10) and 1 beta (IL-1β) and collagen type II (Col II) of the articular cartilage were evaluated.

**Results:** The results showed that CS/GS + PBM decreased IL-1β protein expression (P = 0.0359) (Fig. 1), increased IL-10 (P = 0.028) (Fig. 2) and Col II imunoexpression (P = 0.0204) (Fig. 3) compared to CG.

**Fig. 1:**
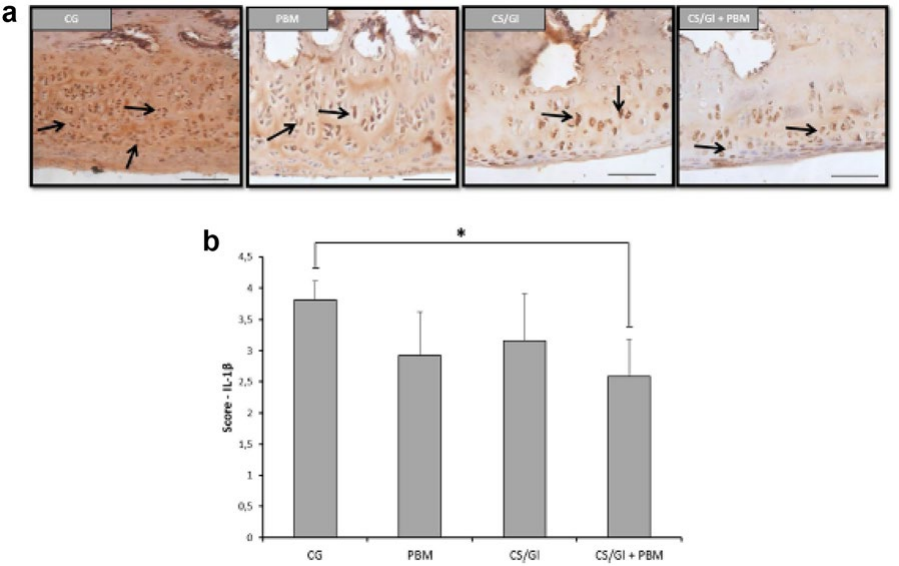
IL-1β imunoexpression. **a** Representative sections of IL-1β immunohistochemistry. Immunolabeled chondrocytes (arrow). OA control group (CG); OA animals submitted to PBM treatment (PBM); OA control group (CG); OA animals submitted to PBM treatment (PBM); OA animals submitted to CS/Gl treatment (CS/Gl); OA submitted to CS/Gl associated with PBM treatments (GS/Gl + PBM). (Scale Bar: 50 μm). **b** Results of semi-quantitative analysis of the IL-1β expression (indicated as *P < 0.05 versus CG). Results expressed as mean ± standard error of the mean

**Fig. 2:**
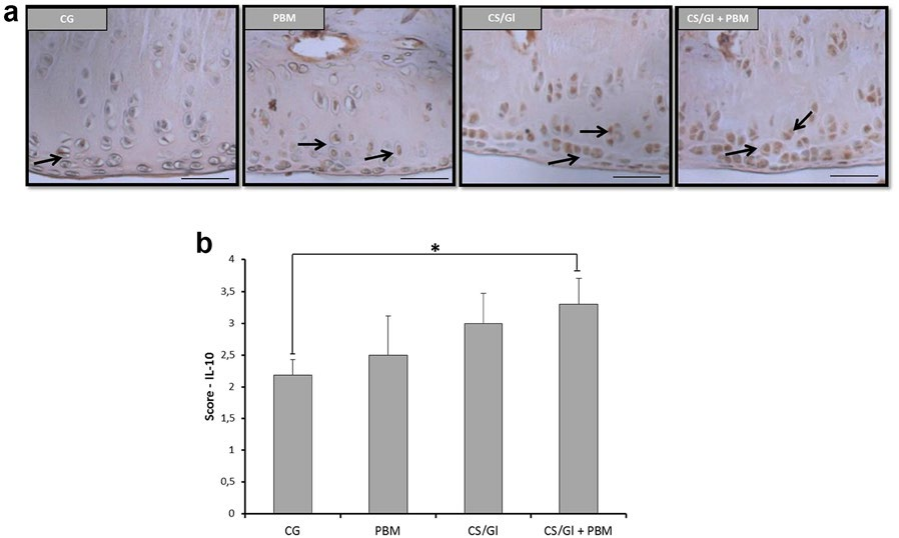
IL-10 imunoexpression. **a** Representative sections of IL-10 immunohistochemistry. Immunolabeled chondrocytes (arrow). OA control group (CG); OA animals submitted to PBM treatment (PBM); OA animals submitted to CS/Gl treatment (CS/Gl); OA submitted to CS/Gl associated with PBM treatments (GS/Gl + PBM). (Scale Bar: 50 μm). **b** Results of semi-quantitative analysis of the IL-10 expression (indicated as *P < 0.05 versus CG). Results expressed as mean ± standard error of the mean

**Fig. 3:**
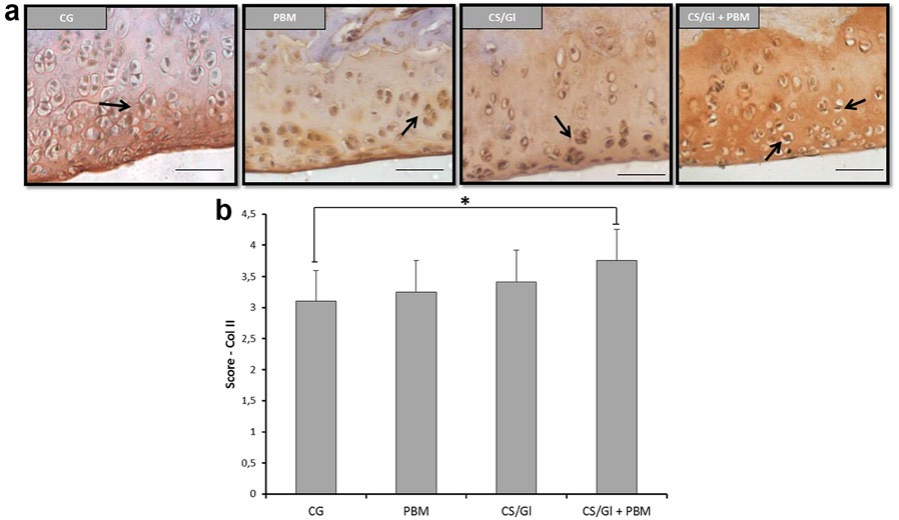
Collagen type II imunoexpression. **a** Representative sections of collagen II immunohistochemistry. Immunolabeled chondrocytes (arrow). OA control group (CG); OA animals submitted to PBM treatment (PBM); OA animals submitted to CS/Gl treatment (CS/Gl); OA submitted to CS/Gl associated with PBM treatments (GS/Gl + PBM). (Scale Bar: 50 μm). **b** Results of semi-quantitative analysis of the collagen II expression (indicated as *P < 0.05 versus CG). Results expressed as mean ± standard error of the mean

**Conclusion:** This study showed that CS/Gl associated with PBM was effective inmodulating inflammatory process and preventing the articular tissue degradation in the knees OA rats.

**References**

1. Bublitz C, Medalha C, Oliveira P, Assis L, Milares LP, Fernandes KR, et al. Low-level laser therapy prevents degenerative morphological changes in an experimental model of anterior cruciate ligament transection in rats. Lasers Med Sci. 2014;29:1669–78.
2. Henrotin Y, Lambert C. Chondroitin and glucosamine in the management of osteoarthritis: an update. Curr Rheumatol Rep. 2003;15:361.

## American College of Veterinary Sports Medicine and Rehabilitation (ACVSMR)

### A35 The effect of lumbar epaxial intramuscular injections on gait in working dogs

#### Carrie Elizabeth Ruge, Tracy Ann Darling, Cynthia Marie Otto

##### Penn Vet Working Dog Center, University of Pennsylvania, Philadelphia, PA, USA

###### **Correspondence:** Cynthia Marie Otto (cmotto@vet.upenn.edu)

*Acta Veterinaria Scandinavica* 2019, **61**(**Suppl 1**):A35

**Background:** Subtle gait abnormalities from intramuscular injections in working dogs could interfere with their ability to complete life-saving tasks safely and effectively. We hypothesized that intramuscular injection of naloxone into the lumbar epaxial muscles would not impact gait kinetics.

**Materials and methods:** This Institutional Animal Care and Use Committee approved cross-over study was conducted on 10 working dogs randomly assigned to opioid reversal with either 4 mg of naloxone intranasally or 4 mg of intramuscular naloxone (4 mL) in the left lumbar epaxial muscle. A right saphenous vein catheter was placed for administration of fentanyl (0.3 mg/dog) and was removed 120 min after opioid reversal. Gait analysis was performed using a temporospatial pressure sensing walkway (Gait4Dog; GaitRite, Sparta, NJ) prior to the study and 24 and 48 h after reversal. Data was analyzed by 2-way repeated measures ANOVA. All 10 dogs completed both arms of the study without incident.

**Results:** Independent of treatment group, in week 1, 48 h after catheter removal, the right hind had a significantly greater total pressure index (TPI) % and higher gait lameness score (GLS) than at 24 h. At 48 h, the mean stride length for 3 out of 4 limbs in the dogs treated with intramuscular naloxone was significantly shorter than the stride length when they were treated with intranasal naloxone.

**Conclusion:** Catheter placement can lead to regional pain and temporary changes in gait. Although no overt lameness was detected, the decrease in stride length could be the result of muscle soreness that could affect mobility, safety and performance of working dogs.

**Acknowledgements:** Funded in part by the Department of Homeland Security (HSHQDC-17-P-00112) and the U.S. Army Medical Research Institute of Chemical Defense (CRADA).

### A36 Pharmacokinetics, safety, and clinical efficacy of cannabidiol treatment in osteoarthritic dogs

#### Lauri-Jo Gamble^1^, Jordyn Boesch^1^, Christopher Frye^1^, Wayne Schwark^2^, Sabine Mann^3^, Lisa Wolfe^4^, Holly Brown^5^, Erin Berthelsen^1^, Joseph Wakshlag^1^

##### ^1^Department of Clinical Sciences, Cornell University College of Veterinary Medicine, Ithaca, NY, USA; ^2^Department of Molecular Medicine, Cornell University College of Veterinary Medicine, Ithaca, NY, USA; ^3^Department of Population Medicine, Cornell University College of Veterinary Medicine, Ithaca, NY, USA; ^4^Proteomic and Metabolomic Facility, Colorado State University, Fort Collins, CO, 80521, USA; ^5^Metzger Animal Hospital, State College, PA, USA

###### **Correspondence:** Joseph Wakshlag (jw37@cornell.edu)

*Acta Veterinaria Scandinavica* 2019, **61**(**Suppl 1**):A36

**Background:** Evidence for efficacity of cannabidiol (CBD) in lower vertebrates suggests potential immunomodulatory[1], anti-hyperalgesic [2, 3], antinociceptive [4, 5], and anti-inflammatory [6, 7], actions, making it an attractive therapeutic option in dogs with osteoarthritis (OA), yet minimal scientific evidence regarding safe and effective oral dosing exists. The objectives of this study were to determine basic oral pharmacokinetics, and assess safety and analgesic efficacy of a CBD based oil in dogs with OA.

**Materials and methods:** Single-dose pharmacokinetics was performed using two different doses of CBD enriched (2 mg/kg and 8 mg/kg) oil. Thereafter, a randomized placebo-controlled, double-blind, crossover study was conducted. Dogs received each of two treatments: CBD oil (2 mg/kg) or placebo oil every 12 h. Each treatment lasted for 4 weeks with a 2-week washout period. Baseline veterinary assessment and owner questionnaires were completed before initiating each treatment and at weeks 2 and 4. Hematology, serum chemistry and physical examinations were performed at each visit. A mixed model analysis of variance was utilized for all variables with a P value of < 0.05 deemed significant.

**Results:** Pharmacokinetics revealed an elimination half-life of 4.2 h at both doses and no observable side effects. Clinically, Canine Brief Pain Inventory and Hudson activity scores showed a significant decrease in pain and increase in activity (P < 0.001) with CBD oil. Veterinary assessment showed decreased pain during CBD treatment (P < 0.02). No side effects were reported by owners, however serum chemistry showed an increase in alkaline phosphatase during CBD treatment (P = 0.005).

**Conclusion:** This pharmacokinetic and clinical study suggests that 2 mg/kg of CBD twice daily can help increase comfort and activity in dogs with OA. The efficacy of this particular CBD product may not translate to other available products due to differing cannabinoid concentrations in this largely unregulated market.

**Acknowledgements:** ElleVet Sciences supported this research with a grant to Cornell University.

**References**

1. Sacerdote P, Martucci C, Vaccani A, Bariselli F, Panerai AE, Colombo A, et al. The nonpsychoactive component of marijuana cannabidiol modulates chemotaxis and IL-10 and IL-12 production of murine macrophages both in vivo and in vitro. J Neuroimmunol. 2005;159:97–105.
2. Costa B, Giagnoni G, Franke C, Trovato AE, Colleoni M. Vanilloid TRPV1 receptor mediates the antihyperalgesic effect of the nonpsychoactive cannabinoid, cannabidiol, in a rat model of acute inflammation. Br J Pharmacol. 2004;143:247–50.
3. Comelli F, Giagnoni G, Bettoni I, Colleoni M, Costa B. Antihyperalgesic effect of a Cannabis sativa extract in a rat model of neuropathic pain: mechanisms involved. Phytother Res. 2008;22:1017–24.
4. Shiue SJ, Peng HY, Lin CR, Wang SW, Rau RH, Cheng JK. Continuous intrathecal infusion of cannabinoid receptor agonists attenuates nerve ligation–induced pain in rats. Reg Anesth Pain Med. 2017; 42:499–506.
5. Cui JH, Kim WM, Lee HG, Kim YO, Kim CM, Yoon MH. Antinociceptive effect of intrathecal cannabinoid receptor agonist WIN 55,212-2 in a rat bone tumor pain model. Neurosci Lett. 2011;493:67–71.
6. Di Marzo V, Bifulco M, De Petrocellis L. The endocannabinoid system and its therapeutic exploitation. Nat Rev Drug Discov. 2004;3:771–84.
7. Malfait AM, Gallily R, Sumariwalla PF, Malik AS, Andreakos E, Mechoulam R, et al. The nonpsychoactive cannabis constituent cannabidiol is an oral anti-arthritic therapeutic in murine collagen-induced arthritis. Proc Natl Acad Sci U S A. 2000;97:9561–6.

### A37 Comparison of post-exercise cooling methods for dogs

#### Michael Scott Davis^1^, Denis J. Marcellin-Little^2^, Elizabeth O’Connor^2^

##### ^1^ Department of Physiological Sciences; ^2^ Center for Veterinary Health Sciences, Oklahoma State University-Stillwater, OK, USA

###### **Correspondence:** Michael Scott Davis (michael.davis@okstate.edu)

*Acta Veterinaria Scandinavica* 2019, **61**(**Suppl 1**):A37

Athletic dogs possess the capacity to produce large amounts of metabolic heat during exercise, but have a poor capacity for dissipation of that heat compared to other athletic mammals. As a result, overheating and heat related injury is a major cause of exercise-related morbidity in dogs. The objective of this study was to evaluate the relative efficacy of three post-exercise cooling methods in dogs with exercise-induced heat stress. Nine athletically-conditioned dogs were exercised at 10 km/h for 15 min on a treadmill in an environmental chamber at 30 °C three times on separate days. The dogs were cooled using one of three methods: Control cooling (consisting of resting in front of a fan), cooling on a 4 °C cooling mat, and partial immersion in a 30 °C water bath for 5 min. Ambient temperature during cooling was 30 °C, and dog temperature was recorded every 2 min using a radiotelemetry gastrointestinal capsule. Time-weighted heat stress (AUC of the temperature x time curve) was lower for immersion cooling compared to cooling mat and control. The mean duration required to lower gastrointestinal temperature to 39 °C was 16 min for immersion cooling, 36 min for cooling mat, and 48 min for control cooling. Immersion into a water bath decreases post-exercise time-weighted heat stress in dogs and provided the most rapid cooling of the three methods evaluated, even if the water temperature is relatively warm. The cooling mat was superior to cooling using only air movement, but not as effective as immersion.

### A38 Tissue heating and cooling properties of a contrast therapy device applied to the equine distal limb

#### Kevin Haussler, Shana Wolfer, Wayne McIlwraith

##### Gail Holmes Equine Orthopaedic Research Center, Department of Clinical Sciences, Colorado State University, Fort Collins, CO, USA

###### **Correspondence:** Kevin Haussler (Kevin.Haussler@ColoState.edu)

*Acta Veterinaria Scandinavica* 2019, **61**(**Suppl 1**):A38

Rehabilitation of tendon injuries often involves the application of cold therapy and heating is occasionally done to help increase collagen extensibility in fibrotic tissues. The application of alternating cold and hot (contrast therapy) is widely used in humans; however, its utility in equine rehabilitation is largely unknown. The objectives of this study were to evaluate the time–temperature profiles during 15 min heating/cooling cycles using an automated contrast therapy device, assess if the equipment could achieve therapeutic temperatures (< 15 °C and > 40 °C) surrounding the flexors tendons, and determine the effect of the sequence of therapy (i.e. hot–cold–hot versus cold–hot–cold). It was hypothesized that the superficial tissue structures would reach therapeutic temperatures during both heating and cooling cycles. Four adult horses with no diagnosed tendinopathies were used. Fine-wire temperature probes were placed on the skin and implanted in 3 locations within the metacarpus in both forelimbs: subcutaneously and deep to the superficial (SDFT) and deep digital flexor tendons (DDFT). Data was captured at 15 s intervals over 7 hot–cold cycles. Minimum and maximum temperatures, slopes and areas under the time–temperature curve were calculated. The applied contrast therapy was consistently able to apply therapeutic cold and heat to tissues superficial to the DDFT, as hypothesized. Temperatures deep to the DDFT were inconsistent, depending on the horse and the treatment cycle. These results help define the physiologic responses of combined tissue heating and cooling within the equine distal limb. Future studies are needed to define treatment protocols for optimal rehabilitation of distal limb injuries in horses.

**Acknowledgements:** Research funding was provided by the Equine Orthopaedic Research Fund. Student support was provided by the USDA Fellowship-USDA-NIFA Animal Health & Disease Research Program. Equipment was donated by Cascade Wellness.

**References**

1. Kaneps AJ. Tissue temperature response to hot and cold therapy in the metacarpal region of a horse. Proc Amer Assoc Equine Pract. 2000;46:208–213.
2. van Eps AW, Orsini JA. A comparison of seven methods for continuous therapeutic cooling of the equine digit. Equine Vet J. 2016;48:120–4.
3. van Eps AW, Pollitt CC, Underwood C, Medina-Torres CE, Goodwin WA, Belknap JK. Continuous digital hypothermia initiated after the onset of lameness prevents lamellar failure in the oligofructose laminitis model. Equine Vet J. 2014;46:625–30.
4. Bleakley CM, Costello JT. Do thermal agents affect range of movement and mechanical properties in soft tissues? A systematic review. Arch Phys Med Rehabil. 2012;94:149–63.
5. Versey NG, Halson SL, Dawson BT. Water immersion recovery for athletes: effect on exercise performance and practical recommendations. Sports Med. 2013;43:1101–30.
6. Menetrier A, Mourot L, Degano B, Bouhaddi M, Walther G, Regnard J, et al. Effects of three postexercice recovery treatments on femoral artery blood flow kinetics. J Sports Med Phys Fitness. 2015;55:258–66.

### A39 The use of venography with inclusion of the 60° dorsoproximal-palmarodistal oblique radiographic projection to evaluate perfusion in non-laminitic equine digit pathology

#### Kimberly Trolinger-Meadows^1^, Alison Morton^1^, Taralyn McCarrel^1^, Erin Gordon-Porter^2^, Vern Dryden^3^

##### ^1^ Department of Large Animal Clinical Sciences, University of Florida College of Veterinary Medicine, Gainesville, FL, USA; ^2^ Department of Small Animal Clinical Sciences, University of Florida College of Veterinary Medicine, Gainesville, FL, USA; ^3^Bur Oak Veterinary and Podiatry Services, Lexington, KY, USA

###### **Correspondence:** Kimberly Trolinger-Meadows (kmeadows@ufl.edu)

*Acta Veterinaria Scandinavica* 2019, **61**(**Suppl 1**):A39

**Background:** Radiography is routinely used to diagnose laminitic and non-laminitic conditions within the equine digit. To evaluate vascular disturbance, digital venography, including lateromedial and 0°-dorsopalmar projections, has been described for evaluation of horses with laminitis [1–8]. Digital venography has not been widely described in non-laminitic disease, nor has the use of additional projections for more complete evaluation of digital vasculature [7, 8]. The purposes of this study were to describe use of digital venography, including the lateromedial, 0°-dorsopalmar, and 60°-dorsoproximal-palmarodistal oblique projections, for evaluation of vascular disturbances in the digit of horses with non-laminitic disease of the digit; and to describe the use of the 60°-dorsoproximal-palmarodistal oblique projection with digital venography in normal equine digits.

**Materials and methods:** Medical records over a three-year period were reviewed to identify patients that had radiographic digital venography performed including lateromedial, 0°-dorsopalmar, and 60°-dorsoproximal-palmarodistal oblique projections for non-laminitic conditions of the digit. Radiographs were made prior to and following digital venography. Radiographic and venographic abnormalities were evaluated and compared to radiographic and venographic findings of five horses without disease of the digit. Normal venography was described on the 60°-dorsoproximal-palmarodistal oblique projections made from the control horses.

**Results:** Regions of altered perfusion were identified in five of eight horses with disease of the digit. The inclusion of the 60°-dorsoproximal-palmarodistal oblique projection allowed for more accurate localization of vascular disturbances of the digit.

**Conclusion:** Digital venography including the 60°-dorsoproximal-palmarodistal oblique projection should be considered for evaluation of vascular disturbance in horses with disease of the digit.

**References**

1. Lyle BE. Venography as a tool for guiding surgery to the foot. In: Floyd AD, Mansmann RA, editors. Equine podiatry. St Louis (MO): Saunders; 2007. p. 284–93.
2. Redding WR, O’Grady, SE. Nonseptic diseases associated with the hoof complex. Vet Clin North Am Equine Pract. 2012;28:407–21.
3. Rosenstein DS, Bowker RM, Bartlett PC. Digital angiography of the feet of horses. Am J Vet Res. 2000;61:255–9.
4. Redden R. A technique for performing digital venography in the standing horse. Equine Vet Educ. 2001;13:128–34.
5. Baldwin GI, Pollitt CC. Progression of venographic changes after experimentally induced laminitis. Vet Clin North Am Equine Pract. 2010;26:135–40.
6. D’Arpe L, Bernardini D. Digital venography in horses and its clinical application in Europe. Vet Clin North Am Equine Pract. 2010;26:339–59.
7. Rucker A. Equine venography and its clinical application in North America. Vet Clin North Am Equine Pract. 2010;26:167–77.
8. Rucker A. Clinical applications of digital venography. J Equine Vet Sci. 2010;30:491–503.

### A40 Clinical efficacy and mechanisms of action of a 2.5% polyacrylamide hydrogel in the treatment of osteoarthritis

#### Aziz Tnibar^1^, Hans Schougaard^2^, Linus Camitz^3^, Jonas Rasmussen^4^, Marc Koene^5^, Werner Jahn^6^, Bo Markussen^7^, Ann B. Persson^1^, Henrik E. Jensen^8^

##### ^1^Department of Large Animal Sciences, Faculty of Health and Medical Sciences, University of Copenhagen, Copenhagen, Denmark; ^2^Nørlund Hestehospital, Them, Denmark; ^3^Kasernens Hesteklinik, Næstved, Denmark; ^4^Højgård Hestehospital, Morud, Denmark; ^5^Tierärztlische Klinik für Pferde, Lüsche, Germany; ^6^Pferdeklinik Bargteheide, Bargteheide, Germany; ^7^Laboratory of Applied Statistics, Department of Mathematical Sciences, Faculty of Health and Medical Sciences, University of Copenhagen, Copenhagen, Denmark; ^8^Department of Veterinary Disease Biology, Faculty of Health and Medical Sciences, University of Copenhagen, Copenhagen, Denmark

###### **Correspondence:** Aziz Tnibar (aztnibar@gmail.com)

*Acta Veterinaria Scandinavica* 2019, **61**(**Suppl 1**):A40

**Background:** Polyacrylamide hydrogel (PAAG)^a^ was recently used to successfully treat osteoarthritis (OA) in horses; however no long-term field-study was performed. We hypothesized that lameness scores would improve significantly in OA joints after treatment with PAAG, and that PAAG has a lasting effect. Another objective was to describe the preliminary observations of the mechanisms of action of a PAAG in OA joints.

**Materials and methods:** Forty-three horses with OA in one joint were included in this study [1]. Horses were injected with 2 ml of PAAG into the affected joint and were followed up at 1, 3, 6, 12 and 24 months. Efficacy of PAAG was evaluated by blinded clinical assessment of lameness [1].

For the mechanisms of action of PAAG in OA joints, a randomized controlled study was conducted on an OA knee model in goats: treatment group (intraarticular PAAG), control group (intraarticular saline) [2, 3]. Magnetic Resonance Imaging (MRI) was performed prior and post-surgery [2, 3]. Gross pathology and histopathology, including immunohistochemistry for nerve endings, were performed on both knees [2, 3]. Joint capsule elasticity of the knees was measured on both groups [2, 3].

**Results:** At 1, 3, 6, 12 and 24 months follow-up, 59%, 69%, 79%, 81% and 82.5% of horses were non-lame respectively [1]. There was a significant decrease in lameness grade from baseline to 1, 3, 6, 12 and 24 months [1].

For the mechanisms of action, MRI showed reduction followed by stabilization of OA lesions after PAAG treatment [2, 3]. Histopathology showed that intraarticular PAAG injection added to the thickness of the synovial membrane; PAAG was integrated into the synovial membrane [2, 3]. Nerve endings were intact with normal morphology and numbers [2, 3]. Joint capsule elasticity investigation showed that treated knees had a higher elasticity when compared to control knees [2, 3].

**Conclusion:** PAAG significantly alleviated lameness in OA joints. No adverse effects were observed [1]. PAAG induced a significant decrease in joint effusion in OA joints. PAAG is a promising, lasting and safe new treatment for OA in horses.

Preliminary observations of the mechanisms of action of PAAG on OA joints were: 1. Pathology and joint capsule elasticity suggest that PAAG by acting on synovial membrane may reduce overall joint capsule stiffness, a major source of pain in OA. 2. MRI and pathology revealed stabilization of OA lesions in PAAG treated goats, possibly caused by the high viscosupplementation of PAAG.

**Trial registration** Danish Council for Animal Experimentation: 2010/561-1890 and 2011/561-2021.

**References**

1. Tnibar A, Schougaard H, Camitz L, Rasmussen J, Koene M, Jahn W, et al. An international multi-centre prospective study on the efficacy of an intraarticular polyacrylamide hydrogel in horses with osteoarthritis: a 24 months follow-up. Acta Vet Scand. 2015;57:20–7.
2. Tnibar A, Persson AB, Nielsen H, Svalastoga E, Westrup U, McEvoy F, et al. Evaluation of a polyacrylamide hydrogel in the treatment of induced osteoarthritis in a goat model: a randomized controlled pilot study. Osteoarthr Cartilage. 2014;22:477.
3. Tnibar A, Persson AB, Jensen HE. Mechanisms of action of an intraarticular 2.5% polyacrylamide hydrogel (Arthramid Vet) in a goat model of osteoarthritis: preliminary observations. SM J Biomed Eng. 2017; 3:1022

## American Association of Rehabilitation Veterinarians (AARV)

### A41 A survey of risk factors for digit injuries among dogs training and competing in agility events

#### Debra C. Sellon^1^, Katherine Martucci^1^, John R. Wenz^1^, Denis J. Marcellin-Little^2^, Michelle Powers^3^, Kimberley L. Cullen^4^

##### ^1^Department of Veterinary Clinical Sciences College of Veterinary Medicine Washington State University, Pullman, WA, USA; ^2^ Department of Surgical and Radiological Sciences, School of Veterinary Medicine, University of California-Davis, Davis, CA, USA; ^3^ Massachusetts Veterinary Referral Hospital, Woburn, MA, USA; ^4^ Institute for Work and Health Toronto, ON, Canada

###### **Correspondence:** Michelle Powers (mpowers@ethosvet.com)

*Acta Veterinaria Scandinavica* 2019, **61**(**Suppl 1**):A41

**Background:** Agility dog competitions have consistently increased over the last 20 years; currently over a million dogs per year participate in agility events [1]. According to results of a prior international survey of 3801 dogs, handlers indicated that approximately one-third of agility dogs experienced at least 1 injury during their competitive career [2, 3, 4, 5]. The most commonly reported sites of injury in affected dogs are the shoulders, back, neck, and digits. Injuries to the digits or paws have been estimated to represent 13% to 24% of total injuries in agility dogs [2–4]. In training and competing for agility events, specific tasks such as going over an A-frame contact obstacle have been found to be significantly associated with digit injuries [4]. However, other risk factors for injury in this population of sporting dogs are currently unknown. Therefore, the objectives of the study were to investigate potential risk factors for agility-related digit injuries in agility dogs and to characterize these injuries when present.

**Materials and methods:** Two retrospective electronic surveys were used to identify potential risk factors for digit injuries in dogs participating in agility. Surveys were distributed through social media sites and email lists related to canine agility. Variables evaluated included demographic information for handlers and dogs, physical characteristics of dogs, and descriptions of the type and possible causes of injury. Data were analyzed for association of individual variables with digit trauma. Multivariable logistic regression was used to develop a model of relevant risk factors.

**Results:** Data were collected from 207 handlers of agility dogs with digit injuries and 874 handlers of agility dogs without digit injuries. Factors associated with increased odds of injury included Border Collie breed (OR, 2.3), medium to long nails (OR, 2.3), absence of front dewclaws (OR, 1.9), and greater weight to height ratio (OR, 3.9). Odds of injury decreased with increasing age of the dog (OR, 0.8).

**Conclusion:** Several factors were associated with increased risk of digit injury in agility dogs suggesting that agility dogs should not have healthy dewclaws removed, should maintain lean body mass, and should have nails trimmed short for training and competition. Future biomechanical research and prospective clinical studies are important to confirm training and environmental recommendations to decrease risks of injury.

**References**

1. Agility: Annual and mid-year statistics. End-of-year MACH totals for 2014–2015. American Kennel Club. http://www.akc.org/events/agility/statistics/.
2. Cullen KL, Dickey JP, Bent LR, Thomason JJ, Moens NM. Internet-based survey of the nature and perceived causes of injury to dogs participating in agility training and competition events. J Am Vet Med Assoc. 2013;243:1010–8.
3. Kerr Zy, Fields S, Comstock RD. Epidemiology of injury among handlers and dogs competing in the sport of agility. J Phys Act Health. 2014;11:1032–40.
4. Levy M, Hall C, Trentacosta N, Percival M. A preliminary retrospective survey of injuries occurring in dogs participating in canine agility. Vet Comp Orthop Traumatol. 2009;22:321–4.
5. Fowler D. Distal limb and paw injuries. Vet Clin North Am Small Anim Pract. 2006;36:819–45.
6. Cullen KL, Dickey JP, Bent LR, Thomason JJ, Moens NM. Survey-based analysis of risk factors for injury among dogs participating in agility training and competition events. J Am Vet Med Assoc. 2013;243:1019–24.

### A42 How weight shifting affects load in canine rehabilitation exercises: a pilot study

#### Leslie Eide

##### Sound Veterinary Rehabilitation Center, Seattle, WA, USA

###### **Correspondence:** Leslie Eide (drleslie@animalsurgical.com)

*Acta Veterinaria Scandinavica* 2019, **61**(**Suppl 1**):A42

**Background:** Therapeutic exercise plays an important role in injury and surgical recovery [1]. Weight-bearing activities and isometric strengthening are key principles in an exercise program. As the body adapts to each exercise, changes must be made to the exercise to increase the stress placed on the muscle for continued strengthening. Changes in variables are used to regress or progress exercise such as: the number of repetitions, resistance, load, and other possible variables. Load is defined as a weight or source of pressure borne by someone or something [2]. In human exercise programs, weights and resistance bands can be used to change load, but this is often difficult to incorporate with dogs [3]. Instead, rehabilitation therapists have assumed weight-shifting can be used to change the load, but little is known about the degree of weight-shift needed to change the load. The assumption has also been made that raising the thoracic limbs causes a weight-shift to the pelvic limbs. This study looks to develop standard principles for load-altering exercises based on anatomical landmarks to use in rehabilitation exercise programs.

**Materials and methods:** The purpose of this study is to determine what degree of weight-shift is needed to affect load and to quantify how much load is affected. The study examined single thoracic leg lifts versus raising both thoracic limbs in medium to large breed dogs. Bilateral limb raising occurred at 2 different heights: approximately the height of the dog’s carpus and approximately the height of the elbow for the thoracic limbs. A commercially available stance analyzer is used to determine percentage of weight-shifting on all 4 limbs.

**Results:** Based on a small number of dogs (5) tested so far, it is noted that there is a change in load during weight-shifting exercises. The lifting of an individual thoracic limb shows almost all the load (95%) is shifted to the opposite thoracic limb. Raising the thoracic limbs to the height of the carpus does not significantly change to the load to the pelvic limbs, while raising to the height of the elbow does significantly change the load to the pelvic limbs.

**Conclusion:** Weight-shifting can be used to increase load on specific limbs. When increasing load to the pelvic limbs, thoracic limbs must be raised to the level of the elbows for a significant weight shift to occur.

**References**

1. Phelps HA, Ramos V, Shires PK, Werre SR. The effect of measurement method on static weight distribution to all legs dogs using the quadruped biofeedback system. Vet Comp Ortho Traumatol. 2007;20:108–12.
2. Cole GL, Millis D. The effect of limb amputation on standing weight distribution in the remaining three limbs in dogs. Vet Comp Ortho Traumatol. 2017;30:59–61.
3. DeLorme TL. Restoration of muscle power by heavy-resistance exercises. J Bone Joint Surg. 1945;27:645.

### A43 Retrospective analysis of two different doses of photobiomodulation combined with rehabilitation therapy as a therapeutic protocol for canine degenerative myelopathy

#### Lisa A. Miller^1^, Debbie (Gross) Torraca^2^, Luis De Taboada^3^

##### ^1,3^Companion Animal Health, LiteCure LLC, Newark, DE, USA; ^2^Wizard of Paws Physical Rehabilitation for Animals, Colchester, CT, USA

###### **Correspondence:** Luis De Taboada (luisd@litecure.com)

*Acta Veterinaria Scandinavica* 2019, **61**(**Suppl 1**):A43

**Background:** Canine degenerative myelopathy (DM) is a progressive neurodegenerative disease for which there exists a dearth of effective treatments as well as published historical data sets on disease progression or survival from the time of symptom onset (Sym Onset). Clinicians usually pursue symptom palliation using novel therapies alone or in combination with physiotherapy.

The objective of this study was to retrospectively examine the impact that adding photobiomodulation therapy (PBMt) to intensive rehabilitation therapy had on the progression of clinical signs of DM on patients treated at a single specialty rehabilitation facility.

**Materials and methods:** Clinical records of dogs referred to the facility with DM symptoms between 2003 and 2012 were screened for patients meeting prospectively identified inclusion and exclusion criteria. Patients meeting criteria (n = 20) were divided into two groups, based on the PBMt dose used: a low dose (LD) group (n = 6) and high dose (HD) group (n = 14). Items related to demographics, diagnostics, rehabilitation protocols, and time of progression of clinical signs from onset of symptoms (Sym Onset) to non-ambulatory paresis (NAP) or paralysis, and to euthanasia, were collected. Data was analyzed to determine differences in outcomes between the HD and LD groups, and historical expectations as given by previous published studies.

**Results:** The mean time between the Sym Onset and NAP was 8.79 ± 1.60 months (mean ± SD) in the LD group, and 31.76 ± 12.53 months in the HD group. The difference was significant (P < 0.05). The mean time between Sym Onset and time of euthanasia was 11.09 ± 2.68 months in the LD group, and 38.2 ± 14.67 months in the HD group. The difference was significant (P < 0.05). Kaplan–Meier survival analysis was used to compare time from Sym Onset to NAP for the LD and HD groups, and a singular historical published study with sufficient data, showed that the time from Sym Onset to NAP for the HD group was significantly longer than the LD group (P < 0.05) or the historical group (P < 0.05). The authors acknowledge the limitations of a retrospective review.

**Conclusion:** The data reviewed shows a significant difference in progression from Sym Onset to NAP and to time of euthanasia between these two PBMt dosage groups and is suggestive of a similar difference between the HD group and reviewed historical data. Further studies are warranted.

